# Whole-genome sequencing identifies novel predictors for hematopoietic cell transplant outcomes for patients with myelodysplastic syndrome: a CIBMTR study

**DOI:** 10.1186/s13045-023-01431-7

**Published:** 2023-04-11

**Authors:** Tao Zhang, Paul Auer, Jing Dong, Corey Cutler, Amy E. Dezern, Shahinaz M. Gadalla, H. Joachim Deeg, Aziz Nazha, Karen-Sue Carlson, Stephen Spellman, Yung-Tsi Bolon, Wael Saber

**Affiliations:** 1 CIBMTR® (Center for International Blood and Marrow Transplant Research), National Marrow Donor Program/Be The Match, Minneapolis, MN USA; 2 Division of Biostatistics, Institute for Health and Equity, Medical College of Wisconsin, Milwaukee, WI USA; 3 CIBMTR® (Center for International Blood and Marrow Transplant Research), Department of Medicine, Medical College of Wisconsin, 9200 W Wisconsin Ave, Milwaukee, WI 53226 USA; 4 Cancer Center Biostatistics Shared Resource, Medical College of Wisconsin, Milwaukee, WI USA; 5 Division of Hematology Oncology, Department of Medicine, Medical College of Wisconsin, Milwaukee, WI USA; 6 Medical College of Wisconsin Cancer Center, Milwaukee, WI USA; 7 Linda T. and John A. Mellowes Center for Genomic Sciences and Precision Medicine, Medical College of Wisconsin, Milwaukee, WI USA; 8 Stem Cell Transplantation and Cellular Therapy, Dana-Farber Cancer Institute, Boston, MA USA; 9 The Sidney Kimmel Comprehensive Cancer Center, Johns Hopkins Medicine, Baltimore, MD USA; 10 Division of Cancer Epidemiology & Genetics, NIH-NCI Clinical Genetics Branch, Rockville, MD USA; 11 Clinical Research Division, Fred Hutchinson Cancer Research Center, Seattle, WA USA; 12 Cleveland Clinic Foundation, Cleveland, OH USA; 13 Medical College of Wisconsin, Milwaukee, WI USA; 14 Blood Research Institute, Versiti, Milwaukee, WI USA

**Keywords:** Myelodysplastic syndrome, WGS, Whole-genome sequencing, Post-transplant survival outcome, *TP53*

## Abstract

Recurrent mutations in TP53, RAS pathway and JAK2 genes were shown to be highly prognostic of allogeneic hematopoietic cell transplant (alloHCT) outcomes in myelodysplastic syndromes (MDS). However, a significant proportion of MDS patients has no such mutations. Whole-genome sequencing (WGS) empowers the discovery of novel prognostic genetic alterations. We conducted WGS on pre-alloHCT whole-blood samples from 494 MDS patients. To nominate genomic candidates and subgroups that are associated with overall survival, we ran genome-wide association tests via gene-based, sliding window and cluster-based multivariate proportional hazard models. We used a random survival forest (RSF) model with build-in cross-validation to develop a prognostic model from identified genomic candidates and subgroups, patient-, disease- and HCT-related clinical factors. Twelve novel regions and three molecular signatures were identified with significant associations to overall survival. Mutations in two novel genes, *CHD1* and *DDX11*, demonstrated a negative impact on survival in AML/MDS and lymphoid cancer data from the Cancer Genome Atlas (TCGA). From unsupervised clustering of recurrent genomic alterations, genomic subgroup with *TP53*/del5q is characterized with the significant association to inferior overall survival and replicated by an independent dataset. From supervised clustering of all genomic variants, more molecular signatures related to myeloid malignancies are characterized from supervised clustering, including Fc-receptor *FCGRs,* catenin complex *CDHs* and B-cell receptor regulators *MTUS2*/*RFTN1*. The RSF model with genomic candidates and subgroups, and clinical variables achieved superior performance compared to models that included only clinical variables.

**Supplementary Information:**

The online version contains supplementary material available at 10.1186/s13045-023-01431-7.

## To the editor

Myelodysplastic syndromes represent a heterogeneous group of myeloid malignancies with increased risk of progression to acute myeloid leukemia (AML). Recurrent mutations in *TP53*, *RAS*, *JAK2*, *TET2*, *EZH2*, *ETV6*, *RUNX1*, *DNMT3A* and *ASXL1* mutations are associated with poor survival after alloHCT, the only curative therapy for MDS (Additional file 1: Table S1) [1–6]. To overcome the complexity of genomic alterations in MDS, several analytic approaches have recently been developed with clustering-based or prior knowledge network-based models [7]. However, no previous study attempted to characterize mutational signatures with clinical relevance to post-transplant outcome at a whole-genome level.

Here using multivariable survival models with selected clinical variables and artificial intelligence-based modeling approaches on WGS data (Additional file 1: Table S2), we investigated both individual-level and subgroup-level impact of genomic mutations on post-alloHCT survival of MDS patients from CIBMTR registration. (The details of CIBMTR data and sample source, outcome association, clustering and modeling can be found in the supplementary methods section.)

## Novel somatic mutations are associated with post-transplant overall survival

**Fig. 1:**
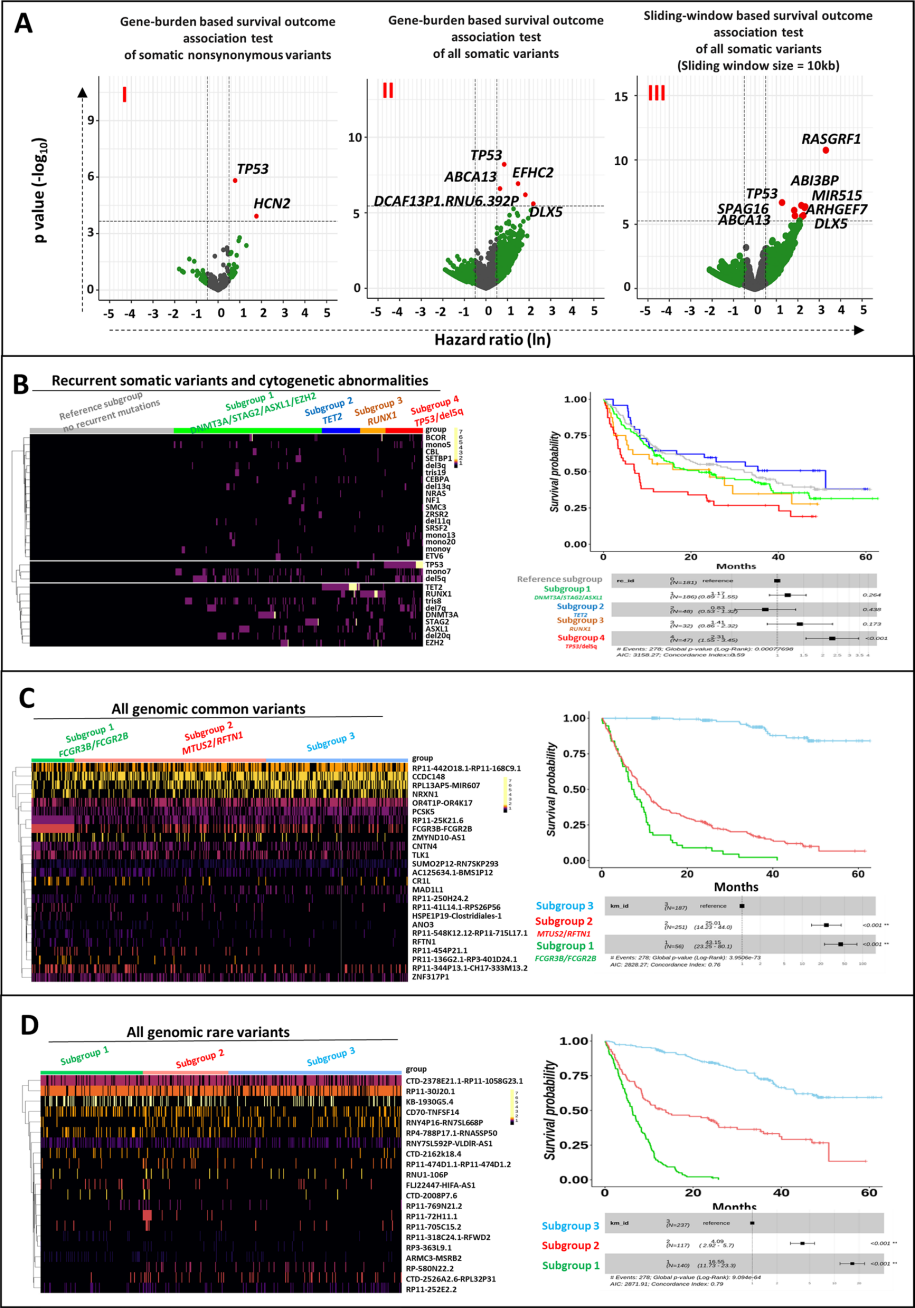
Genomic variants significantly associated with OS among the whole MDS cohort. **A** Volcano plot for genome-wide scanning of overall survival outcome association, respectively, for gene-based test of all nonsynonymous somatic coding variants (left), gene-based test of all somatic variants (middle), sliding window test of all somatic variants (right). **B** Heatmap of MDS genomic subgroups, respectively, using recurrent genomic alterations and K-means clustering. The survival curves associations of MDS genomic subgroups, respectively, using recurrent somatic mutations and cytogenetic abnormalities. **C** and **D** Heatmap and survival curve plots of MDS genomic subgroups using supervised clustering, respectively, for all genomic common variants and rare variants

In genome-wide scanning of somatic nonsynonymous coding variants in the whole cohort (*n* = 494, Additional file 1: Table S3), variants in *HCN2* and *TP53* genes were associated with inferior OS (Fig. 1A I, Additional file 1: Tables S6–S7). In sensitivity analysis among the patients who were without recurrent mutations (*TP53*, *RAS*, *JAK2*, *TET2*, *EZH2*, *ETV6*, *RUNX1*, *DNMT3A* and *ASXL1*) (*n* = 301) (see Additional file 1: Table S4), nonsynonymous somatic variants in the *DDX11* gene were associated with inferior OS (Additional file 1: Fig. S4A I, Additional file 1: Tables S6–S7).

In gene-based and sliding window-based analyses of all somatic variants, we identified 11 additional regions (*TP53, EFHC2, ABCA13, DCAF13P1.RNU6.392P, DLX5, RASGRF1, SLIT3, ABI3BP, MIR7515, SPAG16 and ARHGEF7-AS*) that were associated with inferior OS (Fig. 1A II-III, Additional file 1: Tables S6–S7). In sensitivity analysis among the 301 patients, we identified 7 novel genomic regions (*CHD1, RN7SKP174.EI24P4, EIF2B2, RP11-666E17.1-Metazoa_SRP, RP11-950C14.3, SEC14L3 and bP-2171C21.3*) that were associated with inferior OS (Additional file 1: Fig. S4A II-III, Additional file 1: Tables S6–S7). The set of genes was significantly enriched in the *TP53*-centered pathway network (Gene set enrichment analyses *p* value: 0.0042, Additional file 1: Fig. S5). In addition, a collection of analyses based on external annotations support the clinical impact of most variants and genes that were associated with inferior OS in our cohort (Additional file 1: Figs. S6-S7, Additional file 1: Tables S11-S15).

The impact of novel mutations in DNA repair pathway genes—DDX11 and CHD1—on OS associations was supported among patients with hematologic malignancies whose survival is reported to the TCGA database (Additional file 1: Figs. S8-S9). In multivariate analyses in our cohort, DDX11 and CHD1 were shown to impact OS through an increased risk of both relapse and TRM (Additional file 1: Figs. S10-S11). DDX11 dysfunctions were linked to myeloid neoplasms via promoting cell proliferation [8], while CHD1 plays a critical role in gating transcription landscape of hematopoietic stem and progenitor cells (HSPCs) [9]. A recent study suggested that mutant CHD1 might lead to resistance to standard therapies due to attenuated DNA damage responses in AML/MDS patients [10]. We found that 3 CHD1 noncoding mutations map to known enhancer loci or transcription binding sites, revealing their regulatory functionalities.

## The association of genomic subgroups with post-transplant overall survival

Unsupervised clustering analyses of recurrent somatic variants and cytogenetic abnormalities identified four distinct clusters. The molecular signatures in these four clusters were found to be *DNMT3A*, *STAG2* and *ASXL1* (subgroup 1), *TET2* (subgroup 2), *RUNX1* (subgroup 3), and *TP53* and del5q (subgroup 4), respectively (Fig. 1B). Compared to the reference subgroup, Cox multivariate models revealed that genomic clusters with *TP53* mutations and the del5q (*p* < 0.001**) have strong associations with post-transplant overall survival outcome in both whole cohort and independent replication cohort (Fig. 1B, Additional file 1: Fig. S14, Additional file 1: Table S8). To be noted, although genomic subgroup 1 with DNMT3A, STAG2 and ASXL1 mutations and subgroup 3 with RUNX1 mutations showed adverse survival risk stratifications (Fig. 1B), the results were not statistically significant in our MDS cohort and might be of interest in the future studies.

Supervised clustering analyses of all genomic common variants identified three distinct clusters. To ensure the robustness of genomic clustering, the consistent profiles of survival outcome associations are confirmed in different k-fold cross-validations of supervised clustering (Additional file 1: Fig. S12). Additionally, competing risk regression and Cox proportional regression analyses of the association of genomic signatures from clustering were conducted and confirmed the associations with relapse, OS and DFS (Additional file 1: Fig. S13). The main molecular signatures in these three clusters are Fc-receptor gene *FCGR3B* and *FCGR2B* (subgroup 1) and microtubule binding protein *MTUS2* and *RFTN1* (subgroup 2) (Fig. 1C, Additional file 1: Table S16). Compared to the subgroup 3, Cox multivariate models revealed that genomic clusters with *FCGR3B*/ or *MTUS2*/*RFTN1 mutations* have strong associations with post-transplant overall survival outcome (Fig. 1C, Additional file 1: Table S16). From supervised clustering analyses of all genomic rare variants, the main molecular signatures were mostly found to be from long noncoding RNA (LncRNA) (Fig. 1D, Additional file 1: Table S16).

## Genomic signature-based prognostic models on post-transplant overall survival

The prediction performance of RSF models that incorporated genomic signatures from supervised clustering analyses was excellent with C-index 0.83 alone and 0.84 if combined with genomic association candidates (Table 1), as well as other survival models (Additional file 1: Table S9). To assess the calibration and clinical usefulness of the clinical prediction model, the Brier score for all RSF models has been computed and ranged from 0.07 to 0.22, indicating that RSF models performed well on both discrimination and calibration (Additional file 1: Table S10). In particular, the models with genomic components have very low Brier scores below 0.10, supporting their clinical usefulness on post-HCT overall survival prognosis of MDS patients. Comparable C-index were shown when the RSF models stratified with different conditioning regimens, as well as other outcomes DFS, relapse and TRM (Table 1). Indeed, feature importance evaluations supported that genomic subgroup from supervised clustering was the most important features in the RSF model, and even present greater importance than mutational number uncovered from genomic association candidates (Additional file 1: Fig. S16). The results suggested that molecular signatures from all genomic mutations could potentially provide more prognostic information than somatic recurrent mutations.

**Table 1:** Comparison of the concordance index among RSF models

Survival model/concordance (95%CI)	OS	DFS	Relapse	TRM
Base model	0.49 (0.44–0.52)	0.48 (0.43–0.50)	0.45 (0.39–0.50)	0.45 (0.37–0.50)
Clinical model	0.54 (0.53–0.60)	0.55 (0.51–0.58)	0.55 (0.50–0.60)	0.54 (0.48–0.59)
Genomic model	0.83 (0.81–0.85)	0.75 (0.73–0.77)	0.80 (0.77–0.82)	0.80 (0.78–0.83)
Full model	0.84 (0.83–0.86)	0.78 (0.76–0.81)	0.73 (0.70–0.77)	0.85 (0.82–0.87)
Full model (regimen = myeloablative)	0.83 (0.80–0.86)	0.79 (0.75–0.83)	0.75 (0.69–0.81)	0.85 (0.80–0.90)
Full model (regimen = reduced intensity)	0.83 (0.80–0.85)	0.79 (0.76–0.81)	0.77 (0.74–0.80)	0.84 (0.82–0.87)

Base model: IPSS-R

Clinical model: Base model + mdstype + HMA + CHEMO

Genomic model: genomic association candidates + genomic clustering subgroups

Full model: Clinical model + Genomic model

Even though our models incorporated internal validation, our results require further validation in another independent dataset. Furthermore, the WGS data represent the genomic landscape at the time of alloHCT and lack the comparison to the landscape at diagnosis. Lastly, 100% of our subjects were white, and therefore, these results are not representative of racially/ethnically diverse populations.

Based on the classical IPSS-R model, a recent study developed an innovative personalized prognostic model—IPSS-Molecular (IPSS-M) model, with improved discrimination across all key endpoints [11]. The IPSS-M model integrates clinical, cytogenetic and molecular information. However, the recurrent somatic mutations in IPSS-M model were based on targeted gene sequencing with deeper depth > 200×, which are unavailable in our MDS cohort with 60× depth. Although our WGS-based study may miss extremely small subclones in somatic genomics of MDS patients, it does empower the discovery of novel genetic biomarkers and could potentially provide additional prognostic stratification information to the IPSS-M model. Further investigations would be of great clinical value toward developing the genomic model combined with WGS -based novel genetic biomarkers and IPSS-M.

In summary, our analyses identified novel prognostic factors of post-transplant survival that were centered by *TP53* pathway network, and novel molecular signatures involved in multiple immune regulatory pathways. Our RSF models have demonstrated the substantial prognostic contribution of these novel genomic candidates for alloHCT outcomes in MDS. This study supports the key role of WGS in elucidating the prognostic impact of genomic alterations in a disease known to be quite molecularly heterogeneous, such as MDS. These genomic alterations would not be identified with targeted gene panels sequencing alone. With the continuous reduction in costs of WGS, this technology could be an essential tool in future research and perhaps in clinical care, at an affordable rate [12].

## Supplementary Information

**Additional file 1:** Supplementary Methods and Results.

## Data Availability

The source codes and documentations of supervised clustering survival workflow can be found here: https://github.com/tzhang-nmdp/supervised-clustering-survival. CIBMTR supports accessibility of research in accord with the National Institutes of Health (NIH) Data Sharing Policy and the National Cancer Institute (NCI) Cancer Moonshot Public Access and Data Sharing Policy. The CIBMTR only releases de-identified datasets that comply with all relevant global regulations regarding privacy and confidentiality.

## Abbreviations

WGS: Whole-genome sequencing
MDS: Myelodysplastic syndromes
alloHCT: Allogeneic hematopoietic cell transplant
RSF: Random survival forest machine learning model
OS: Overall survival
DFS: Disease-free survival
TRM: Transplant-related mortality
CI: Confident interval
AML: Acute myeloid leukemia
c-index: Concordance index
CIBMTR: Center for International Blood and Marrow Transplant Research
TCGA: The Cancer Genome Atlas (TCGA) database
IPSS-R: Revised International Prognostic Scoring System
lncRNAs: Long noncoding RNAs
