# Supplementary material for: Whole-genome sequencing identifies novel predictors for hematopoietic cell transplant outcomes for patients with myelodysplastic syndrome: a CIBMTR study
Source: J Hematol Oncol. 2023 Apr 11;16:37. doi: 10.1186/s13045-023-01431-7 (PMC10088148; doi:10.1186/s13045-023-01431-7)
Supplement: Supplementary file 1 — Additional file 1: Supplementary Methods and Results. [file 13045_2023_1431_MOESM1_ESM.docx]

Supplementary Appendix

This appendix has been provided by the authors to give readers additional information about their work.

Supplement to: Zhang T; Auer P; Spellman S; Bolon Y, Saber W, et al. Whole Genome Sequencing Identifies Novel Predictors for Hematopoietic Cell Transplant Outcomes for Patients with Myelodysplastic Syndrome: A CIBMTR Study.

**Table of Contents**

**Supplementary methods**

Data Source .................................................................................................................................................5

Patient Sample Preparation .........................................................................................................................5

Whole Genome Sequencing Procedure .......................................................................................................5

Somatic Variant Process ..............................................................................................................................6

Outcome Definitions ……………………........................................................................................................7

Supervised clustering algorithm ...................................................................................................................8

Survival Outcome Association Analyses ....................................................................................................10

Functional Annotation Analyses of Genomic Association Candidates ........................................................13

**Supplementary Figures**

Figure S1 -- The somatic genomic profile of MDS recurrently mutated gene coding variants………............15

Figure S2 -- The mutational signature profiles of somatic variants in our MDS cohort...............................16

Figure S3 -- The qqplot for overall survival outcome association analyses……………................................16

Figure S4 -- Genomic variants significantly associated with OS among the subset cohort …………..…....17

Figure S5 -- The overrepresentation of top somatic prognostic candidate genes in TP53-centered pathways.....................................................................................................................................................17

Figure S6 -- The expression levels of somatic prognostic candidate genes in GTEx whole blood RNAseq data…………...............................................................................................................................................18

Figure S7 -- The expression dysregulations of *ABCA13*, *DDX11*, *CHD1, EFHC2, EIF2B2* and *HCN2* between healthy and MDS CD34+ HSCs in GEO data……………………………………..............................18

Figure S8 -- The survival correlations of *ABCA13*, *DDX11*, *CHD1, EFHC2, EIF2B2* and *HCN2* in TCGA dataset........................................................................................................................................................19

Figure S9 – The variant distribution summary of *DDX11* and *CHD1* in our MDS cohort and TCGA databset......................................................................................................................................................19

Figure S10 -- Survival outcome association summary of *DDX11* and *CHD1* in our MDS cohort…….........20

Figure S11 -- Competing Risks Regression analyses of *DDX11* and *CHD1* in our MDS cohort…………….21

Figure S12 -- Survival curve plots for the robustness of supervised clustering ………………………..……..22

Figure S13 -- Survival curve plots of different survival outcomes for supervised clustering ………………..22

Figure S14 -- Replication of genomic subgroups based on somatic recurrent mutations in an independent MDS cohort………………………………………………………………………………………………………….23

Figure S15 -- The molecular signatures FCGR3B/FCGR2B in genomic subgroup are mutated in the region of known gene enhancer and transcription factor binding site……………………………..……………..…...24 Figure S16 -- Summary of genomic clustering subgroups and genomic association candidates in RandomForest survival (RSF) models ............……………………………………………………………..…...25

**Supplementary table**

Table S1 -- Known prognostic genes from previous clinical studies............................................................26

Table S2 -- Clinical variable selection for overall survival.............................................................................26

Table S3 -- Demographic characteristics of whole cohort…………………………………….…………….....26

Table S4 -- Demographic characteristics of subset cohort…………………………………….…………….....28

Table S5 -- The hematologic malignancies AML/MDS and lymphoma cohort in TCGA database…….......29

Table S6 – Summary of candidate associations with overall survival outcome in multivariate coxph model………………………………………………………………………………………………………………...29

Table S7 -- Permutation validation of candidate associations with overall survival outcome in univariate coxph model...............................................................................................................................................30

Table S8 -- The table of genomic subgroups in discovery and replication MDS patient cohort by K-means clustering based on recurrent somatic mutations and cytogenetic abnormalities………………………….....31

Table S9 -- The prognostic effects of genomic variables on survival outcome in different models ………..31

Table S10 -- The model performance calibration of RSF survival models by Brier scores .........................31

Table S11 -- Gene annotations for top candidates of overall survival outcome from different association analyses ……………………………………………………………………...…………………………….............32

Table S12 -- GTEx annotations of pre-computed whole blood eQTL analyses for candidate genes with significant associations......………………………… ……………………………………………………….........33

Table S13 -- Variant deleteriousness annotations for candidate genes......................................................33

Table S14 -- Top candidates of overall survival outcome from different association analyses……….…….37

Table S15 -- Patient level and variant level data for candidate genes in our MDS cohort…………….……...45

Table S16 -- Gene annotation table for Top genes in supervised clustering analysis of common variants………………………………………………………………………………………………………………50

Table S17 -- Gene annotation table for Top genes in supervised clustering analysis of rare variants…………………………………………………………………………………………...………………….55

References……………………………………………………………………...………...……59

**Supplementary methods**

**Data Source**

The CIBMTR is a research affiliation between the National Marrow Donor Program/Be The Match and the Medical College of Wisconsin. It facilitates critical research through medical, scientific, and statistical expertise; a network of more than 330 participating centers; a database with clinical data on 575,000 patients; and a biospecimen repository.^1^ The study was approved by the Institutional Review.

**Patient Sample Preparation**

Consecutive patients with MDS undergoing alloHCT from 2014-2018 and reported to the CIBMTR with banked pre-alloHCT whole blood samples were included in the study. All samples were collected in ACD-A tubes before the administration of the preparative conditioning regimen prior to transplantation and shipped overnight at ambient temperature to the CIBMTR Research Repository, aliquoted on the day of receipt and stored frozen at -80C or in N2(l). DNAs were extracted using either the Qiagen Puregene Method or DNA Blood Kit on the Perkin Elmer Chemagic 360. Qiagen Puregene kit was used for DNA extraction with a DNA yield of 100-500ng/ul DNA per sample.

**Whole Genome Sequencing Procedure**

Whole genome sequencing was conducted by Broad Institute using a modified version of the Illumina TruSeq PCR-Free LT Library Prep method (Illumina). Briefly, 3-4ug gDNA was fragmented via Covaris acoustic shearing to a size of 900-1000bp. Sheared samples were then dual size selected using AMPure XP beads (Beckman Coulter) to first select for larger fragments, and secondly to select out small fragments. End repair followed the standard Illumina protocol. Following End repair, a second dual size selection, identical to the first, was conducted to further select for the correct fragment size. Adenylation and Adapter ligation followed the Illumina standard protocol. Following adapter ligation, a final set of two AMPure washes was completed to purify final whole genome libraries. Final qualification and quantification of libraries was conducted on a Fragment Analyzer (Advanced Analytical) and via qPCR using Universal qPCR MasterMix (Kapa Biosystems) on a CFX384 quantitative thermalcycler (Bio-Rad). Libraries were pooled at 3uM and sequenced via Illumina MiSeq to QC for cluster density yield and index distribution. Samples were then normalized to appropriate concentration to load on the Illumina Novaseq 6000 (Illumina) using 2x150 paired-end sequencing on an S4 flowcell. Pool size was 12 samples to achieve the required 60x depth minimum for each sample. The raw bam files were generated with hg38 references.

**Somatic Variant Process**

For somatic calling of single nucleotide variants and small insert-deletions (indels) <250bp, we process all samples with OCTOPUS (github.com/luntergroup/octopus/tree/master).^2^ OCTOPUS implements a Bayesian genotyping model and variant calling algorithm. First it generates allele and haplotype information from sequencing reads. Then, based on different model assumptions, it computes genotype/haplotype posteriors and calls somatic variants. Additional filtering steps were applied on somatic variants based on multiple databases: 1. Remove variants with Gnomad allele frequency above 10E-06; 2. Remove noncoding variants in low complex and repetitive regions or with no functional indications from ANNOVAR annotations, ^3^and CADD database conservative score under 15,^4^ and absence in HGMD^5^ and COSMIC database.^6^ Somatic variants with VAF>=50% were checked CNV by CNVkit.^7^ For the clonal architecture analyses, we used CNVkit to identify copy-number alterations across the genome and the SciClone R package to analyze the clonality.^8^ The mutational signatures were extracted and characterized using SigProfiler workflow.^9^ Each of the extracted process was assigned to one specific mutational signature included in the updated COSMIC (release v93, March 2021).^10^

The WGS provided mean genome coverage of 77X (59~146X), a mean somatic variant count per sample of 864 (379~2743). To assess the clonality of somatic mutations in MDS, VAF and CNA information were integrated into a variational Bayesian mixture model to infer the clone boundary and clone number. In our MDS cohort, a mean clone number of 1.5 (1~3) with mean VAF 19.6% were detected for recurrent somatic mutations, while TP53, TET2, RUNX1, DNMT3A and ASXL1 are the most frequently mutated genes (Fig. S1).

As compared to target gene sequencing, especially at extremely low VAF, WGS have lower sensitivity for the detection of somatic single-nucleotide variants and small insertions or deletions^11,12^. Sequencing depth settings and tumor clonal heterogeneity are the major factors impacting the detection thresholds of our analyses, which have been extensively discussed in previous studies^13-15^. Initially, the unfiltered recurrent somatic mutations were detected in 70% of patients in our cohort, which is slightly lower than previous reports^16-18^. Given the fact that DNA samples were collected from MDS patients after certain treatments like HMA, Chemo, or both, the possible explanation might be that the clone size of some somatic mutations were reduced under the detection threshold of current WGS settings. To minimize germline misclassifications, additional filtering steps were applied, and ultimately recurrent somatic mutations were identified in 60% of patients in our cohort (Fig. S1A). However, for most somatic variants absent in our WGS analyses due to extremely low VAF or high frequency in normal population (which would have been filtered out, see preceding sentence), further investigations would be of great value to determine their clinical significance.

The SBS mutational signature analysis revealed that signature SBS5 (72.5%), SBS23 (24.52%) and SBS1 (2.94%). SBS5 and SBS1 (associated with deamination of 5-methylcytosine to thymine) are both clock-like mutational process that are ubiquitous among cancer types and were present in most cancer cell lines and patient-derived xenografts.15 While SBS23 exhibits strong transcriptional strand bias for C>T mutations with damage to guanine and repair by transcription-coupled nucleotide excision repair (Fig. S2).

**Outcome Definitions**

*Primary Outcome*

The primary outcome was overall survival (OS), defined as time to death. Death from any cause was considered an event. Surviving patients were censored at time of last follow-up.

*Secondary Outcomes*

Relapse was defined as time to onset of first hematologic or clinical recurrence of MDS. Death in remission was treated as a competing risk. Surviving patients in remission were censored at time of last follow-up.

Transplant related mortality (TRM) was defined as any death in the first 28 days, or time to death in remission after the first 28 days. Relapse was treated as competing risk for deaths occurring after 28 days. Surviving patients in remission were censored at time of last follow-up.

Disease-free survival (DFS) was defined as time to relapse, or death in first 28 days from any cause, or death in remission from any cause after 28 days. Surviving patients in remission were censored at time of last follow-up.

**Supervised clustering algorithm**

The supervised clustering algorithm of all genomic variants on survival outcome has been as implemented as the pseudo code below (Detailed documentations and source codes can be found here: https://github.com/tzhang-nmdp/Supervised-clustering-survival):

Input - genomic variables from samples except those from the genomic cluster *TP53*/del5q: Common variants after being pruned by standard PLINK packages, or rare variants within the same gene region are aggregated.

1. For each point in genomic variables, exclude the variables if they failed for linearly independence check and L1 regulation (optional).

2. Stratified random split of samples based the response variable (death event) into k folds for cross-validations.

3. For samples ∈ 1:k folds, compute the 1^st^ weight for each genomic variables based on its effect size : $w_{or}\left( x_{i},y \right)=\log(N_{x_{i}=0,y=0}\times N_{x_{i}=1,y=1})/{(N}_{x_{i}=0,y=0}{\times N}_{x_{i}=1,y=1})$.

4. For samples ∈ 1:k folds, compute the 2^nd^ weight for each genomic variables based on its GO semantic similarity: $w_{sem}\left( x_{i},{x_{i}}^{c} \right)=\sum_{j\in{x_{i}}^{c}}^{j} {sem}_{i,j}/\sum_{i\in n}^{i} \sum_{j\in x_{i}^{c}}^{j} {sem}_{i,j}$.

5. For samples ∈ 1:k folds, formulate the combined weight for each genomic variables with hyperparameter θ: $w=w_{or}\left( x_{i},y \right)\theta+w_{sem}\left( x_{i},{x_{i}}^{c} \right)(1-\theta)$.

6. For cluster number C ∈ 3:10 and hyperparameter θ ∈ 0:1, run the iterations of K-Means clustering.

6.1 For each iteration, compute the silhouette score for quality control of K-Means clustering (no significant variations and no negative score for individual clusters).

6.2 For each iteration, relabel the cluster id and compute pair-wised log-rank tests of the same clusters between different k-folds and hold-out samples for quality control of K-Means clustering (no significant variations of survival distributions in cross-validations).

6.3 For each iteration, compute the c-index of the sample cohort in different k-folds and hold-out samples.

7. Determine the optimal cluster number C and hyperparameter θ based on the best c-index and no significant variations of silhouette score and log-rank tests.

Summary of the genomic subgroups and association of cluster identities (based on pre-computed centroids) with overall survival is illustrated in Figure 1.

**Survival Outcome Association Analyses**

In order to test for associations between genomic mutations and survival outcomes, we selected covariates to include in the Cox proportional hazards model via forward-backward variable selection (the list of all potential covariates is given in Table 1). We first constructed a model that tested only the prognostic importance of clinical variables in our cohort for OS. The final model indicated that the following covariates were important: IPSS-R, pre-transplant treatments, and MDS type) (Table S2).

Next, we constructed a combined clinical/genomic model to test individual level associations of somatic mutations. In coding regions, we conducted gene-based tests of somatic variants by summing the number of rare alleles from somatic nonsynonymous (missense, nonsense, splice) mutations per gene and testing for association with overall survival via the Cox model.^19^ For non-coding regions, we applied a sliding window-based approach, summing the number of rare alleles from somatic mutations per window of 10kb region and testing for association with overall survival via the Cox model. The prognostic effects of genomic candidates and important clinical variables were systematically evaluated in a multivariate Coxph model (Table S6). The potential candidates were determined based on the cutoffs of both adjusted p value 0.05 and coefficient (log hazard ratio) 0.5. To reduce the overfitting effects from coxph model, only somatic collapsed groups with equal or more than 5 hits will be considered. Quantile-quantile plots showed the distribution of observed and expected P values for each test were delineated with reasonable positive candidates (Fig. S3). For each candidate, the association was further validated by permutation method, coefficient, hazard ratio (HR), standard deviation of the coefficient, 95% confidence intervals (CIs), and adjust p value with Bonferroni correction were summarized (Table S7).

Sixty percent of subjects (N=301) in our cohort were without recurrent mutations across a wide range of recurrently mutated genes (*TP53*, *RAS*, *JAK2*, *TET2*, *EZH2*, *ETV6*, *RUNX1*, *DNMT3A* and *ASXL1*) in myeloid malignancies (Table S1, S3). For overall survival association tests, we conducted a sensitivity analysis to determine whether we could identify any novel genomic prognostic markers among this subset.

For all genomic candidates that were associated with inferior post HCT survival, we externally replicated our findings among patients with hematologic malignancies whose outcomes are reported in the Cancer Genome Atlas (TCGA) database (Table S4).^20^

To evaluate the risk stratification effects of complex molecular signatures from a whole genome level, we characterized such genomic subgroup entities by unsupervised and supervised clustering analysis. For recurrent somatic mutations and cytogenetic abnormalities, we conducted unsupervised K-means clustering to define subgroups based on absence/presence of recurrent genomic events (rare genomic events were excluded with occurrences <5). We used the current cohort (N=494) as a discovery dataset, while MDS patients included in a prior publication were used as a replication dataset after applying additional matching factors, such as ethnicity, age and graft source (N=1514).^21^ For all genomic mutations, we conducted supervised clustering method to characterize the molecular signatures of common variants and rare variants separately. This supervised clustering algorithm is based on the integrated weights of both GO semantic similarity and statistical association effect. The algorithm mines the mutational clusters with strong survival outcome stratifications. Multiple survival association-based metrics and clustering distance-based metrics were evaluated to determine the optimal results. More details of association, clustering and additional analyses can be found in Supplementary Appendix.

To further assess the impact of genomic alterations on OS, we applied a random survival forest (RSF) model to the full cohort. Because the overall mutation number has been reported as an important negative prognostic factor for overall survival in MDS patients,^22^ we included the overall mutational number into our RSF model for predicting OS. Specifically, we built three different models for comparison: a base model (with IPSS-R only), a clinical model (with IPSS-R, Pre-HCT treatments, and MDS-type) and a full model (with IPSS-R, Pre-HCT treatments, MDS-type, mutation number, mutations from significant genes/regions and subgroups from clustering from the discovery analysis). The RSF algorithm has multiple advantages. It accounts for the interactions among explanatory variables, flexible to nonlinearity between explanatory and response variables, implemented with a repeated bootstrap procedure that enhances robustness and reproducibility, and has built-in imputation function for missingness. Finally, we constructed 95% confidence intervals for the c-indices via a non-parametric bootstrap procedure (number of bootstraps=1000). A set of additional survival models were employed to assess the reproducibility the of c-indices from the RSF model.

**Functional Annotation Analyses of Genomic Association Candidates**

To explore the biological relevance of identified mutations, we performed functional annotation using the Ensembl Variant Effect Predictor (VEP).^23^ The VEP incorporates an extensive list of prediction algorithms to evaluate the potential deleteriousness of variants in both coding and non-coding regions of the genome. The algorithms used include but are not limited to SIFT, PolyPhen-2, ClinVar, the Combined Annotation Dependent Depletion (CADD), the Rare Exome Variant Ensemble Learner (REVEL), MetaLR, MTR, MutationAssessor, MutationTaster, FATHMM, and FATHMMMKL.^5,24-32^

To explore the potential functional mechanisms of candidate genes, we conducted gene set enrichment analyses on GO and KEGG pathways using STRING.^33^ To prioritize the functional importance of our top somatic prognostic candidates, a serial of meta analyses were conducted based on GTEx, GEO, and TCGA databases. The Genotype-Tissue Expression (GTEx) Project was supported by the Common Fund of the Office of the Director of the National Institutes of Health, and by NCI, NHGRI, NHLBI, NIDA, NIMH, and NINDS. Finally, we investigated *TP53* related genomic contexts on MDS post-transplant survival outcomes (cytogenetic abnormalities, and *TP53* mutation VAF, *TP53* variant type, and # of mutations in *TP53*) using Kaplan-Meier curves and log-rank tests.

**Supplementary Figures**

**
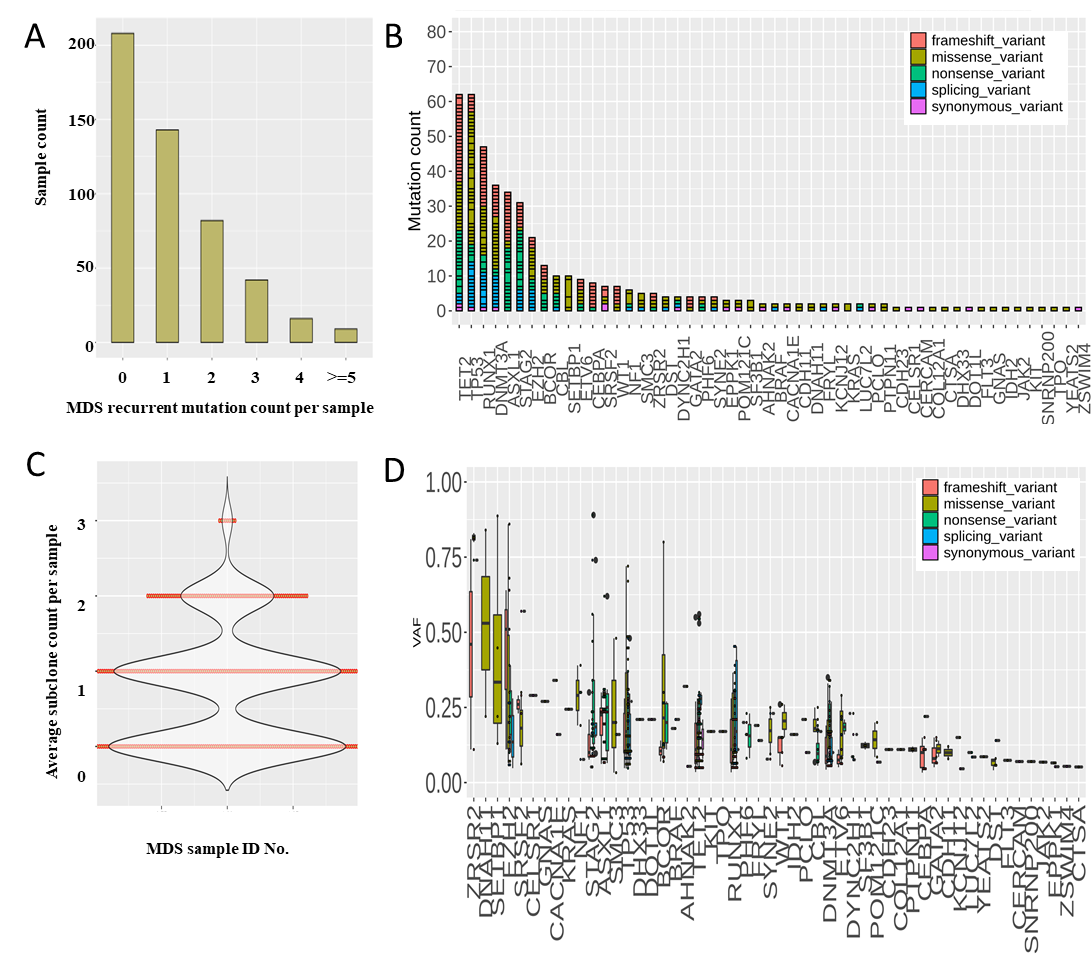
**

Supplemental Figure 1. The somatic genomic profile of MDS recurrently mutated gene coding variants.

A. The number of somatic mutations per patient in MDS recurrently mutated genes;

B. The Mutation spectrum in MDS recurrently mutated genes, x axis ordered by total recurrent mutation counts;

C. The number of somatic clones per patient in MDS recurrently mutated genes;

D. The variant allele frequency (VAF) distribution in in MDS recurrently mutated genes.


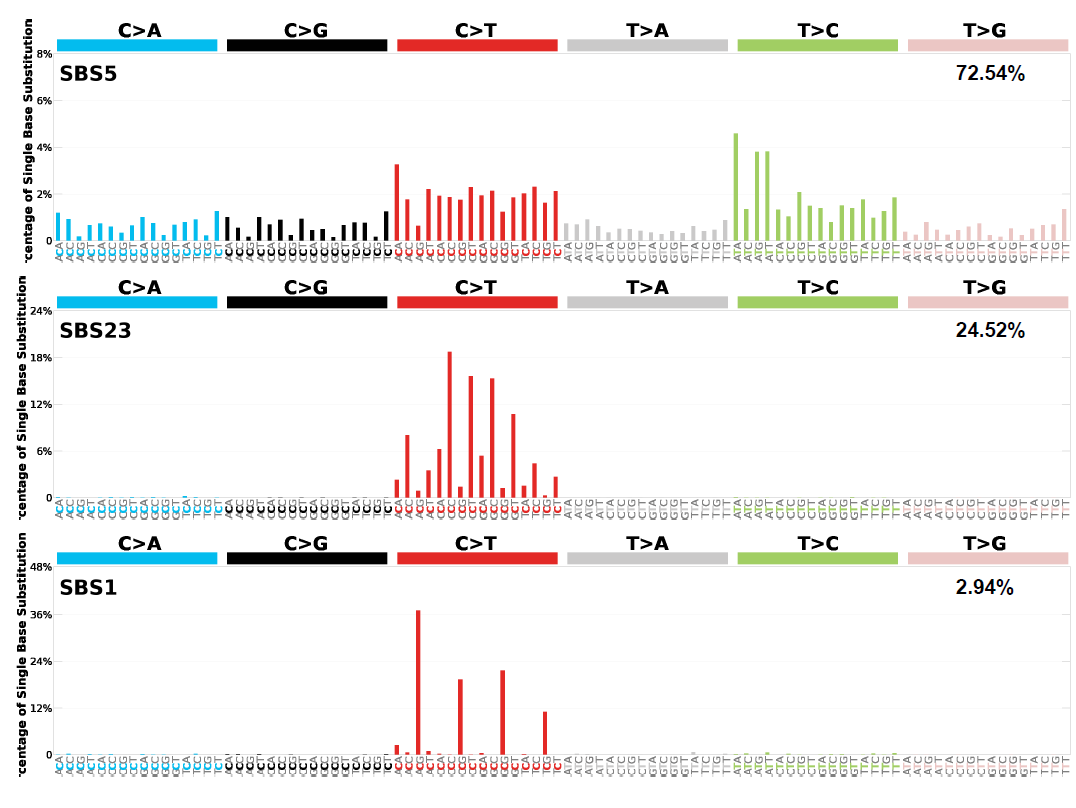


Supplemental Figure 2. The mutational signature profiles of somatic variants in our MDS cohort.


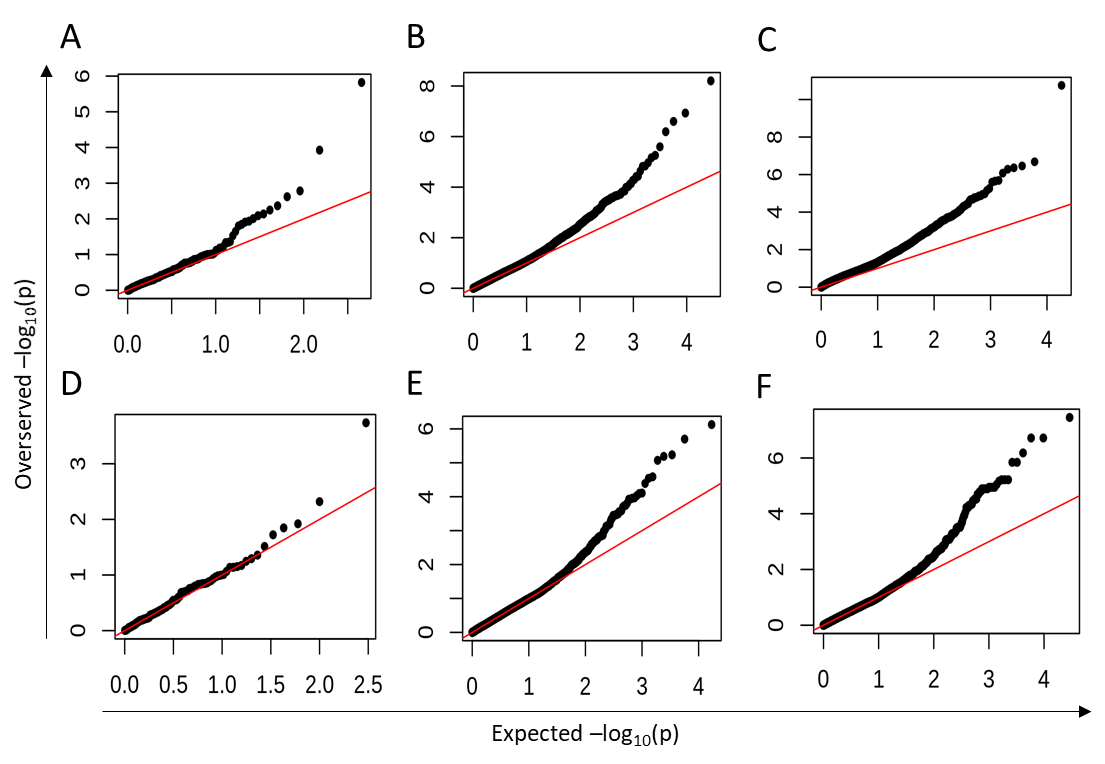


Supplemental Figure 3. The qqplot for overall survival outcome association analyses.

A. Survival outcome qqplot plot for gene burden test of all somatic coding variants in whole cohort;

B. Survival outcome qqplot for gene burden test of all somatic variants in whole cohort;

C. Survival outcome qqplot for sliding window test of all somatic variants in whole cohort;

D. Survival outcome qqplot for gene burden test of all somatic coding variants in subset cohort;

E. Survival outcome qqplot for gene burden test of all somatic variants in subset cohort;

F. Survival outcome qqplot for sliding window test of all somatic variants in subset cohort.


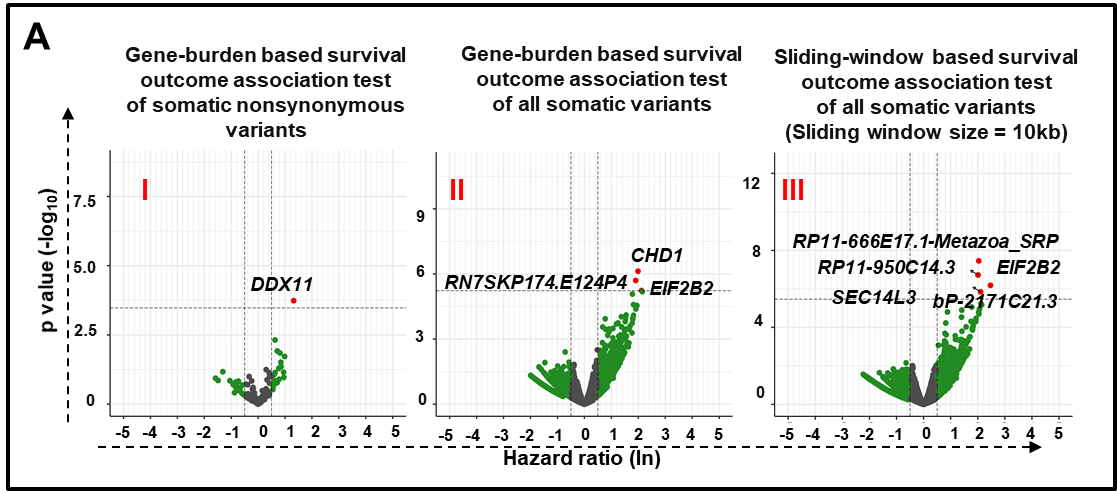


Supplemental Figure 4. Genomic variants significantly associated with OS among the subset cohort patients who were without recurrent mutations (TP53, RAS, JAK2, TET2, EZH2, ETV6, RUNX1, DNMT3A and ASXL1) . (A) The volcano plot for genome wide scanning of overall survival outcome association respectively for gene-based test of all nonsynonymous somatic coding variants (left), gene-based test of all somatic variants (middle), sliding window test of all somatic variants (right).


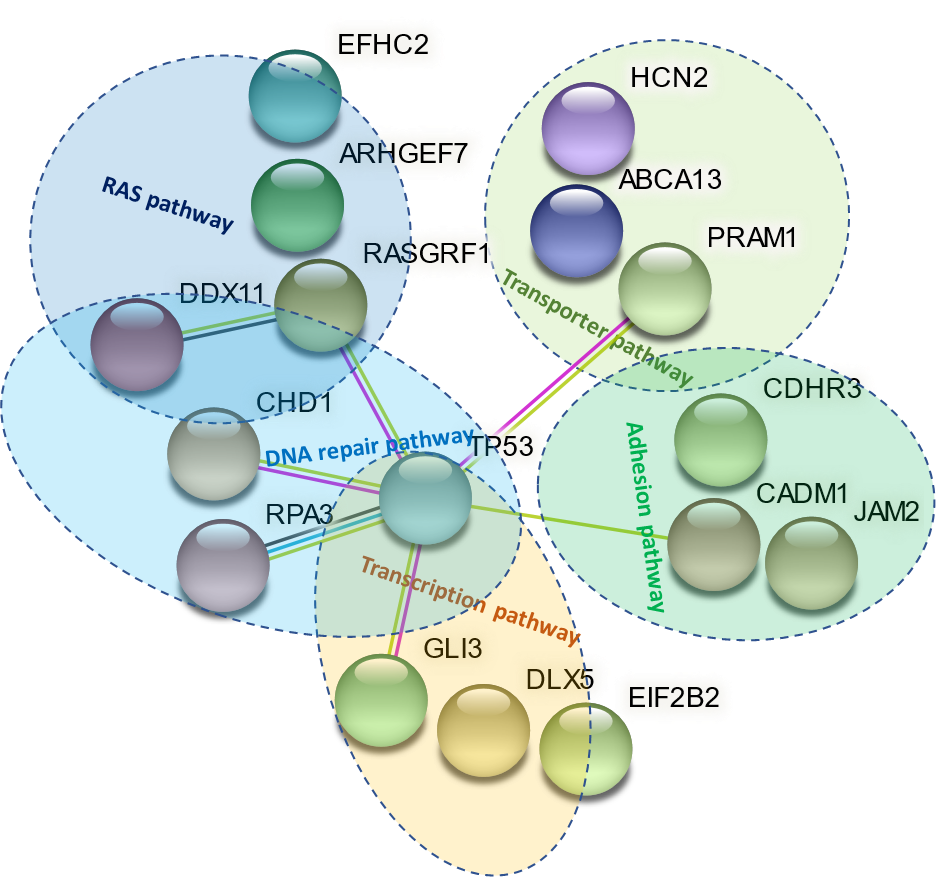


Supplemental Figure 5. The overrepresentation of top somatic prognostic candidate genes in *TP53*-centered pathways.


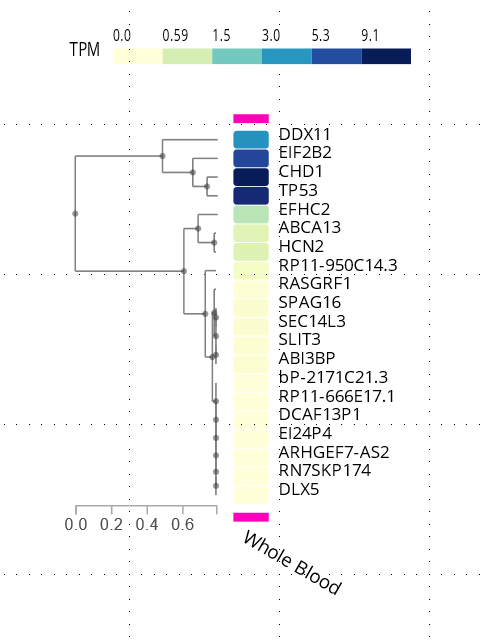


Supplemental Figure 6. The expression levels of top somatic prognostic candidates in GTEx whole blood RNAseq data.


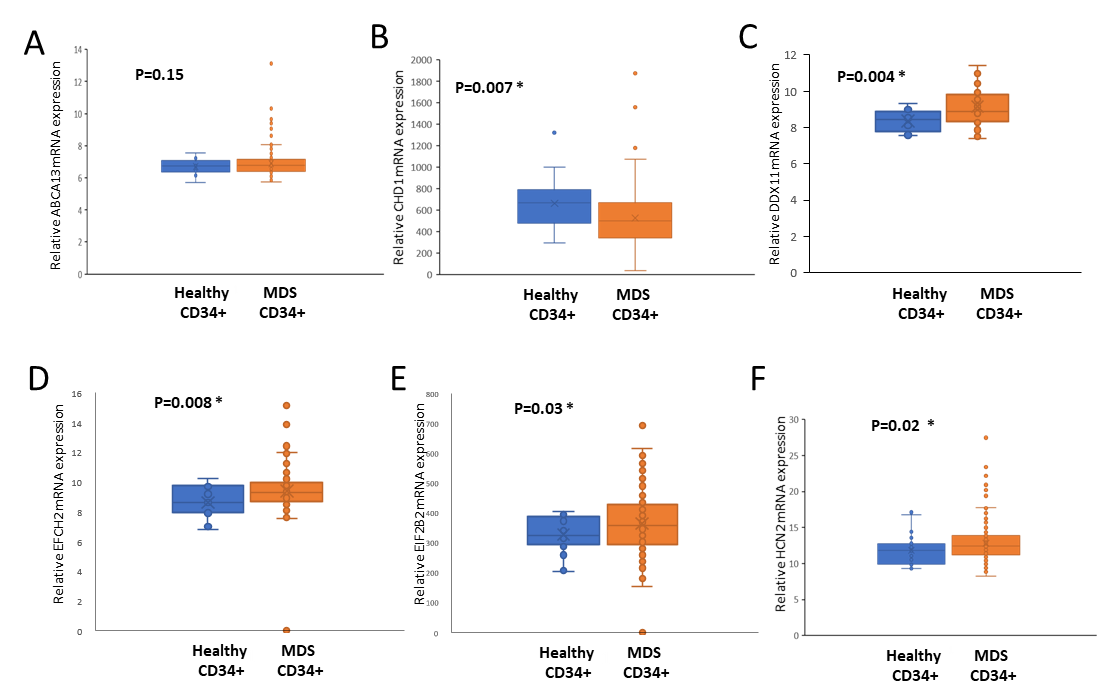


Supplemental Figure 7. The expression dysregulations of *ABCA13*(A), *CHD1*(B), *DDX11*(C), EFHC2(D), EIF2B2(E) and *HCN2* (F) between healthy and MDS CD34+ HSCs in GEO data.


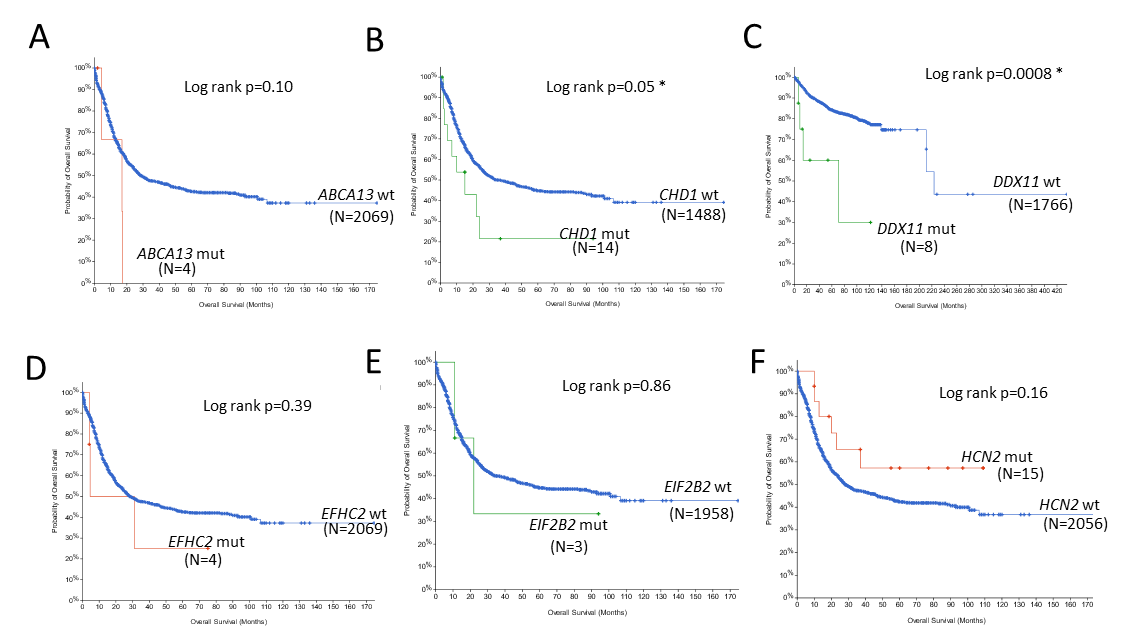


Supplemental Figure 8. The survival curves of *ABCA13*(A), *CHD1* (B), *DDX11(C), EFHC2*(D), EIF2B2(E) and *HCN2* (F) in TCGA AML/MDS datasets (Lymphoma dataset for DDX11 only).


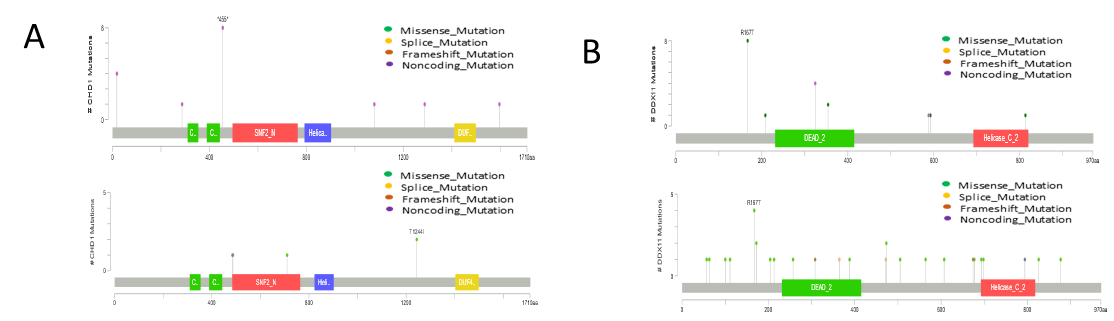


Supplemental Figure 9. The lollipop plots for non-recurrent MDS gene candidate CHD1 (A) and DDX11 (B), respectively in our MDS cohort (upper) and TGCA data (lower).


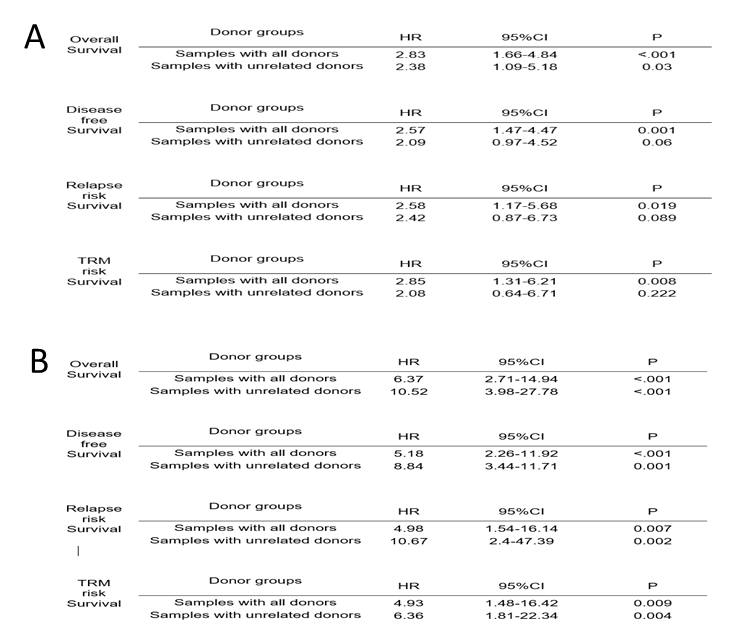


Supplemental Figure 10. Survival outcome association summary of novel biomarker candidates *DDX11*(A), and *CHD1*(B) in our MDS cohort.


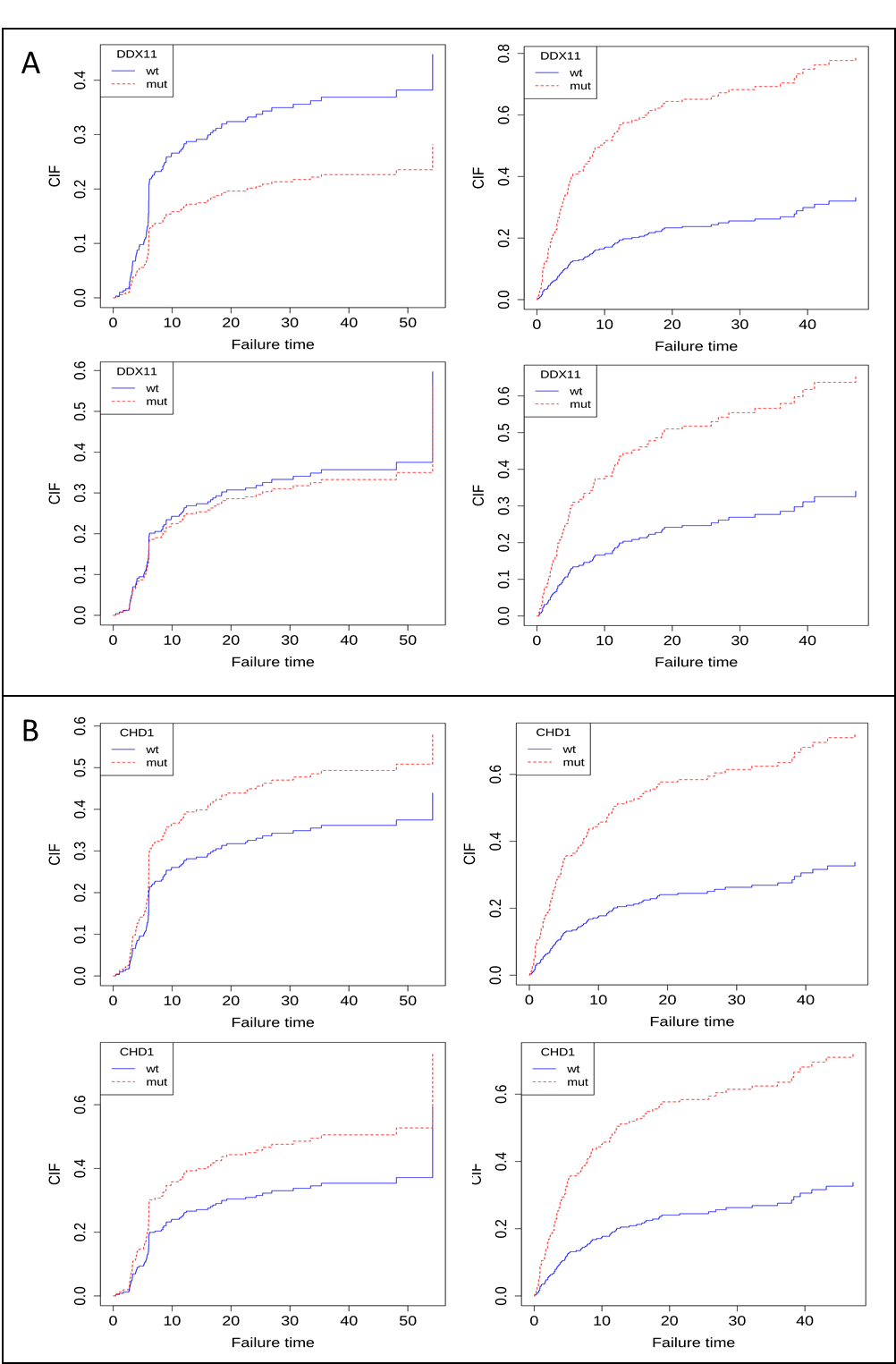


Supplemental Figure 11. Competing Risks Regression analyses in our MDS cohort respectively for *DDX11* (A) and *CHD1* (B): Relapse as primary risk in all MDS patients (upper left) and MDS patients with unrelated donor only (lower left), and TRM as primary risk and in all MDS patients (upper right) and MDS patients with unrelated donor only (lower right). *DDX11* mutations show significant associations with TRM risk in both all MDS patients (p value: <0.001) and MDS patients with unrelated donor only (p value: 0.05).


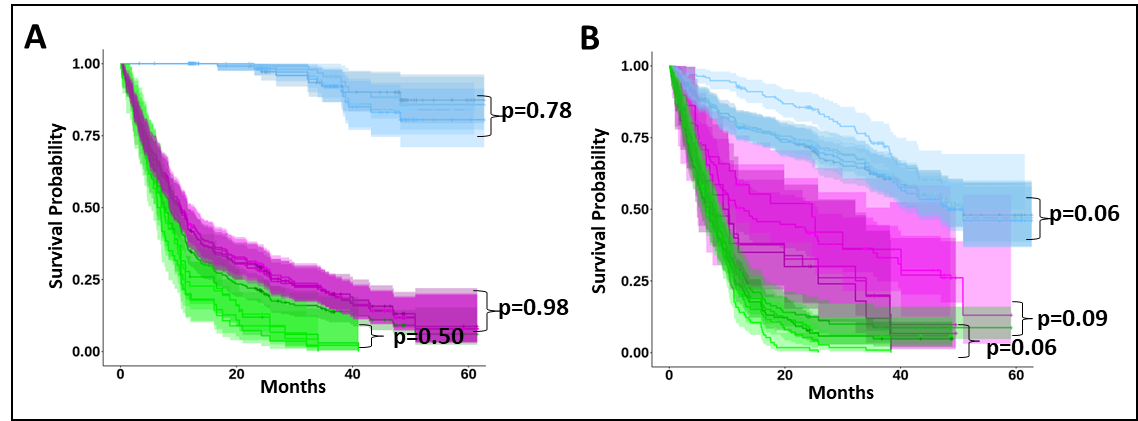


Supplemental Figure 12. Survival curve plots for the robustness of supervised clustering. The genomic subgroups (green: cluster 1; purple: cluster 2; blue: cluster 3) were compared among different k-fold cross-validations of based on genomic common variants (A) and rare variants (B).


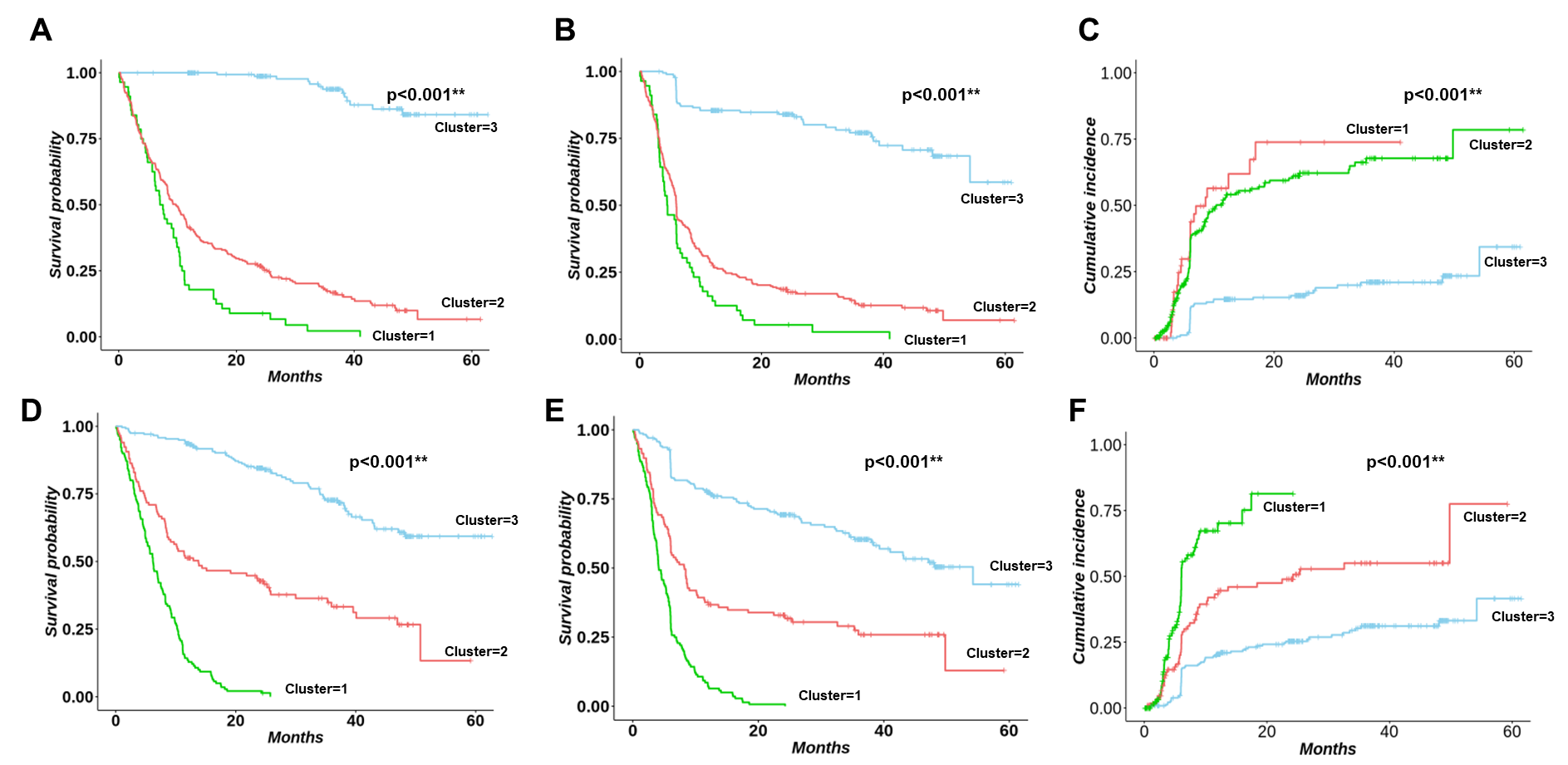


Supplemental Figure 13. Survival curve plots of different survival outcomes for supervised clustering. The clustering subgroups are based on supervised clustering of genomic common variants (A: OS; B: DFS; C: REL) and rare variants (D: OS; E: DFS; F: REL).


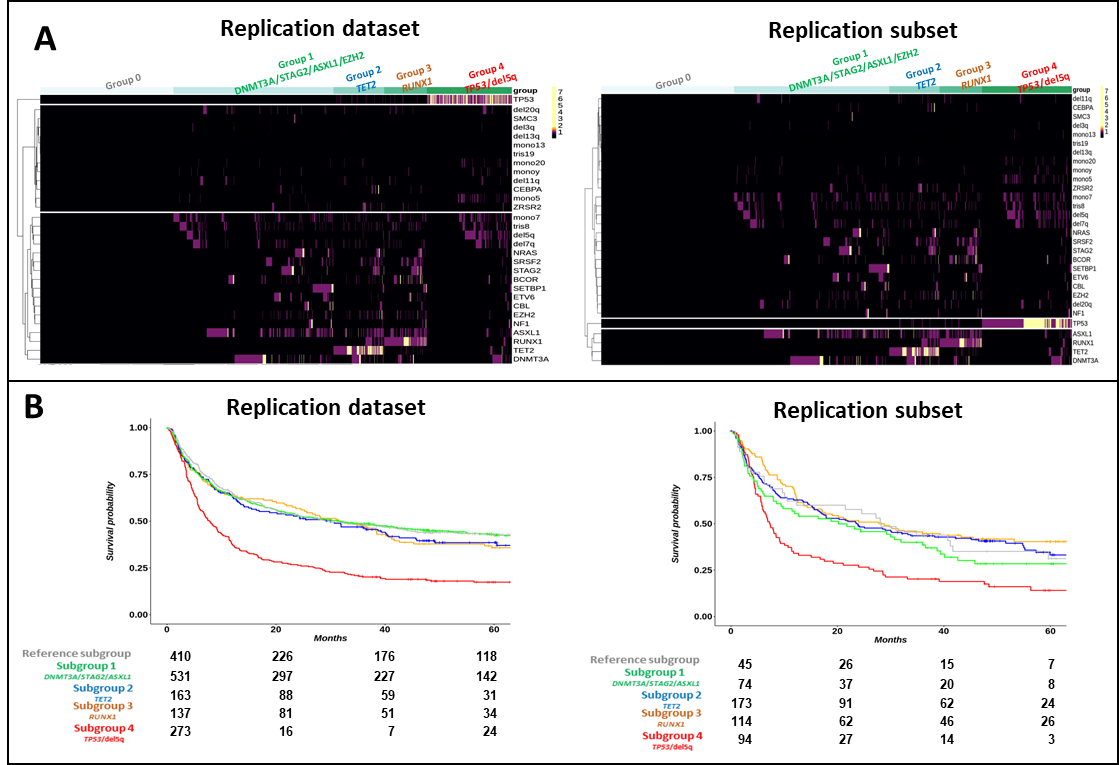


Supplemental Figure 14. Replication of genomic subgroups based on somatic recurrent mutations in an independent MDS cohort.

**A**. The heatmaps of MDS genomic subgroups respectively using recurrent genomic alterations and K-means clustering in replication dataset and subset. Reference subgroup was defined as samples with recurrent somatic mutations and cytogenetic abnormalities. **B**. The survival curves of MDS genomic subgroups respectively using recurrent genomic alterations and K-means clustering in replication dataset and subset.


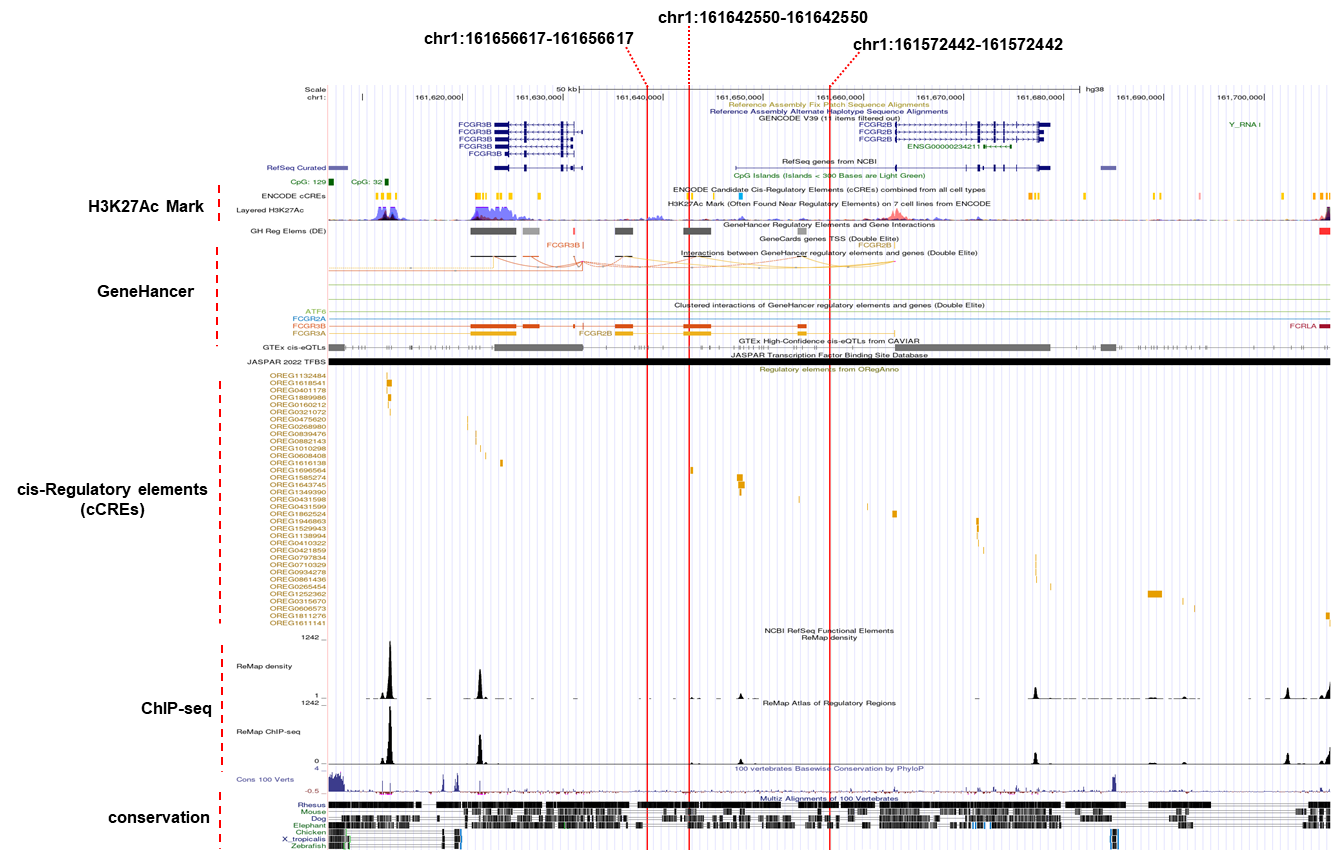


Supplemental Figure 15. The molecular signatures FCGR3B/FCGR2B in genomic subgroup are mutated in the region of known gene enhancer and transcription factor binding site.


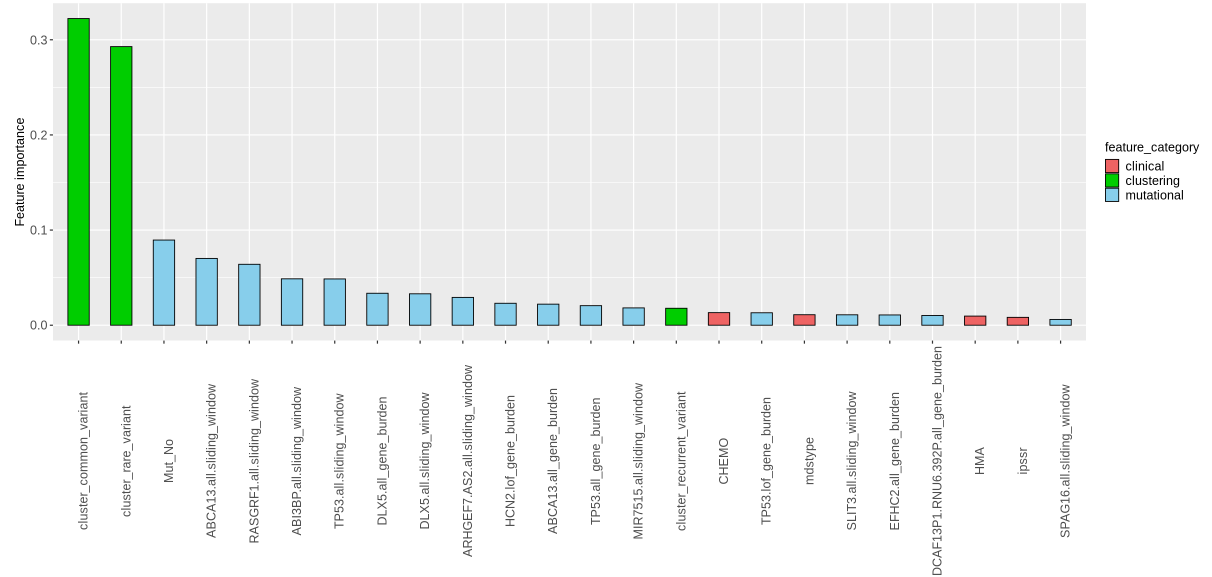


Supplemental Figure 16. Summary of genomic clustering subgroups and genomic association candidates in Random Forest survival (RSF) models based on whole cohort. The variable importance is ranked from the most important to the least important variables on post-transplant overall survival. The green color highlights genomic clustering subgroups and the blue color highlights genomic association candidates, while the red color highlights clinical variables.

Supplemental Table 1. Known prognostic genes from previous clinical studies.

| Gene symbol | Reference |
| --- | --- |
| *TP53,JAK2,RAS* | Prognostic Mutations in Myelodysplastic Syndrome after Stem-Cell Transplantation. N Engl J Med. 2017 Feb 9;376(6):536-547.  Genetic abnormalities in myelodysplasia and secondary acute myeloid leukemia: impact on outcome of stem cell transplantation. Blood. 2017 Apr 27;129(17):2347-2358. |
| *EZH2, ETV6* | Allogeneic hematopoietic stem cell transplantation for MDS and CMML: recommendations from an international expert panel. Blood, 2017 Mar 30;129(13), 1753-1762. |
| *RUNX1, ASXL1* | Clinical Effects of Driver Somatic Mutations on the Outcomes of Patients With Myelodysplastic Syndromes Treated With Allogeneic Hematopoietic Stem-Cell Transplantation. J Clin Oncol, 2016 Oct 20; 34(30), 3627-3637. |
| *TET2, DNMT3A* | Somatic mutations predict poor outcome in patients with myelodysplastic syndrome after hematopoietic stem-cell transplantation. J Clin Oncol. 2014 Sep 1;32(25):2691-8. |

Supplemental Table 2. Clinical variable selection for overall survival.

| **Variable** | **Beta** | **HR** | **SE** | **P-value** |
| --- | --- | --- | --- | --- |
| IPSSR=intermediate | 0.31067 | 1.36434 | 0.15148 | 0.0403 |
| IPSSR=high, very high | 0.7072 | 2.02831 | 0.1654 | 1.91E-05 |
| MDStype = therapy related | -0.12086 | 0.88616 | 0.14808 | 0.4144 |
| MDStype = secondary | -0.73318 | 0.48038 | 0.45572 | 0.1076 |
| HMA = Yes | 0.28684 | 1.33221 | 0.15689 | 0.0675 |
| CHEMO = Yes | 0.28975 | 1.33609 | 0.19507 | 0.1374 |

Reference groups: IPSSR = low, very low, MDS-type = de novo, HMA = none, CHEMO = none

P-values are adjusted by Bonferroni correction

Supplemental Table 3. Demographic characteristics of whole cohort.

| **Characteristics** | Patients |
| --- | --- |
| **No. of patients** | 494 |
| **No. of centers** | 93 |
| **Patient age (year) - median (min-max)** | 66 (22-78) |
| **Sex - no. (%)** |  |
| Male | 315 (64) |
| **Race/ethnicity - no. (%)** |  |
| Caucasian, non-Hispanic | 494 (100) |
| **KPS - no. (%)** |  |
| 0-90 | 251(51) |
| **HCT-CI- no. (%)** |  |
| 0-2 | 171(35) |
| 3+ | 315(64) |
| Missing | 8(2) |
| **Pre-transplant therapies/ (%)** |  |
| HMA alone | 340 (69) |
| Chemo alone | 15 (3) |
| HMA plus Chemo | 34 (7) |
| Neither | 93 (19) |
| Missing | 12 (2) |
| **MDS IPSS-R score pre transplant - no. (%)** |  |
| Very low | 57 (12) |
| Low | 123 (25) |
| Intermediate | 160 (32) |
| High | 74 (15) |
| Very high | 22 (4) |
| Missing | 58 (12) |
| **Time from diagnosis to HCT (month) - median (range)** | 18(2-263) |
| **Donor type - no. (%)** |  |
| HLA-identical sibling | 65 (13) |
| Other related | 32 (6) |
| Well-matched unrelated (8/8) | 353 (71) |
| Partially-matched unrelated (7/8) | 39 (8) |
| Mis-matched unrelated (≤6/8) or unknown | 5 (1) |
| **stem cell source - no. (%)** |  |
| Bone marrow | 59(12) |
| Peripheral blood | 435(88) |
| **regimen intensity - no. (%)** |  |
| Myeloablative | 127(26) |
| Reduced intensity | 308(62) |
| Non-myeloablative | 44(9) |
| Missing | 15(3) |
| **Year of HCT - no. (%)** |  |
| 2014 | 91(18) |
| 2015 | 156(32) |
| 2016 | 118(24) |
| 2017 | 122(25) |
| 2018 | 7(1) |
| **Median follow up of survivors (months) - median (range)** | 34.5(3.2-62.7) |

Supplemental Table 4. Demographic characteristics of subset cohort.

| **Characteristic** | MDS patients without known prognostic genes |
| --- | --- |
| **No. of patients** | 301 |
| **No. of centers** | 81 |
| **Patient age (year) - median (min-max)** | 65.84 (22.58-78.56) |
| **Sex - no. (%)** |  |
| Male | 190 (63) |
| **Race/ethnicity - no. (%)** |  |
| Caucasian, non-Hispanic | 301(100) |
| **KPS - no. (%)** |  |
| 0~90 | 154(51) |
| **HCT-CI- no. (%)** |  |
| 0 | 104(34) |
| 3 | 191(64) |
| Missing | 6(2) |
| **Pre-transplant therapies/ (%)** |  |
| HMA alone yes/no | 198(66) |
| Chemo alone | 12 (4) |
| Both | 24 (8) |
| Neither | 58 (19) |
| Missing | 9 (3) |
| **MDS IPSS-R score pre transplant - no. (%)** |  |
| Very low | 42 (14) |
| Low | 67 (22) |
| Intermediate | 101 (34) |
| High | 38 (13) |
| Very high | 10 (3) |
| Missing | 42 (14) |
| **Time from diagnosis to HCT (month) - median (min-max)** | 18(2-263) |
| **Donor age (year) - median (min-max)** | 34(16-70) |
| **Donor type - no. (%)** |  |
| HLA-identical sibling | 31(10) |
| Other related | 16 (5) |
| Well-matched unrelated (8/8) | 224 (75) |
| Partially-matched unrelated (7/8) | 26 (9) |
| Mis-matched unrelated (≤6/8) or unknown | 4 (1) |
| **graft type - no. (%)** |  |
| Bone marrow | 44(15) |
| Peripheral blood | 256(85) |
| **regimen intensity - no. (%)** |  |
| Myeloablative | 82(27) |
| Reduced intensity | 185(62) |
| Non-myeloablative | 24(8) |
| Missing | 9(3) |
| **Year of HCT - no. (%)** |  |
| 2014 | 56(19) |
| 2015 | 93(31) |
| 2016 | 67(22) |
| 2017 | 78(26) |
| 2018 | 6(2) |
| **Median follow up of survivors (months) - median (range)** | 20.8(0.1-61.0) |

Supplemental Table 5. The hematologic malignancies AML/MDS and lymphoma cohort in TCGA database.

| Cancer type | TCGA Study ID | Patient No | References |
| --- | --- | --- | --- |
| AML/MDS | aml_ohsu_2018 | 672 | Functional genomic landscape of acute myeloid leukaemia. Nature. 2018 Oct;562(7728):526-531. |
|  | aml_target_2018_pub | 1025 | The molecular landscape of pediatric acute myeloid leukemia reveals recurrent structural alterations and age-specific mutational interactions. Nature Medicine 24(1):103-112 |
|  | laml_tcga_pan_can_atlas_2018 | 200 | An Integrated TCGA Pan-Cancer Clinical Data Resource to Drive High-Quality Survival Outcome Analytics.Cell. 2018 Apr 5;173(2):400-416.e11. |
|  | laml_tcga_pub | 200 | Multiomic Integration of Public Oncology Databases in Bioconductor.JCO Clin Cancer Inform. 2020 Oct;4:958-971. |
|  | mnm_washu_2016 | 136 | TP53 and Decitabine in Acute Myeloid Leukemia and Myelodysplastic Syndromes. N Engl J Med. 2016 Nov 24;375(21):2023-2036. |
| Lymphoma | all_phase2_target_2018_pub | 1978 | The genetic basis and cell of origin of mixed phenotype acute leukaemia. Nature. 562(7727):373-379. |
|  | dlbcl_dfci_2018 | 135 | Molecular subtypes of diffuse large B cell lymphoma are associated with distinct pathogenic mechanisms and outcomes. Nat Med. 2018 May;24(5):679-690. |
|  | dlbc_tcga_pan_can_atlas_2018 | 48 | An Integrated TCGA Pan-Cancer Clinical Data Resource to Drive High-Quality Survival Outcome Analytics.Cell. 2018 Apr 5;173(2):400-416.e11. |
|  | dlbc_tcga | 48 | Oncogenic Signaling Pathways in The Cancer Genome Atlas.Cell. 2018 Apr 5;173(2):321-337.e10. |
|  | mbn_mdacc_2013 | 760 | Pathognomonic and epistatic genetic alterations in B-cell non-Hodgkin lymphoma,bioRxiv. |
|  | nhl_bcgsc_2011 | 14 | Frequent mutation of histone-modifying genes in non-Hodgkin lymphoma.Nature. 2011 Jul 27;476(7360):298-303. |
|  | pcnsl_mayo_2015 | 19 | Genome-Wide Analysis Uncovers Novel Recurrent Alterations in Primary Central Nervous System Lymphomas.Clin Cancer Res. 2015 Sep 1;21(17):3986-94. |

| **MDS Cohort** | **Analysis type** | **Variant type** | **ID** | **Number of mutation carriers** | **Adjusted** | | |
| --- | --- | --- | --- | --- | --- | --- | --- |
|  |  |  |  |  | **HR** | **95%CI** | **p_value** |
| **Whole cohort** | **Gene-based overall survival outcome association test** | **Somatic nonsynonymous variants** | TP53 | 52 | 2.19 | 1.59-3.01 | 1.51E-06 |
|  |  |  | HCN2 | 5 | 5.84 | 2.38-14.34 | 1.19E-04 |
|  |  | **All somatic variants** | TP53 | 54 | 2.33 | 1.75-3.10 | 6.24E-09 |
|  |  |  | EFHC2 | 8 | 4.43 | 2.55-7.68 | 1.18E-07 |
|  |  |  | ABCA13 | 39 | 1.92 | 1.50-2.45 | 2.52E-07 |
|  |  |  | DCAF13P1.RNU6.392P | 8 | 6.21 | 3.03-12.75 | 6.43E-07 |
|  |  |  | DLX5 | 5 | 8.99 | 3.60-22.44 | 2.54E-06 |
|  | **Sliding window based overall survival outcome association test** | **All somatic variants** | RASGRF1 | 5 | 27.39 | 10.44-71.89 | 1.76E-11 |
|  |  |  | TP53 | 21 | 3.54 | 2.20-5.71 | 2.07E-07 |
|  |  |  | SLIT3 | 6 | 8.73 | 3.79-20.09 | 3.47E-07 |
|  |  |  | ABI3BP | 5 | 10.43 | 4.20-25.89 | 4.35E-07 |
|  |  |  | MIR7515 | 5 | 10.16 | 4.11-25.11 | 5.18E-07 |
|  |  |  | SPAG16 | 8 | 6.25 | 3.01-12.96 | 8.39E-07 |
|  |  |  | ARHGEF7-AS2 | 5 | 9.58 | 3.77-24.35 | 2.06E-06 |
|  |  |  | ABCA13 | 7 | 6.47 | 2.99-14.01 | 2.21E-06 |
|  |  |  | DLX5 | 5 | 8.99 | 3.60-22.44 | 2.54E-06 |
| **Subset cohort** | **Gene-based overall survival outcome association test** | **Somatic nonsynonymous variants** | DDX11 | 10 | 3.74 | 1.87-7.47 | 1.84E-04 |
|  |  | **All somatic variants** | CHD1 | 7 | 7.31 | 3.33-16.08 | 7.46E-07 |
|  |  |  | RN7SKP174.EI24P4 | 7 | 6.71 | 3.06-14.72 | 1.99E-06 |
|  |  |  | EIF2B2 | 5 | 8.32 | 3.33-20.81 | 5.82E-06 |
|  | **Sliding window based overall survival outcome association test** | **All somatic variants** | RP11-666E17.1-Metazoa_SRP | 6 | 7.69 | 3.24-18.25 | 3.68E-06 |
|  |  |  | EIF2B2 | 5 | 8.63 | 3.42-21.74 | 4.90E-06 |
|  |  |  | RP11-950C14.3 | 5 | 8.63 | 3.42-21.74 | 4.90E-06 |
|  |  |  | SEC14L3 | 7 | 6.01 | 2.73-13.27 | 8.85E-06 |
|  |  |  | bP-2171C21.3 | 7 | 5.71 | 2.61-12.46 | 1.24E-05 |

Supplemental Table 6. Summary of candidate associations with overall survival outcome in multivariate coxph model.

Supplemental Table 7. Permutation validation of candidate associations with overall survival outcome in univariate coxph model.

| **MDS cohort** | **Analysis type** | **Variant type** | **ID** | **Subject No.** | **unadjusted** | |
| --- | --- | --- | --- | --- | --- | --- |
|  |  |  |  |  | p_value  (without permutation) | p_value  (with 10000 permutation) |
| **Whole cohort**  **(all 494 MDS patient subjects)** | Gene-burden based overall survival outcome association test | Somatic nonsynonymous variants | TP53 | 52 | 7.90E-06 | 4.30E-04 |
|  |  |  | HCN2 | 5 | 1.50E-04 | 2.43E-03 |
|  |  | All somatic variants | TP53 | 54 | 5.06E-08 | 1.70E-04 |
|  |  |  | EFHC2 | 8 | 3.13E-07 | 3.90E-04 |
|  |  |  | ABCA13 | 39 | 1.77E-07 | 4.00E-05 |
|  |  |  | DCAF13P1.RNU6.392P | 8 | 2.71E-07 | 3.00E-05 |
|  |  |  | DLX5 | 5 | 1.04E-06 | 2.70E-04 |
|  | Sliding window based overall survival outcome association test | All somatic variants | RASGRF1 | 5 | 5.13E-12 | 1.00E-05 |
|  |  |  | TP53 | 21 | 4.34E-07 | 2.00E-05 |
|  |  |  | SLIT3 | 6 | 1.56E-07 | 9.00E-05 |
|  |  |  | ABI3BP | 5 | 1.72E-07 | 1.00E-04 |
|  |  |  | MIR7515 | 5 | 2.63E-07 | 1.40E-04 |
|  |  |  | SPAG16 | 8 | 2.29E-05 | 5.20E-04 |
|  |  |  | ARHGEF7-AS2 | 5 | 1.50E-05 | 7.70E-04 |
|  |  |  | ABCA13 | 7 | 2.11E-06 | 1.90E-04 |
|  |  |  | DLX5 | 5 | 1.04E-06 | 2.70E-04 |
| **Subset cohort**  **(301 MDS patient subjects without known prognostic gene mutations)** | Gene-burden based overall survival outcome association test | Somatic nonsynonymous variants | DDX11 | 10 | 2.27E-04 | 1.33E-03 |
|  |  | All somatic variants | CHD1 | 7 | 4.39E-07 | 8.00E-05 |
|  |  |  | RN7SKP174.EI24P4 | 7 | 9.13E-07 | 1.00E-04 |
|  |  |  | EIF2B2 | 5 | 1.39E-06 | 2.90E-04 |
|  | Sliding window based overall survival outcome association test | All somatic variants | RP11-666E17.1-Metazoa_SRP | 6 | 4.82E-07 | 1.50E-04 |
|  |  |  | EIF2B2 | 5 | 1.39E-06 | 2.90E-04 |
|  |  |  | RP11-950C14.3 | 5 | 1.39E-06 | 2.90E-04 |
|  |  |  | SEC14L3 | 7 | 1.04E-05 | 4.10E-04 |
|  |  |  | bP-2171C21.3 | 7 | 1.27E-03 | 4.45E-03 |

Supplemental Table 8. The table of genomic subgroups in discovery and replication MDS patient cohort by K-means clustering based on recurrent somatic mutations and cytogenetic abnormalities. No statistical differences of demographic variables between two datasets at cohort level.


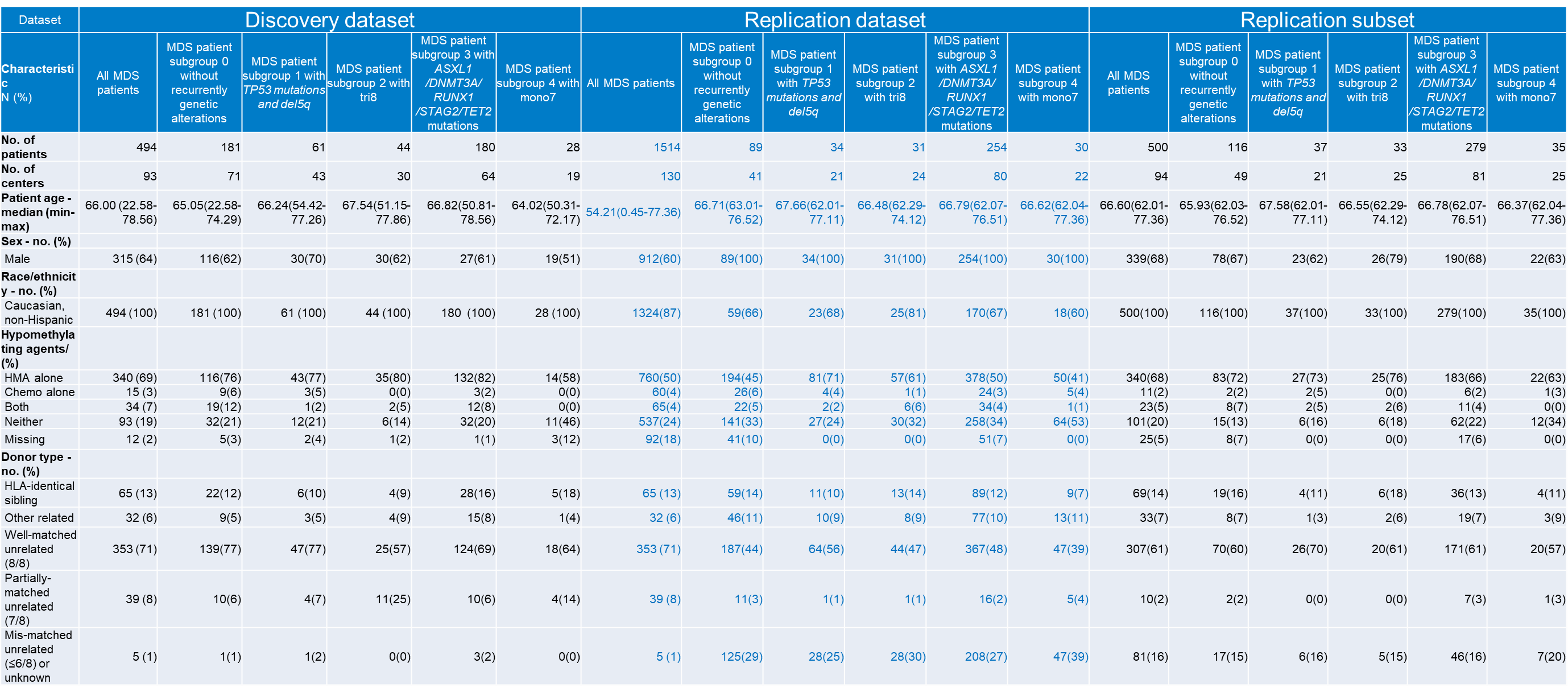


Supplemental Table 9. The prognostic effects of genomic variables on survival outcome in different models.

| Survival Model | Concordance | | |
| --- | --- | --- | --- |
|  | Coxph | Surv.Xgboost | RSF |
| Base model | 0.538 | 0.559 | 0.485 |
| Clinical model | 0.557 | 0.586 | 0.542 |
| Clinical model + genomic association candidates | 0.672 | 0.649 | 0.657 |
| Clinical model + genomic clustering subgroups | 0.819 | 0.745 | 0.829 |
| Clinical model + genomic association candidates+ genomic clustering subgroups | 0.851 | 0.762 | 0.844 |

Supplemental Table 10. The model performance calibration of RSF survival models by Brier scores.

| Survival Model /  Brier score (95%CI) | OS |
| --- | --- |
| Base model | 0.22(0.21-0.22) |
| Clinical model | 0.20 (0.20-0.21) |
| Genomic model | 0.08(0.07-0.09) |
| Full model | 0.07(0.06-0.08) |
| Full model (regimen=Myeloablative) | 0.09(0.07-0.10) |
| Full model (regimen=Reduced intensity) | 0.08(0.07-0.09) |

Supplemental Table 11. Gene annotations for top candidates of overall survival outcome from different association analyses.

| **Gene ID** | **Gene annotations** |
| --- | --- |
| ABCA13 | ATP binding cassette subfamily A member 13 |
| ABI3BP | Target of Nesh-SH3; Fibronectin type III domain containing |
| ARHGEF7 | Rho guanine nucleotide exchange factor 7; Acts as a RAC1 guanine nucleotide exchange factor (GEF) and can induce membrane ruffling. Functions in cell migration, attachment and cell spreading. Promotes targeting of RAC1 to focal adhesions (By similarity). May function as a positive regulator of apoptosis. Downstream of NMDA receptors and CaMKK-CaMK1 signaling cascade, promotes the formation of spines and synapses in hippocampal neurons; Pleckstrin homology domain containing |
| bP-2171C21.3 | Long noncoding RNA |
| DLX5 | Homeobox protein DLX-5; Transcriptional factor involved in bone development. Acts as an immediate early BMP-responsive transcriptional activator essential for osteoblast differentiation. Stimulates ALPL promoter activity in a RUNX2-independent manner during osteoblast differentiation. Stimulates SP7 promoter activity during osteoblast differentiation. Promotes cell proliferation by up-regulating MYC promoter activity. Involved as a positive regulator of both chondrogenesis and chondrocyte hypertrophy in the endochondral skeleton. Binds to the homeodomain-response element of the ALPL an [...] |
| EFHC2 | EF-hand domain-containing family member C2 |
| EIF2B2 | Translation initiation factor eIF-2B subunit beta; Catalyzes the exchange of eukaryotic initiation factor 2-bound GDP for GTP; Belongs to the eIF-2B alpha/beta/delta subunits family |
| HCN2 | Potassium/sodium hyperpolarization-activated cyclic nucleotide-gated channel 2; Hyperpolarization-activated ion channel exhibiting weak selectivity for potassium over sodium ions. Contributes to the native pacemaker currents in heart (If) and in neurons (Ih). Can also transport ammonium in the distal nephron. Produces a large instantaneous current. Modulated by intracellular chloride ions and pH; acidic pH shifts the activation to more negative voltages (By similarity); Cyclic nucleotide gated channels |
| RN7SKP174 | RNA, 7SK Small Nuclear Pseudogene 174 |
| RP11-950C14.3 | Long noncoding RNA |
| SEC14L3 | SEC14-like protein 3; Probable hydrophobic ligand-binding protein; may play a role in the transport of hydrophobic ligands like tocopherol, squalene and phospholipids; SEC14 family |
| SLIT3 | Slit homolog 3 protein; May act as molecular guidance cue in cellular migration, and function may be mediated by interaction with roundabout homolog receptors |
| SPAG16 | Sperm-associated antigen 16 protein; Necessary for sperm flagellar function. Plays a role in motile ciliogenesis. May help to recruit STK36 to the cilium or apical surface of the cell to initiate subsequent steps of construction of the central pair apparatus of motile cilia (By similarity); WD repeat domain containing |
| TP53 | Cellular tumor antigen p53; Acts as a tumor suppressor in many tumor types; induces growth arrest or apoptosis depending on the physiological circumstances and cell type. Involved in cell cycle regulation as a trans-activator that acts to negatively regulate cell division by controlling a set of genes required for this process. One of the activated genes is an inhibitor of cyclin-dependent kinases. Apoptosis induction seems to be mediated either by stimulation of BAX and FAS antigen expression, or by repression of Bcl-2 expression. |

Supplemental Table 12. GTEx annotations of pre-computed whole blood eQTL analyses for candidate genes with significant associations.

| **Gencode Id** | **Gene Symbol** | **Nominal P-Value** | **Empirical P-Value** | **Q-Value** | **aFC** |
| --- | --- | --- | --- | --- | --- |
| **ENSG00000013573.16** | DDX11 | 2.38E-253 | 1.67E-223 | 2.39E-220 | -2.036282 |
| **ENSG00000102606.17** | ARHGEF7 | 2.68E-53 | 1.64E-45 | 1.01E-44 | 0.355417 |
| **ENSG00000100012.11** | SEC14L3 | 2.48E-41 | 1.49E-34 | 6.25E-34 | -1.449792 |
| **ENSG00000119718.10** | EIF2B2 | 9.51E-33 | 1.09E-27 | 3.38E-27 | -0.201426 |
| **ENSG00000099822.2** | HCN2 | 1.05E-10 | 7.91E-07 | 7.57E-07 | -0.521633 |
| **ENSG00000179869.14** | ABCA13 | 7.01E-08 | 0.000258719 | 0.000191371 | 0.686631 |
| **ENSG00000183690.12** | EFHC2 | 8.41E-07 | 0.00145578 | 0.000976552 | -1.97019 |
| **ENSG00000153922.10** | CHD1 | 2.96137E-06 | 0.00620252 | 0.00379529 | -0.204759 |

Supplemental Table 13. Variant deleteriousness annotations for candidate genes.

| **Chr** | **Pos** | **Gene** | **Consequence** | **IMPACT** | **clinvar_sig** |
| --- | --- | --- | --- | --- | --- |
| 7 | 48528298 | ABCA13 | synonymous_variant | LOW | - |
| 7 | 48281444 | ABCA13 | missense_variant | MODERATE | - |
| 7 | 48455085 | ABCA13 | missense_variant | MODERATE | - |
| 7 | 48174792 | ABCA13 | intron_variant | MODIFIER | - |
| 7 | 48197249 | ABCA13 | intron_variant | MODIFIER | - |
| 7 | 48197736 | ABCA13 | intron_variant | MODIFIER | - |
| 7 | 48198077 | ABCA13 | intron_variant | MODIFIER | - |
| 7 | 48204594 | ABCA13 | intron_variant | MODIFIER | - |
| 7 | 48227913 | ABCA13 | intron_variant | MODIFIER | - |
| 7 | 48232504 | ABCA13 | intron_variant | MODIFIER | - |
| 7 | 48282248 | ABCA13 | intron_variant | MODIFIER | - |
| 7 | 48283385 | ABCA13 | intron_variant | MODIFIER | - |
| 7 | 48284108 | ABCA13 | intron_variant | MODIFIER | - |
| 7 | 48293203 | ABCA13 | intron_variant | MODIFIER | - |
| 7 | 48317033 | ABCA13 | intron_variant | MODIFIER | - |
| 7 | 48317038 | ABCA13 | intron_variant | MODIFIER | - |
| 7 | 48318836 | ABCA13 | intron_variant | MODIFIER | - |
| 7 | 48337113 | ABCA13 | intron_variant | MODIFIER | - |
| 7 | 48351058 | ABCA13 | intron_variant | MODIFIER | - |
| 7 | 48351234 | ABCA13 | intron_variant | MODIFIER | - |
| 7 | 48357801 | ABCA13 | intron_variant | MODIFIER | - |
| 7 | 48358585 | ABCA13 | intron_variant | MODIFIER | - |
| 7 | 48372660 | ABCA13 | intron_variant | MODIFIER | - |
| 7 | 48373926 | ABCA13 | intron_variant | MODIFIER | - |
| 7 | 48376106 | ABCA13 | intron_variant | MODIFIER | - |
| 7 | 48398144 | ABCA13 | intron_variant | MODIFIER | - |
| 7 | 48402020 | ABCA13 | intron_variant | MODIFIER | - |
| 7 | 48411831 | ABCA13 | intron_variant | MODIFIER | - |
| 7 | 48449150 | ABCA13 | intron_variant | MODIFIER | - |
| 7 | 48449931 | ABCA13 | intron_variant | MODIFIER | - |
| 7 | 48451521 | ABCA13 | intron_variant | MODIFIER | - |
| 7 | 48452045 | ABCA13 | intron_variant | MODIFIER | - |
| 7 | 48458394 | ABCA13 | intron_variant | MODIFIER | - |
| 7 | 48461478 | ABCA13 | intron_variant | MODIFIER | - |
| 7 | 48487503 | ABCA13 | intron_variant | MODIFIER | - |
| 7 | 48502595 | ABCA13 | intron_variant | MODIFIER | - |
| 7 | 48505610 | ABCA13 | intron_variant | MODIFIER | - |
| 7 | 48520555 | ABCA13 | intron_variant | MODIFIER | - |
| 7 | 48524588 | ABCA13 | intron_variant | MODIFIER | - |
| 7 | 48541982 | ABCA13 | intron_variant | MODIFIER | - |
| 7 | 48549060 | ABCA13 | intron_variant | MODIFIER | - |
| 7 | 48574723 | ABCA13 | intron_variant | MODIFIER | - |
| 7 | 48580768 | ABCA13 | intron_variant | MODIFIER | - |
| 7 | 48624101 | ABCA13 | intron_variant | MODIFIER | - |
| 7 | 48637756 | ABCA13 | intron_variant | MODIFIER | - |
| 7 | 48639334 | ABCA13 | intron_variant | MODIFIER | - |
| 7 | 48639886 | ABCA13 | intron_variant | MODIFIER | - |
| 19 | 585485 | BSG | downstream_gene_variant | MODIFIER | - |
| 5 | 98856931 | CHD1 | intron_variant | MODIFIER | - |
| 5 | 98871353 | CHD1 | intron_variant | MODIFIER | - |
| 5 | 98877746 | CHD1 | intron_variant | MODIFIER | - |
| 5 | 98898050 | CHD1 | intron_variant | MODIFIER | - |
| 5 | 98898054 | CHD1 | intron_variant | MODIFIER | - |
| 5 | 98899854 | CHD1 | intron_variant | MODIFIER | - |
| 5 | 98908441 | CHD1 | intron_variant | MODIFIER | - |
| 5 | 98912718 | CHD1 | intron_variant | MODIFIER | - |
| 5 | 98916609 | CHD1 | intron_variant | MODIFIER | - |
| 12 | 31089977 | DDX11 | synonymous_variant | LOW | - |
| 12 | 31084988 | DDX11 | missense_variant | MODERATE | - |
| 12 | 31085110 | DDX11 | missense_variant | MODERATE | - |
| 12 | 31090065 | DDX11 | missense_variant | MODERATE | - |
| 12 | 31097896 | DDX11 | missense_variant | MODERATE | Uncertain_significance |
| 12 | 31103001 | DDX11 | missense_variant | MODERATE | - |
| 12 | 31097144 | DDX11 | intron_variant | MODIFIER | - |
| 7 | 97019882 | DLX5 | downstream_gene_variant | MODIFIER | - |
| 7 | 97021242 | DLX5 | downstream_gene_variant | MODIFIER | - |
| 7 | 97022047 | DLX5 | 3_prime_UTR_variant | MODIFIER | - |
| 7 | 97026032 | DLX5 | upstream_gene_variant | MODIFIER | - |
| 7 | 97026067 | DLX5 | upstream_gene_variant | MODIFIER | - |
| X | 44179573 | EFHC2 | intron_variant | MODIFIER | - |
| X | 44219853 | EFHC2 | intron_variant | MODIFIER | - |
| X | 44273345 | EFHC2 | intron_variant | MODIFIER | - |
| X | 44273346 | EFHC2 | intron_variant | MODIFIER | - |
| X | 44273363 | EFHC2 | intron_variant | MODIFIER | - |
| X | 44296869 | EFHC2 | intron_variant | MODIFIER | - |
| X | 44296870 | EFHC2 | intron_variant | MODIFIER | - |
| X | 44309899 | EFHC2 | intron_variant | MODIFIER | - |
| X | 44310115 | EFHC2 | intron_variant | MODIFIER | - |
| X | 44333461 | EFHC2 | intron_variant | MODIFIER | - |
| 14 | 75000948 | EIF2B2 | upstream_gene_variant | MODIFIER | - |
| 14 | 75002666 | EIF2B2 | upstream_gene_variant | MODIFIER | - |
| 14 | 75009764 | EIF2B2 | 3_prime_UTR_variant | MODIFIER | - |
| 19 | 605186 | HCN2 | frameshift_variant | HIGH | - |
| 19 | 590043 | HCN2 | missense_variant | MODERATE | - |
| 19 | 603785 | HCN2 | missense_variant | MODERATE | - |
| 19 | 605115 | HCN2 | missense_variant | MODERATE | - |
| 19 | 588907 | HCN2 | upstream_gene_variant | MODIFIER | - |
| 19 | 589054 | HCN2 | upstream_gene_variant | MODIFIER | - |
| 19 | 589688 | HCN2 | upstream_gene_variant | MODIFIER | - |
| 19 | 600070 | HCN2 | intron_variant | MODIFIER | - |
| 19 | 608431 | HCN2 | intron_variant | MODIFIER | - |
| 19 | 609058 | HCN2 | intron_variant | MODIFIER | - |
| 19 | 610247 | HCN2 | intron_variant | MODIFIER | - |
| 17 | 7674196 | TP53 | frameshift_variant | HIGH | - |
| 17 | 7674292 | TP53 | splice_acceptor_variant | HIGH | - |
| 17 | 7674860 | TP53 | splice_donor_variant,coding_sequence_variant,intron_variant | HIGH | - |
| 17 | 7674858 | TP53 | splice_donor_variant | HIGH | - |
| 17 | 7674878 | TP53 | frameshift_variant | HIGH | - |
| 17 | 7674894 | TP53 | stop_gained | HIGH | Pathogenic |
| 17 | 7674905 | TP53 | frameshift_variant | HIGH | - |
| 17 | 7674939 | TP53 | stop_gained | HIGH | Pathogenic |
| 17 | 7674945 | TP53 | stop_gained | HIGH | Pathogenic |
| 17 | 7674972 | TP53 | splice_acceptor_variant | HIGH | - |
| 17 | 7675052 | TP53 | splice_donor_variant | HIGH | - |
| 17 | 7675237 | TP53 | splice_acceptor_variant | HIGH | - |
| 17 | 7675993 | TP53 | splice_donor_variant | HIGH | - |
| 17 | 7676096 | TP53 | frameshift_variant | HIGH | - |
| 17 | 7676183 | TP53 | frameshift_variant | HIGH | - |
| 17 | 7676211 | TP53 | stop_gained | HIGH | Pathogenic |
| 17 | 7674940 | TP53 | synonymous_variant | LOW | - |
| 17 | 7675989 | TP53 | splice_region_variant,intron_variant | LOW | - |
| 17 | 7675990 | TP53 | splice_region_variant,intron_variant | LOW | - |
| 17 | 7675994 | TP53 | splice_region_variant,synonymous_variant | LOW | - |
| 17 | 7687372 | TP53 | splice_region_variant,intron_variant | LOW | - |
| 17 | 7687386 | TP53 | splice_region_variant,5_prime_UTR_variant | LOW | - |
| 17 | 7673764 | TP53 | missense_variant | MODERATE | Pathogenic |
| 17 | 7673793 | TP53 | missense_variant | MODERATE | Likely_pathogenic |
| 17 | 7673796 | TP53 | missense_variant | MODERATE | Pathogenic/Likely_pathogenic |
| 17 | 7673806 | TP53 | missense_variant | MODERATE | Uncertain_significance |
| 17 | 7674211 | TP53 | missense_variant | MODERATE | Likely_pathogenic |
| 17 | 7674221 | TP53 | missense_variant | MODERATE | Pathogenic |
| 17 | 7674241 | TP53 | missense_variant | MODERATE | Likely_pathogenic |
| 17 | 7674250 | TP53 | missense_variant | MODERATE | Conflicting_interpretations_of_pathogenicity |
| 17 | 7674252 | TP53 | missense_variant | MODERATE | Conflicting_interpretations_of_pathogenicity |
| 17 | 7674872 | TP53 | missense_variant | MODERATE | Pathogenic |
| 17 | 7674947 | TP53 | missense_variant | MODERATE | Pathogenic/Likely_pathogenic |
| 17 | 7674953 | TP53 | missense_variant | MODERATE | Conflicting_interpretations_of_pathogenicity |
| 17 | 7675088 | TP53 | missense_variant | MODERATE | Pathogenic |
| 17 | 7673779 | TP53 | missense_variant | MODERATE | Likely_pathogenic |
| 17 | 7674242 | TP53 | missense_variant | MODERATE | Uncertain_significance |
| 17 | 7674263 | TP53 | missense_variant | MODERATE | Pathogenic/Likely_pathogenic |
| 17 | 7674890 | TP53 | missense_variant | MODERATE | Likely_pathogenic |
| 17 | 7674950 | TP53 | missense_variant | MODERATE | Uncertain_significance |
| 17 | 7675076 | TP53 | missense_variant | MODERATE | Conflicting_interpretations_of_pathogenicity |
| 17 | 7675095 | TP53 | missense_variant | MODERATE | Pathogenic |
| 17 | 7675139 | TP53 | missense_variant | MODERATE | Pathogenic |
| 17 | 7675145 | TP53 | missense_variant | MODERATE | Uncertain_significance |
| 17 | 7675217 | TP53 | missense_variant | MODERATE | Likely_pathogenic |
| 17 | 7675218 | TP53 | missense_variant | MODERATE | Conflicting_interpretations_of_pathogenicity |
| 17 | 7676051 | TP53 | missense_variant | MODERATE | Uncertain_significance |
| 17 | 7676055 | TP53 | missense_variant | MODERATE | Likely_pathogenic |
| 17 | 7670726 | TP53 | intron_variant | MODIFIER | - |
| 17 | 7674885 | TP53 | coding_sequence_variant | MODIFIER | - |
| 17 | 7679285 | TP53 | intron_variant | MODIFIER | - |

Supplemental Table 14. Top candidates of overall survival outcome from different association analyses.

(Represent the candidates at top 1~5% rank for each test)

| Test | Gene | Variant count | coef | exp_coef | se_coef | z | p |
| --- | --- | --- | --- | --- | --- | --- | --- |
| Gene-burden based overall survival outcome association test of somatic nonsynonymous variants in whole cohort | *TP53* | 56 | 0.78 | 2.19 | 0.16 | 4.81 | 1.51E-06 |
|  | *HCN2* | 5 | 1.76 | 5.84 | 0.46 | 3.85 | 1.19E-04 |
|  | PRAM1 | 7 | 0.99 | 2.70 | 0.32 | 3.15 | 1.65E-03 |
|  | *DDX11* | 13 | 0.95 | 2.57 | 0.31 | 3.04 | 2.40E-03 |
|  | CHEK2 | 5 | 1.30 | 3.68 | 0.46 | 2.86 | 4.30E-03 |
|  | KRTAP4.11 | 23 | 0.41 | 1.50 | 0.15 | 2.77 | 5.65E-03 |
|  | STEAP1 | 100 | 0.24 | 1.27 | 0.09 | 2.68 | 7.29E-03 |
|  | C16orf96 | 14 | 0.43 | 1.54 | 0.16 | 2.65 | 8.14E-03 |
|  | AC066615.1 | 26 | 0.61 | 1.84 | 0.24 | 2.58 | 9.85E-03 |
| Gene-burden based overall survival outcome association test of all somatic variants in whole cohort | *TP53* | 64 | 0.85 | 2.33 | 0.15 | 5.81 | 6.24E-09 |
|  | EFHC2 | 10 | 1.49 | 4.43 | 0.28 | 5.30 | 1.18E-07 |
|  | *ABCA13* | 51 | 0.65 | 1.92 | 0.13 | 5.16 | 2.52E-07 |
|  | DCAF13P1.RNU6.392P | 8 | 1.83 | 6.21 | 0.37 | 4.98 | 6.43E-07 |
|  | DLX5 | 5 | 2.20 | 8.99 | 0.47 | 4.71 | 2.54E-06 |
|  | KIAA1462.RNU6.598P | 13 | 1.28 | 3.59 | 0.28 | 4.54 | 5.53E-06 |
|  | Metazoa_SRP.RP11.404K5.3 | 5 | 2.08 | 8.02 | 0.46 | 4.50 | 6.88E-06 |
|  | RSF1 | 11 | 1.44 | 4.22 | 0.33 | 4.40 | 1.09E-05 |
|  | RHOBTB1 | 19 | 1.09 | 2.98 | 0.25 | 4.34 | 1.45E-05 |
|  | RPA3 | 9 | 1.48 | 4.40 | 0.34 | 4.33 | 1.52E-05 |
|  | PSMD3 | 5 | 1.96 | 7.10 | 0.46 | 4.23 | 2.32E-05 |
|  | NCAPG2 | 6 | 1.74 | 5.68 | 0.42 | 4.13 | 3.70E-05 |
|  | RP11.167P20.1.ARL2BPP5 | 18 | 1.06 | 2.88 | 0.26 | 4.12 | 3.81E-05 |
|  | CTB.12O2.1.NMUR2 | 16 | 0.94 | 2.57 | 0.23 | 4.06 | 4.93E-05 |
|  | TRPC4.RP11.93G5.1 | 11 | 1.06 | 2.90 | 0.26 | 4.04 | 5.42E-05 |
|  | AC026188.1.SUMF1 | 7 | 1.59 | 4.89 | 0.40 | 3.97 | 7.18E-05 |
|  | USP49 | 7 | 1.25 | 3.48 | 0.32 | 3.96 | 7.64E-05 |
|  | BTBD7 | 7 | 1.32 | 3.73 | 0.34 | 3.91 | 9.34E-05 |
|  | PRKCH | 89 | 0.52 | 1.67 | 0.13 | 3.89 | 9.91E-05 |
|  | SLITRK6.RP11.272M24.2 | 19 | 0.69 | 2.00 | 0.18 | 3.88 | 1.03E-04 |
|  | CTC.321K16.4.AC011431.1 | 6 | 1.61 | 4.98 | 0.42 | 3.81 | 1.42E-04 |
|  | SCART1 | 39 | 0.68 | 1.97 | 0.18 | 3.80 | 1.44E-04 |
|  | CTC.493L21.1.CTC.493L21.2 | 6 | 1.61 | 4.99 | 0.42 | 3.79 | 1.51E-04 |
|  | TMEM104 | 12 | 1.13 | 3.10 | 0.30 | 3.77 | 1.62E-04 |
|  | RNU6.440P.RP11.107N15.1 | 10 | 1.21 | 3.36 | 0.32 | 3.73 | 1.91E-04 |
|  | AXIN2 | 5 | 1.70 | 5.49 | 0.46 | 3.73 | 1.92E-04 |
|  | DPH6.AS1 | 19 | 0.98 | 2.67 | 0.26 | 3.72 | 2.00E-04 |
|  | RP5.912I13.2 | 14 | 1.11 | 3.03 | 0.30 | 3.71 | 2.10E-04 |
|  | SLITRK3 | 6 | 1.53 | 4.64 | 0.41 | 3.70 | 2.14E-04 |
|  | E2F2 | 6 | 1.55 | 4.72 | 0.42 | 3.70 | 2.15E-04 |
|  | UBE2L3 | 8 | 1.29 | 3.62 | 0.35 | 3.69 | 2.24E-04 |
|  | RP3.332B22.1 | 14 | 1.02 | 2.78 | 0.28 | 3.69 | 2.28E-04 |
|  | PROSER2.AS1 | 98 | 0.52 | 1.67 | 0.14 | 3.67 | 2.38E-04 |
|  | RFTN1 | 13 | 1.17 | 3.22 | 0.32 | 3.67 | 2.45E-04 |
|  | RNF13 | 8 | 1.34 | 3.81 | 0.37 | 3.65 | 2.62E-04 |
|  | SYTL2 | 10 | 1.26 | 3.52 | 0.34 | 3.65 | 2.64E-04 |
|  | RP5.965F6.2 | 8 | 1.19 | 3.29 | 0.33 | 3.64 | 2.70E-04 |
|  | RP11.210M15.2 | 7 | 1.37 | 3.95 | 0.38 | 3.64 | 2.73E-04 |
|  | LINC01445.SLC25A5P3 | 9 | 1.32 | 3.73 | 0.36 | 3.63 | 2.89E-04 |
|  | LINC01483 | 22 | 0.81 | 2.26 | 0.23 | 3.61 | 3.12E-04 |
|  | MIMT1.CTC.258N23.3 | 5 | 1.71 | 5.51 | 0.47 | 3.60 | 3.16E-04 |
|  | CTLA4 | 6 | 1.51 | 4.54 | 0.42 | 3.60 | 3.19E-04 |
|  | RPL31P13.RP11.554K11.2 | 5 | 1.64 | 5.17 | 0.46 | 3.58 | 3.38E-04 |
|  | FGD3 | 5 | 1.91 | 6.77 | 0.53 | 3.58 | 3.40E-04 |
|  | RPH3AL | 23 | 0.80 | 2.23 | 0.22 | 3.58 | 3.43E-04 |
|  | PLB1 | 23 | 0.71 | 2.04 | 0.20 | 3.57 | 3.55E-04 |
|  | AC073987.2.AC092568.1 | 23 | 0.71 | 2.04 | 0.20 | 3.56 | 3.74E-04 |
|  | RP3.523K23.2.HCRTR2 | 11 | 1.17 | 3.21 | 0.33 | 3.55 | 3.82E-04 |
|  | WDR86 | 5 | 1.63 | 5.10 | 0.46 | 3.55 | 3.92E-04 |
|  | SLC36A2 | 6 | 1.47 | 4.36 | 0.42 | 3.53 | 4.10E-04 |
|  | *DDX11* | 17 | 0.88 | 2.40 | 0.25 | 3.53 | 4.12E-04 |
|  | CEP57L1 | 5 | 1.61 | 5.01 | 0.46 | 3.51 | 4.52E-04 |
|  | NIPBL | 5 | 1.59 | 4.92 | 0.46 | 3.50 | 4.66E-04 |
|  | RNU4.43P.RP11.351N6.1 | 40 | 0.62 | 1.86 | 0.18 | 3.50 | 4.72E-04 |
|  | TCF7L2.RNU7.165P | 11 | 1.06 | 2.89 | 0.30 | 3.49 | 4.80E-04 |
|  | LINC01310.RPL35P8 | 72 | 0.49 | 1.63 | 0.14 | 3.44 | 5.79E-04 |
|  | GPNMB | 6 | 1.43 | 4.19 | 0.42 | 3.42 | 6.21E-04 |
|  | LINC00989 | 6 | 1.43 | 4.19 | 0.42 | 3.42 | 6.21E-04 |
|  | CH17.174L20.1.RP11.378A12.1 | 7 | 1.42 | 4.14 | 0.42 | 3.40 | 6.63E-04 |
|  | LINC01068.LINC01038 | 12 | 1.11 | 3.05 | 0.33 | 3.40 | 6.84E-04 |
|  | AC004862.6 | 8 | 1.23 | 3.42 | 0.36 | 3.38 | 7.19E-04 |
|  | CTC.493L21.2.CTD.2161E19.1 | 10 | 1.23 | 3.42 | 0.36 | 3.38 | 7.26E-04 |
|  | AC003986.5.AC007091.1 | 20 | 0.86 | 2.35 | 0.25 | 3.38 | 7.29E-04 |
|  | RN7SL255P.RP11.6N13.1 | 9 | 1.32 | 3.73 | 0.39 | 3.37 | 7.40E-04 |
|  | AC079613.1.COL3A1 | 9 | 1.15 | 3.17 | 0.34 | 3.35 | 7.94E-04 |
|  | TOMM5 | 7 | 1.39 | 4.03 | 0.42 | 3.35 | 8.00E-04 |
|  | PHLPP2 | 10 | 1.12 | 3.06 | 0.33 | 3.35 | 8.12E-04 |
|  | MIR6777 | 5 | 1.55 | 4.70 | 0.46 | 3.34 | 8.43E-04 |
|  | CTC.445B21.1 | 9 | 1.22 | 3.37 | 0.36 | 3.34 | 8.47E-04 |
|  | FUT8.CTD.2014B16.1 | 239 | 0.22 | 1.25 | 0.07 | 3.33 | 8.61E-04 |
|  | U3.ATF1P1 | 13 | 0.69 | 1.99 | 0.21 | 3.33 | 8.67E-04 |
|  | FARP1 | 29 | 0.69 | 2.00 | 0.21 | 3.31 | 9.39E-04 |
|  | RP11.696F12.1.RP11.308K2.1 | 26 | 0.62 | 1.86 | 0.19 | 3.29 | 1.02E-03 |
|  | RP11.25E2.1.RP11.116D17.2 | 21 | 0.71 | 2.03 | 0.22 | 3.28 | 1.05E-03 |
|  | C2orf42 | 5 | 1.49 | 4.44 | 0.46 | 3.27 | 1.06E-03 |
|  | RP11.90C4.3.RP1.158P9.1 | 16 | 0.91 | 2.48 | 0.28 | 3.27 | 1.08E-03 |
|  | RP11.583F24.7 | 5 | 1.49 | 4.44 | 0.46 | 3.25 | 1.14E-03 |
|  | RP11.795H16.2.RP11.396N11.1 | 6 | 1.36 | 3.88 | 0.42 | 3.25 | 1.16E-03 |
|  | FAM49B | 11 | 1.05 | 2.86 | 0.32 | 3.25 | 1.17E-03 |
|  | STAB2 | 20 | 0.65 | 1.92 | 0.20 | 3.24 | 1.21E-03 |
|  | HNRNPA1P60.RP11.97I14.1 | 5 | 1.47 | 4.36 | 0.46 | 3.23 | 1.23E-03 |
|  | SETP4.UBE2DNL | 11 | 1.05 | 2.86 | 0.32 | 3.23 | 1.23E-03 |
|  | EEF1A2 | 5 | 1.68 | 5.35 | 0.52 | 3.22 | 1.28E-03 |
|  | PRAM1 | 11 | 0.81 | 2.25 | 0.25 | 3.21 | 1.31E-03 |
|  | HNRNPA3 | 6 | 1.33 | 3.80 | 0.42 | 3.21 | 1.33E-03 |
|  | CACNA1C | 78 | 0.41 | 1.50 | 0.13 | 3.21 | 1.34E-03 |
|  | TSPAN8 | 21 | 0.77 | 2.15 | 0.24 | 3.21 | 1.34E-03 |
|  | ACOT12 | NA | 1.04 | 2.83 | 0.33 | 3.19 | 1.41E-03 |
|  | AB015752.3 | 6 | 1.35 | 3.84 | 0.42 | 3.19 | 1.42E-03 |
|  | VIM.AS1 | 5 | 1.63 | 5.09 | 0.51 | 3.19 | 1.43E-03 |
|  | USP46 | 7 | 1.24 | 3.46 | 0.39 | 3.18 | 1.45E-03 |
|  | FAM188A.SNORA31 | 10 | 1.15 | 3.17 | 0.36 | 3.18 | 1.46E-03 |
|  | RP4.710M16.2 | 5 | 1.45 | 4.26 | 0.45 | 3.18 | 1.46E-03 |
|  | RP11.415C15.2 | 8 | 1.23 | 3.42 | 0.39 | 3.18 | 1.47E-03 |
|  | DOK6 | 99 | 0.38 | 1.46 | 0.12 | 3.18 | 1.48E-03 |
|  | RP11.419L9.1.CTD.2576D5.1 | 6 | 1.33 | 3.77 | 0.42 | 3.18 | 1.48E-03 |
|  | RP11.488I20.8.FRG2HP | 9 | 1.15 | 3.17 | 0.36 | 3.17 | 1.52E-03 |
|  | SSR1 | 5 | 1.45 | 4.27 | 0.46 | 3.17 | 1.53E-03 |
|  | AC011899.10 | 20 | 0.60 | 1.82 | 0.19 | 3.15 | 1.61E-03 |
| Sliding window based overall survival outcome association test of all somatic variants in whole cohort | RASGRF1 | 5 | 3.31 | 27.39 | 0.49 | 6.72 | 1.76E-11 |
|  | *TP53* | 21 | 1.26 | 3.54 | 0.24 | 5.19 | 2.07E-07 |
|  | SLIT3 | 6 | 2.17 | 8.73 | 0.43 | 5.1 | 3.47E-07 |
|  | ABI3BP | 5 | 2.34 | 10.43 | 0.46 | 5.05 | 4.35E-07 |
|  | MIR7515 | 5 | 2.32 | 10.16 | 0.46 | 5.02 | 5.18E-07 |
|  | SPAG16 | 8 | 1.83 | 6.25 | 0.37 | 4.93 | 8.39E-07 |
|  | ARHGEF7-AS2 | 5 | 2.26 | 9.58 | 0.48 | 4.75 | 2.06E-06 |
|  | *ABCA13* | 7 | 1.87 | 6.47 | 0.39 | 4.73 | 2.21E-06 |
|  | DLX5 | 5 | 2.2 | 8.99 | 0.47 | 4.71 | 2.54E-06 |
|  | PWWP2A | 5 | 2.08 | 8.02 | 0.46 | 4.54 | 5.53E-06 |
|  | LINC01605 | 5 | 2.07 | 7.92 | 0.46 | 4.49 | 7.02E-06 |
|  | FAM135B-COL22A1 | 5 | 2.03 | 7.62 | 0.46 | 4.4 | 1.07E-05 |
|  | GLI3-LINC01448 | 5 | 2.1 | 8.13 | 0.48 | 4.39 | 1.12E-05 |
|  | LRGUK | 7 | 1.7 | 5.46 | 0.39 | 4.35 | 1.35E-05 |
|  | RP11-362B23.1-AC073133.2 | 6 | 1.83 | 6.26 | 0.42 | 4.35 | 1.37E-05 |
|  | RPA3 | 5 | 1.97 | 7.19 | 0.46 | 4.32 | 1.57E-05 |
|  | FGF18-CTB-78H18.1 | 5 | 2.05 | 7.76 | 0.48 | 4.3 | 1.69E-05 |
|  | ACA59-CADM1 | 6 | 1.82 | 6.18 | 0.42 | 4.3 | 1.69E-05 |
|  | CTD-2113L7.1 | 5 | 1.96 | 7.09 | 0.46 | 4.26 | 2.03E-05 |
|  | C14orf177 | 6 | 1.86 | 6.42 | 0.44 | 4.25 | 2.12E-05 |
|  | RYR2 | 6 | 1.78 | 5.92 | 0.42 | 4.25 | 2.15E-05 |
|  | MTCO1P55 | 5 | 1.97 | 7.17 | 0.47 | 4.21 | 2.53E-05 |
|  | RP11-736P16.1-RP11-1141N12.1 | 5 | 1.92 | 6.79 | 0.46 | 4.14 | 3.54E-05 |
|  | ARL2BPP5-RP11-541P9.3 | 5 | 1.9 | 6.67 | 0.46 | 4.12 | 3.74E-05 |
|  | RNA5SP189 | 5 | 1.9 | 6.71 | 0.46 | 4.11 | 4.01E-05 |
|  | PRAM1 | 5 | 1.87 | 6.49 | 0.46 | 4.08 | 4.46E-05 |
|  | LTBP2 | 5 | 1.88 | 6.53 | 0.46 | 4.07 | 4.75E-05 |
|  | RNU6-54P-RP11-475F14.1 | 6 | 1.76 | 5.8 | 0.43 | 4.06 | 4.90E-05 |
|  | INPP5A | 5 | 1.84 | 6.32 | 0.46 | 4.02 | 5.73E-05 |
|  | JAM2 | 6 | 1.68 | 5.34 | 0.42 | 4 | 6.36E-05 |
|  | RABGAP1L | 5 | 1.83 | 6.22 | 0.46 | 3.96 | 7.51E-05 |
|  | MYO5A | 5 | 1.81 | 6.12 | 0.46 | 3.95 | 7.90E-05 |
|  | SLITRK6-RP11-272M24.2 | 8 | 1.43 | 4.2 | 0.36 | 3.94 | 8.06E-05 |
|  | RP5-947P14.1-RP11-478L17.1 | 5 | 1.8 | 6.04 | 0.46 | 3.9 | 9.48E-05 |
|  | MDFIC-LINC01392 | 5 | 1.81 | 6.12 | 0.47 | 3.89 | 0.0001 |
|  | RP11-69L16.4 | 5 | 1.78 | 5.95 | 0.46 | 3.88 | 0.00011 |
|  | SMOC2 | 7 | 1.55 | 4.7 | 0.4 | 3.87 | 0.00011 |
|  | CADM1 | 6 | 1.62 | 5.03 | 0.42 | 3.85 | 0.00012 |
|  | AC004911.2-AC073055.2 | 6 | 1.6 | 4.96 | 0.42 | 3.83 | 0.00013 |
|  | CTLA4 | 6 | 1.61 | 5 | 0.42 | 3.83 | 0.00013 |
|  | ARAP3-AC005753.1 | 5 | 1.78 | 5.94 | 0.47 | 3.83 | 0.00013 |
|  | TUBGCP2 | 6 | 1.6 | 4.95 | 0.42 | 3.81 | 0.00014 |
|  | MAP2K2 | 5 | 1.74 | 5.71 | 0.46 | 3.78 | 0.00016 |
|  | RP11-10J21.6-LINC01300 | 5 | 1.75 | 5.76 | 0.46 | 3.77 | 0.00016 |
|  | HNRNPA3 | 6 | 1.57 | 4.81 | 0.42 | 3.77 | 0.00017 |
|  | DCC | 5 | 1.74 | 5.69 | 0.46 | 3.76 | 0.00017 |
|  | SEMA3G | 5 | 1.78 | 5.93 | 0.47 | 3.75 | 0.00017 |
|  | KAZN | 5 | 1.72 | 5.6 | 0.46 | 3.74 | 0.00019 |
|  | DOK6 | 28 | 0.82 | 2.27 | 0.22 | 3.74 | 0.00019 |
|  | CTD-2008N3.1-RP11-445O3.3 | 5 | 1.72 | 5.57 | 0.46 | 3.74 | 0.00019 |
| Gene-burden based overall survival outcome association test of somatic nonsynonymous variants in subset cohort | *DDX11* | 10 | 1.32 | 3.74 | 0.35 | 3.74 | 1.84E-04 |
|  | ARMCX4 | 32 | 0.63 | 1.88 | 0.22 | 2.82 | 4.80E-03 |
|  | AC066615.1 | 18 | 0.71 | 2.04 | 0.28 | 2.51 | 1.20E-02 |
|  | RP11.326C3.14 | 11 | 0.81 | 2.26 | 0.33 | 2.45 | 1.42E-02 |
|  | POTEE | 7 | 0.99 | 2.68 | 0.42 | 2.35 | 1.89E-02 |
|  | CELA1 | 10 | 0.85 | 2.33 | 0.39 | 2.16 | 3.07E-02 |
|  | HMCN2 | 6 | 0.85 | 2.35 | 0.42 | 2.01 | 4.39E-02 |
|  | KRTAP5.5 | 8 | 0.77 | 2.16 | 0.39 | 1.95 | 5.09E-02 |
|  | CGREF1 | 148 | 0.30 | 1.35 | 0.16 | 1.91 | 5.66E-02 |
| Gene-burden based overall survival outcome association test of all somatic variants in subset cohort | *CHD1* | 7 | 1.99 | 7.31 | 0.40 | 4.95 | 7.46E-07 |
|  | RN7SKP174.EI24P4 | 7 | 1.90 | 6.71 | 0.40 | 4.75 | 1.99E-06 |
|  | EIF2B2 | 5 | 2.12 | 8.32 | 0.47 | 4.53 | 5.82E-06 |
|  | RP3.332B22.1 | 5 | 2.15 | 8.58 | 0.48 | 4.51 | 6.47E-06 |
|  | SEC14L3 | 7 | 1.78 | 5.93 | 0.40 | 4.45 | 8.46E-06 |
|  | AC003090.1 | 6 | 1.82 | 6.18 | 0.43 | 4.21 | 2.59E-05 |
|  | SOS1 | 5 | 1.95 | 7.02 | 0.47 | 4.18 | 2.87E-05 |
|  | GS1.519E5.1.RP11.126O22.1 | 5 | 1.91 | 6.74 | 0.47 | 4.10 | 4.08E-05 |
|  | RPA3 | 7 | 1.55 | 4.70 | 0.39 | 3.95 | 7.81E-05 |
|  | OSMR.AS1 | 5 | 1.82 | 6.18 | 0.46 | 3.94 | 8.07E-05 |
|  | CACNA1A.CCDC130 | 10 | 1.37 | 3.94 | 0.35 | 3.91 | 9.34E-05 |
|  | EFHC2 | 5 | 1.80 | 6.03 | 0.46 | 3.87 | 1.08E-04 |
|  | RP11.1084I9.1 | 7 | 1.53 | 4.62 | 0.40 | 3.87 | 1.10E-04 |
|  | SGK223 | 41 | 0.77 | 2.17 | 0.20 | 3.85 | 1.16E-04 |
|  | SLC4A8 | 5 | 1.80 | 6.03 | 0.47 | 3.85 | 1.19E-04 |
|  | TRPC4 | 7 | 1.51 | 4.55 | 0.40 | 3.78 | 1.55E-04 |
|  | MIR7641.1.RP11.886D15.1 | 10 | 1.25 | 3.48 | 0.33 | 3.75 | 1.80E-04 |
|  | MBOAT7 | 5 | 1.77 | 5.89 | 0.47 | 3.74 | 1.85E-04 |
|  | *DDX11* | 12 | 1.19 | 3.28 | 0.32 | 3.72 | 2.01E-04 |
|  | RP11.183I6.2 | 5 | 1.69 | 5.41 | 0.46 | 3.65 | 2.58E-04 |
|  | AC005178.1.WSPAR | 9 | 1.38 | 3.96 | 0.38 | 3.64 | 2.73E-04 |
|  | CYP51A1P1.SRRM1P2 | 52 | 0.68 | 1.97 | 0.19 | 3.64 | 2.76E-04 |
|  | AC105393.2.AC093326.1 | 15 | 1.06 | 2.88 | 0.29 | 3.60 | 3.24E-04 |
|  | SATB1.AS1 | 16 | 1.06 | 2.88 | 0.29 | 3.59 | 3.25E-04 |
|  | RP11.666E17.1.Metazoa_SRP | 22 | 0.92 | 2.52 | 0.26 | 3.58 | 3.43E-04 |
|  | KIAA1462.RNU6.598P | 7 | 1.54 | 4.64 | 0.43 | 3.58 | 3.48E-04 |
|  | AC002383.2.RNU6.274P | 34 | 0.79 | 2.20 | 0.22 | 3.57 | 3.56E-04 |
|  | ASZ1 | 6 | 1.66 | 5.28 | 0.47 | 3.57 | 3.57E-04 |
|  | LINC01499 | 6 | 1.49 | 4.44 | 0.42 | 3.52 | 4.28E-04 |
|  | RN7SKP246.NAMPTP2 | 20 | 0.92 | 2.50 | 0.26 | 3.51 | 4.48E-04 |
|  | CTD.2089N3.3.RNU6.480P | 5 | 1.77 | 5.85 | 0.51 | 3.47 | 5.16E-04 |
|  | RP5.912I13.2 | 8 | 1.33 | 3.80 | 0.39 | 3.41 | 6.50E-04 |
|  | *ABCA13* | 23 | 0.86 | 2.37 | 0.25 | 3.40 | 6.67E-04 |
|  | FARP1 | 8 | 1.35 | 3.84 | 0.40 | 3.39 | 6.91E-04 |
|  | RNU6.967P.CTC.360P9.4 | 5 | 1.56 | 4.76 | 0.46 | 3.38 | 7.22E-04 |
|  | MRPS22 | 6 | 1.45 | 4.25 | 0.43 | 3.37 | 7.40E-04 |
|  | CTD.2366F13.1.RP11.94D20.1 | 9 | 1.27 | 3.56 | 0.38 | 3.37 | 7.48E-04 |
|  | ANKRD7 | 14 | 1.05 | 2.87 | 0.32 | 3.33 | 8.81E-04 |
|  | ADAM33 | 5 | 1.52 | 4.59 | 0.46 | 3.30 | 9.53E-04 |
|  | PDE8B | 8 | 1.30 | 3.69 | 0.40 | 3.28 | 1.03E-03 |
|  | COL6A6 | 7 | 1.39 | 4.03 | 0.43 | 3.28 | 1.05E-03 |
|  | RAD18 | 6 | 1.35 | 3.86 | 0.42 | 3.21 | 1.35E-03 |
|  | ARF1P2 | 10 | 1.11 | 3.05 | 0.35 | 3.20 | 1.39E-03 |
|  | RP11.627G18.4.RP11.535A5.1 | 9 | 1.18 | 3.26 | 0.37 | 3.20 | 1.39E-03 |
|  | RP5.1050E16.2 | 7 | 1.36 | 3.91 | 0.43 | 3.20 | 1.39E-03 |
|  | AC079150.2.AC079150.3 | 5 | 1.58 | 4.85 | 0.50 | 3.18 | 1.46E-03 |
|  | ARMCX4 | 37 | 0.67 | 1.95 | 0.21 | 3.18 | 1.46E-03 |
|  | SSBP2 | 10 | 1.17 | 3.23 | 0.37 | 3.18 | 1.50E-03 |
|  | AP1S2P1.RN7SKP104 | 5 | 1.65 | 5.23 | 0.52 | 3.17 | 1.50E-03 |
|  | PQLC2L.RN7SKP46 | 12 | 1.01 | 2.74 | 0.32 | 3.16 | 1.57E-03 |
|  | RP11.357H3.1.RP11.1O2.1 | 10 | 1.07 | 2.92 | 0.34 | 3.15 | 1.63E-03 |
|  | CASC2 | 11 | 1.21 | 3.34 | 0.38 | 3.15 | 1.64E-03 |
|  | PPIAP1.NCAM2 | 9 | 1.16 | 3.20 | 0.37 | 3.12 | 1.84E-03 |
|  | CTB.12O2.1.NMUR2 | 8 | 1.31 | 3.70 | 0.42 | 3.11 | 1.89E-03 |
|  | LINC00378.RP11.521H3.3 | 22 | 0.77 | 2.16 | 0.25 | 3.10 | 1.94E-03 |
|  | AC008834.1 | 11 | 1.08 | 2.94 | 0.35 | 3.10 | 1.94E-03 |
|  | AC073987.2.AC092568.1 | 11 | 1.03 | 2.79 | 0.33 | 3.10 | 1.95E-03 |
|  | CTD.2091N23.1.POLD2P1 | 13 | 0.94 | 2.56 | 0.30 | 3.09 | 1.99E-03 |
|  | LINC00936 | 9 | 1.23 | 3.41 | 0.40 | 3.09 | 2.01E-03 |
|  | RP11.179K3.2.NEK4P3 | 6 | 1.31 | 3.72 | 0.43 | 3.08 | 2.07E-03 |
|  | RP4.715N11.2 | 5 | 1.63 | 5.11 | 0.53 | 3.06 | 2.25E-03 |
|  | QSOX2 | 6 | 1.31 | 3.71 | 0.43 | 3.05 | 2.29E-03 |
|  | LNPEP | 5 | 1.50 | 4.49 | 0.49 | 3.04 | 2.33E-03 |
|  | AC003984.1.AC093716.1 | 24 | 0.76 | 2.13 | 0.25 | 3.03 | 2.44E-03 |
|  | COX7A2P2.RP11.431J17.1 | 7 | 1.20 | 3.32 | 0.40 | 3.03 | 2.45E-03 |
|  | RP11.542F9.1.RP11.406O16.1 | 12 | 1.03 | 2.81 | 0.34 | 3.03 | 2.48E-03 |
|  | MIR4686.ASCL2 | 5 | 1.47 | 4.34 | 0.49 | 3.02 | 2.53E-03 |
|  | SUMF1 | 18 | 0.79 | 2.21 | 0.27 | 2.99 | 2.80E-03 |
|  | EHBP1 | 10 | 1.03 | 2.81 | 0.35 | 2.97 | 2.95E-03 |
|  | RNF13 | 6 | 1.27 | 3.57 | 0.43 | 2.97 | 2.95E-03 |
|  | ABCG8 | 9 | 1.10 | 2.99 | 0.37 | 2.97 | 2.98E-03 |
|  | RNA5SP117.HSPA8P6 | 106 | 0.48 | 1.61 | 0.16 | 2.95 | 3.14E-03 |
|  | RFX4 | 48 | 0.58 | 1.78 | 0.20 | 2.94 | 3.32E-03 |
|  | USP46 | 5 | 1.35 | 3.87 | 0.46 | 2.92 | 3.49E-03 |
|  | MIR646HG | 24 | 0.70 | 2.02 | 0.24 | 2.91 | 3.59E-03 |
|  | RP11.306B9.1.RP11.1103G16.1 | 5 | 1.34 | 3.82 | 0.46 | 2.91 | 3.64E-03 |
|  | ZNF516 | 6 | 1.24 | 3.44 | 0.43 | 2.89 | 3.85E-03 |
|  | TMEM104 | 5 | 1.33 | 3.78 | 0.46 | 2.88 | 3.94E-03 |
|  | SRSF10P1.MEX3C | 49 | -0.71 | 0.49 | 0.25 | -2.88 | 3.94E-03 |
|  | RP11.440K22.1.ZBBX | 9 | 1.05 | 2.87 | 0.37 | 2.88 | 3.95E-03 |
|  | CASC18.ST13P3 | 32 | 0.66 | 1.93 | 0.23 | 2.87 | 4.06E-03 |
|  | LUC7L2 | 5 | 1.49 | 4.45 | 0.52 | 2.87 | 4.10E-03 |
|  | STRBP.CRB2 | 6 | 1.23 | 3.42 | 0.43 | 2.86 | 4.19E-03 |
|  | LINC01162.RN7SL542P | 10 | 1.00 | 2.72 | 0.35 | 2.86 | 4.21E-03 |
|  | FAM89A.RP5.1097F14.1 | 21 | 0.74 | 2.09 | 0.26 | 2.86 | 4.25E-03 |
|  | PRKAR2B | 12 | 0.96 | 2.62 | 0.34 | 2.85 | 4.31E-03 |
|  | CTC.425K20.1.RPL7P20 | 5 | 1.32 | 3.73 | 0.46 | 2.85 | 4.43E-03 |
|  | CPNE7 | 8 | 1.11 | 3.02 | 0.39 | 2.83 | 4.60E-03 |
|  | RIMBP2 | 9 | 1.07 | 2.91 | 0.38 | 2.83 | 4.60E-03 |
|  | Y_RNA.RP5.942I16.1 | 5 | 1.30 | 3.67 | 0.46 | 2.83 | 4.68E-03 |
|  | SCART1 | 20 | 0.73 | 2.08 | 0.26 | 2.83 | 4.72E-03 |
|  | RP11.205K6.2.RP11.123K19.1 | 5 | 1.29 | 3.63 | 0.46 | 2.81 | 4.90E-03 |
|  | CELA1 | 12 | 0.98 | 2.66 | 0.35 | 2.81 | 4.91E-03 |
|  | DPH6.AS1 | 11 | 0.98 | 2.67 | 0.35 | 2.81 | 4.94E-03 |
|  | ZNF414 | 5 | 1.47 | 4.34 | 0.52 | 2.81 | 5.02E-03 |
|  | ARHGEF6 | 6 | 1.21 | 3.36 | 0.43 | 2.81 | 5.03E-03 |
|  | HEG1 | 20 | 0.72 | 2.06 | 0.26 | 2.77 | 5.53E-03 |
|  | CAMK4 | 23 | 0.72 | 2.05 | 0.26 | 2.77 | 5.57E-03 |
|  | HSPE1P28 | 5 | 1.28 | 3.59 | 0.46 | 2.76 | 5.70E-03 |
| Sliding window based overall survival outcome association test of all somatic variants in subset cohort | RP11-666E17.1-Metazoa_SRP | 9 | 2.04 | 7.66 | 0.37 | 5.51 | 3.51E-08 |
|  | GLI3-LINC01448 | 5 | 2.47 | 11.81 | 0.50 | 4.97 | 6.57E-07 |
|  | *ABCA13* | 6 | 2.10 | 8.19 | 0.44 | 4.82 | 1.43E-06 |
|  | EIF2B2 | 5 | 2.12 | 8.29 | 0.47 | 4.53 | 6.03E-06 |
|  | RP11-950C14.3 | 5 | 2.12 | 8.29 | 0.47 | 4.53 | 6.03E-06 |
|  | SEC14L3 | 7 | 1.78 | 5.91 | 0.40 | 4.44 | 8.88E-06 |
|  | RPS2P2-SYNDIG1L | 5 | 2.04 | 7.72 | 0.48 | 4.27 | 1.99E-05 |
|  | RP3-332B22.1 | 5 | 2.15 | 8.54 | 0.48 | 4.50 | 6.75E-06 |
|  | SPAG16 | 5 | 2.06 | 7.87 | 0.47 | 4.39 | 1.13E-05 |
|  | SGK223 | 38 | 0.88 | 2.41 | 0.20 | 4.32 | 1.59E-05 |
|  | AC008834.1 | 5 | 2.03 | 7.62 | 0.47 | 4.31 | 1.64E-05 |
|  | RP11-384F7.1-IGSF11 | 11 | 1.41 | 4.08 | 0.32 | 4.36 | 1.28E-05 |
|  | CFTRP2-5_8S_rRNA | 5 | 1.90 | 6.70 | 0.47 | 4.08 | 4.57E-05 |
|  | NLGN4X | NA | 4.38 | 79.95 | 1.11 | 3.93 | 8.38E-05 |
|  | RP11-195B3.1-RP11-482E14.1 | 5 | 1.94 | 6.93 | 0.47 | 4.14 | 3.44E-05 |
|  | IGHV1OR15-6 | 7 | 1.57 | 4.80 | 0.40 | 3.90 | 9.44E-05 |
|  | RP11-440K22.1-ZBBX | 5 | 1.93 | 6.86 | 0.47 | 4.06 | 4.99E-05 |
|  | ARMCX4 | 31 | 0.84 | 2.31 | 0.23 | 3.67 | 2.42E-04 |
|  | KCNA3-Y_RNA | 7 | 1.75 | 5.76 | 0.43 | 4.06 | 4.90E-05 |
|  | ZNF140 | 5 | 1.83 | 6.24 | 0.47 | 3.93 | 8.48E-05 |
|  | PARK2 | 5 | 1.89 | 6.62 | 0.47 | 4.06 | 4.93E-05 |
|  | CCL5 | 5 | 1.81 | 6.09 | 0.47 | 3.81 | 1.37E-04 |
|  | RP11-467L19.16 | 7 | 1.38 | 3.97 | 0.40 | 3.47 | 5.22E-04 |
|  | AC124997.1 | 5 | 1.62 | 5.05 | 0.47 | 3.47 | 5.29E-04 |
|  | CACNA1A-CCDC130 | 10 | 1.30 | 3.66 | 0.37 | 3.51 | 4.53E-04 |
|  | ANHX | 5 | 1.71 | 5.55 | 0.47 | 3.61 | 3.08E-04 |
|  | PRKCB | 30 | 0.78 | 2.17 | 0.22 | 3.48 | 5.10E-04 |
|  | RP11-202A13.1 | 11 | 1.17 | 3.21 | 0.32 | 3.64 | 2.77E-04 |
|  | SPPL3 | 11 | 1.17 | 3.23 | 0.33 | 3.53 | 4.23E-04 |
|  | LA16c-444G7.1-ZNF469 | 5 | 1.58 | 4.85 | 0.46 | 3.40 | 6.76E-04 |
|  | RNU6-967P-CTC-360P9.4 | 5 | 1.56 | 4.74 | 0.46 | 3.37 | 7.49E-04 |
|  | RN7SKP181-RP11-315L6.1 | 7 | 1.40 | 4.05 | 0.42 | 3.31 | 9.35E-04 |
|  | LINC00378-RP11-521H3.3 | 21 | 0.83 | 2.30 | 0.25 | 3.34 | 8.28E-04 |
|  | PHACTR1 | 7 | 1.39 | 4.02 | 0.39 | 3.52 | 4.26E-04 |
|  | ZMAT3 | 7 | 1.45 | 4.27 | 0.43 | 3.40 | 6.67E-04 |
|  | GRIK2 | 27 | 0.82 | 2.27 | 0.24 | 3.42 | 6.34E-04 |
|  | RNA5SP117-HSPA8P6 | 102 | 0.55 | 1.73 | 0.16 | 3.34 | 8.52E-04 |
|  | RP5-1050E16.2 | 7 | 1.36 | 3.89 | 0.43 | 3.18 | 1.45E-03 |
|  | TSPAN18 | 12 | 1.02 | 2.78 | 0.32 | 3.22 | 1.30E-03 |
|  | CNTNAP2 | 5 | 1.56 | 4.75 | 0.46 | 3.36 | 7.82E-04 |
|  | RP4-715N11.2 | 5 | 1.63 | 5.08 | 0.53 | 3.04 | 2.34E-03 |
|  | ESRRG | 38 | 0.74 | 2.09 | 0.22 | 3.34 | 8.27E-04 |
|  | IFITM3 | 10 | 1.06 | 2.88 | 0.33 | 3.18 | 1.50E-03 |
|  | *CHD1* | 5 | 1.70 | 5.45 | 0.53 | 3.21 | 1.33E-03 |
|  | AC105393.2-AC093326.1 | 14 | 0.99 | 2.70 | 0.30 | 3.26 | 1.13E-03 |
|  | NFAT5 | 5 | 1.47 | 4.36 | 0.49 | 3.01 | 2.57E-03 |
|  | MIR1538 | 5 | 1.47 | 4.36 | 0.49 | 3.01 | 2.57E-03 |
|  | TTC17 | 5 | 1.62 | 5.05 | 0.52 | 3.10 | 1.94E-03 |
|  | bP-2171C21.3 | 8 | 1.14 | 3.13 | 0.39 | 2.92 | 3.46E-03 |
|  | SEMA6D | 22 | 0.73 | 2.08 | 0.26 | 2.87 | 4.12E-03 |

Supplemental Table 15. Patient level and variant level data for *DDX11*, *CHD1*, *ABCA13*, *HCN2* and *TP53* in our MDS cohort.

| UPN | Chr | Position_Start | Ref | Alt | Gene | Variant_Type | cDNA | AA | VAF | Intxsurv(month) |
| --- | --- | --- | --- | --- | --- | --- | --- | --- | --- | --- |
| 410 | chr7 | 48293203 | G | CCCCCCCCC | *ABCA13* | intron_variant |  |  | 0.45 | 12.04 |
| 111 | chr7 | 48317033 | T | TGGGG | *ABCA13* | intron_variant |  |  | 0.11 | 3.09 |
| 315 | chr7 | 48520553 | TAA | T | *ABCA13* | intron_variant |  |  | 0.17 | 0.63 |
| 545 | chr7 | 48520553 | TAA | T | *ABCA13* | intron_variant |  |  | 0.16 | 4.9 |
| 549 | chr7 | 48520553 | TAA | T | *ABCA13* | intron_variant |  |  | 0.16 | 6.78 |
| 652 | chr7 | 48520553 | TAA | T | *ABCA13* | intron_variant |  |  | 0.21 | 1.97 |
| 919 | chr7 | 48520553 | TAA | T | *ABCA13* | intron_variant |  |  | 0.12 | 0.66 |
| 109 | chr7 | 48351058 | C | T | *ABCA13* | intron_variant |  |  | 0.15 | 3.98 |
| 109 | chr7 | 48398144 | T | G | *ABCA13* | intron_variant |  |  | 0.041 | 3.98 |
| 111 | chr7 | 48317034 | GGTGT | G | *ABCA13* | intron_variant |  |  | 0.11 | 3.09 |
| 123 | chr7 | 48198077 | T | G | *ABCA13* | intron_variant |  |  | 0.23 | 32.2 |
| 125 | chr7 | 48351234 | G | A | *ABCA13* | intron_variant |  |  | 0.14 | 2.3 |
| 252 | chr7 | 48487503 | C | T | *ABCA13* | intron_variant |  |  | 0.073 | 49.57 |
| 272 | chr7 | 48451521 | G | T | *ABCA13* | intron_variant |  |  | 0.067 | 25.76 |
| 300 | chr7 | 48174792 | T | C | *ABCA13* | intron_variant |  |  | 0.32 | 7.01 |
| 300 | chr7 | 48574723 | A | G | *ABCA13* | intron_variant |  |  | 0.33 | 7.01 |
| 311 | chr7 | 48580768 | G | T | *ABCA13* | intron_variant |  |  | 0.081 | 5.1 |
| 315 | chr7 | 48197736 | C | T | *ABCA13* | intron_variant |  |  | 0.29 | 0.63 |
| 335 | chr7 | 48549060 | A | T | *ABCA13* | intron_variant |  |  | 0.085 | 11.15 |
| 477 | chr7 | 48282248 | A | G | *ABCA13* | intron_variant |  |  | 0.28 | 8.26 |
| 493 | chr7 | 48639334 | A | G | *ABCA13* | intron_variant |  |  | 0.23 | 36.81 |
| 529 | chr7 | 48376106 | T | A | *ABCA13* | intron_variant |  |  | 0.3 | 10.23 |
| 529 | chr7 | 48452045 | A | G | *ABCA13* | intron_variant |  |  | 0.26 | 10.23 |
| 532 | chr7 | 48204594 | G | C | *ABCA13* | intron_variant |  |  | 0.32 | 7.07 |
| 549 | chr7 | 48197249 | C | T | *ABCA13* | intron_variant |  |  | 0.087 | 6.78 |
| 615 | chr7 | 48402020 | T | C | *ABCA13* | intron_variant |  |  | 0.083 | 26.81 |
| 628 | chr7 | 48639886 | G | A | *ABCA13* | intron_variant |  |  | 0.091 | 49.05 |
| 634 | chr7 | 48624101 | G | T | *ABCA13* | intron_variant |  |  | 0.19 | 4.57 |
| 639 | chr7 | 48284108 | A | G | *ABCA13* | intron_variant |  |  | 0.74 | 10.39 |
| 639 | chr7 | 48411831 | G | C | *ABCA13* | intron_variant |  |  | 0.096 | 10.39 |
| 639 | chr7 | 48524587 | CA | C | *ABCA13* | intron_variant |  |  | 0.22 | 10.39 |
| 677 | chr7 | 48461477 | GT | G | *ABCA13* | intron_variant |  |  | 0.05 | 8.32 |
| 735 | chr7 | 48227913 | A | T | *ABCA13* | intron_variant |  |  | 0.16 | 2.93 |
| 739 | chr7 | 48502595 | C | T | *ABCA13* | intron_variant |  |  | 0.13 | 49.24 |
| 794 | chr7 | 48372660 | G | A | *ABCA13* | intron_variant |  |  | 0.3 | 6.18 |
| 822 | chr7 | 48358585 | G | A | *ABCA13* | intron_variant |  |  | 0.14 | 4.61 |
| 822 | chr7 | 48505610 | A | G | *ABCA13* | intron_variant |  |  | 0.056 | 4.61 |
| 843 | chr7 | 48458394 | G | C | *ABCA13* | intron_variant |  |  | 0.18 | 5.33 |
| 891 | chr7 | 48449150 | G | C | *ABCA13* | intron_variant |  |  | 0.16 | 2.07 |
| 903 | chr7 | 48283385 | A | C | *ABCA13* | intron_variant |  |  | 0.069 | 2.37 |
| 903 | chr7 | 48318836 | T | C | *ABCA13* | intron_variant |  |  | 0.77 | 2.37 |
| 903 | chr7 | 48449931 | G | T | *ABCA13* | intron_variant |  |  | 0.044 | 2.37 |
| 905 | chr7 | 48541981 | AC | A | *ABCA13* | intron_variant |  |  | 0.037 | 24.38 |
| 923 | chr7 | 48373926 | T | C | *ABCA13* | intron_variant |  |  | 0.15 | 2.99 |
| 984 | chr7 | 48637756 | C | A | *ABCA13* | intron_variant |  |  | 0.32 | 37.93 |
| 1072 | chr7 | 48337113 | G | A | *ABCA13* | intron_variant |  |  | 0.25 | 37.2 |
| 1097 | chr7 | 48232504 | A | G | *ABCA13* | intron_variant |  |  | 0.056 | 2.24 |
| 1097 | chr7 | 48357801 | G | T | *ABCA13* | intron_variant |  |  | 0.04 | 2.24 |
| 1526 | chr7 | 48455085 | G | A | *ABCA13* | missense_variant | c.12614G>A | p.Arg4205His | 0.062 | 0.95 |
| 144 | chr7 | 48281444 | T | C | *ABCA13* | missense_variant | c.8828T>C | p.Ile2943Thr | 0.06 | 2.7 |
| 599 | chr7 | 48528298 | T | G | *ABCA13* | synonymous_variant | c.14307T>G | p.Gly4769Gly | 0.22 | 3.26 |
| 1102 | chr5 | 98912718 | C | T | *CHD1* | intron_variant |  |  | 0.13 | 2.37 |
| 1559 | chr5 | 98898050 | G | A | *CHD1* | intron_variant |  |  | 0.1 | 0.82 |
| 144 | chr5 | 98908441 | C | A | *CHD1* | intron_variant |  |  | 0.048 | 2.7 |
| 385 | chr5 | 98856931 | C | T | *CHD1* | intron_variant |  |  | 0.17 | 5.49 |
| 388 | chr5 | 98898050 | G | A | *CHD1* | intron_variant |  |  | 0.14 | 11.81 |
| 388 | chr5 | 98898054 | A | G | *CHD1* | intron_variant |  |  | 0.14 | 11.81 |
| 396 | chr5 | 98916609 | G | T | *CHD1* | intron_variant |  |  | 0.15 | 13.85 |
| 515 | chr5 | 98898050 | G | A | *CHD1* | intron_variant |  |  | 0.1 | 1.91 |
| 515 | chr5 | 98898054 | A | G | *CHD1* | intron_variant |  |  | 0.1 | 1.91 |
| 561 | chr5 | 98877746 | A | C | *CHD1* | intron_variant |  |  | 0.076 | 4.87 |
| 662 | chr5 | 98898050 | G | A | *CHD1* | intron_variant |  |  | 0.075 | 43.45 |
| 667 | chr5 | 98899854 | C | T | *CHD1* | intron_variant |  |  | 0.21 | 3.62 |
| 903 | chr5 | 98898054 | A | G | *CHD1* | intron_variant |  |  | 0.1 | 2.37 |
| 988 | chr5 | 98898050 | G | A | *CHD1* | intron_variant |  |  | 0.1 | 36.05 |
| 988 | chr5 | 98898054 | A | G | *CHD1* | intron_variant |  |  | 0.1 | 36.05 |
| 1092 | chr5 | 98871353 | T | G | *CHD1* | intron_variant |  |  | 0.096 | 23.78 |
| 599 | chr12 | 31097144 | C | T | *DDX11* | intron_variant |  |  | 0.11 | 3.26 |
| 315 | chr12 | 31103001 | C | G | *DDX11* | missense_variant | c.2438C>G | p.Ala813Gly | 0.25 | 0.63 |
| 1210 | chr12 | 31084988 | G | C | *DDX11* | missense_variant | c.500G>C | p.Arg167Thr | 0.092 | 6.97 |
| 165 | chr12 | 31084988 | G | C | *DDX11* | missense_variant | c.500G>C | p.Arg167Thr | 0.068 | 3.78 |
| 201 | chr12 | 31084988 | G | C | *DDX11* | missense_variant | c.500G>C | p.Arg167Thr | 0.054 | 23.95 |
| 462 | chr12 | 31084988 | G | C | *DDX11* | missense_variant | c.500G>C | p.Arg167Thr | 0.081 | 4.9 |
| 514 | chr12 | 31084988 | G | C | *DDX11* | missense_variant | c.500G>C | p.Arg167Thr | 0.026 | 6.28 |
| 605 | chr12 | 31084988 | G | C | *DDX11* | missense_variant | c.500G>C | p.Arg167Thr | 0.17 | 12.11 |
| 774 | chr12 | 31084988 | G | C | *DDX11* | missense_variant | c.500G>C | p.Arg167Thr | 0.071 | 11.38 |
| 1064 | chr12 | 31084988 | G | C | *DDX11* | missense_variant | c.500G>C | p.Arg167Thr | 0.082 | 4.8 |
| 717 | chr12 | 31097896 | C | G | *DDX11* | missense_variant | c.1774C>G | p.Gln592Glu | 0.055 | 4.51 |
| 144 | chr12 | 31090065 | G | A | *DDX11* | missense_variant | c.1060G>A | p.Gly354Arg | 0.067 | 2.7 |
| 1047 | chr12 | 31090065 | G | A | *DDX11* | missense_variant | c.1060G>A | p.Gly354Arg | 0.047 | 9.9 |
| 1042 | chr12 | 31085110 | A | G | *DDX11* | missense_variant | c.622A>G | p.Lys208Glu | 0.09 | 36.09 |
| 1210 | chr12 | 31089977 | C | T | *DDX11* | synonymous_variant | c.972C>T | p.Gly324Gly | 0.087 | 6.97 |
| 630 | chr12 | 31089977 | C | T | *DDX11* | synonymous_variant | c.972C>T | p.Gly324Gly | 0.058 | 29.11 |
| 728 | chr12 | 31089977 | C | T | *DDX11* | synonymous_variant | c.972C>T | p.Gly324Gly | 0.073 | 4.05 |
| 1092 | chr12 | 31089977 | C | T | *DDX11* | synonymous_variant | c.972C>T | p.Gly324Gly | 0.08 | 23.78 |
| 315 | chr19 | 605185 | TC | T | *HCN2* | frameshift_variant | c.1184delC | p.Pro395fs | 0.29 | 0.63 |
| 1091 | chr19 | 608431 | A | C | *HCN2* | intron_variant |  |  | 0.14 | 40.1 |
| 221 | chr19 | 600070 | C | A | *HCN2* | intron_variant |  |  | 0.32 | 12.3 |
| 815 | chr19 | 609058 | C | T | *HCN2* | intron_variant |  |  | 0.076 | 13.75 |
| 956 | chr19 | 608431 | A | C | *HCN2* | intron_variant |  |  | 0.052 | 37.17 |
| 840 | chr19 | 590043 | A | C | *HCN2* | missense_variant | c.98A>C | p.Gln33Pro | 0.13 | 3.85 |
| 957 | chr19 | 605115 | A | C | *HCN2* | missense_variant | c.1111A>C | p.Ile371Leu | 0.031 | 7.5 |
| 829 | chr19 | 603785 | G | A | *HCN2* | missense_variant | c.874G>A | p.Val292Met | 0.062 | 8.59 |
| 118 | chr19 | 610244 | GCCT | G | *HCN2* | splice_region_variant | c.1438-9_1438-7delTCC |  | 0.1 | 1.61 |
| 1555 | chr19 | 585485 | A | G | *HCN2* | upstream_gene_variant |  |  | 0.062 | 43.16 |
| 118 | chr19 | 589688 | G | A | *HCN2* | upstream_gene_variant |  |  | 0.24 | 1.61 |
| 208 | chr19 | 585485 | A | G | *HCN2* | upstream_gene_variant |  |  | 0.085 | 3.09 |
| 214 | chr19 | 589054 | G | A | *HCN2* | upstream_gene_variant |  |  | 0.16 | 12.34 |
| 241 | chr19 | 585485 | A | G | *HCN2* | upstream_gene_variant |  |  | 0.15 | 19.31 |
| 346 | chr19 | 589054 | G | A | *HCN2* | upstream_gene_variant |  |  | 0.32 | 12.73 |
| 387 | chr19 | 585485 | A | G | *HCN2* | upstream_gene_variant |  |  | 0.11 | 12.04 |
| 577 | chr19 | 588907 | C | G | *HCN2* | upstream_gene_variant |  |  | 0.29 | 48.09 |
| 672 | chr19 | 585485 | A | G | *HCN2* | upstream_gene_variant |  |  | 0.13 | 1.71 |
| 737 | chr19 | 589054 | G | A | *HCN2* | upstream_gene_variant |  |  | 0.15 | 12.43 |
| 923 | chr19 | 589054 | G | A | *HCN2* | upstream_gene_variant |  |  | 0.1 | 2.99 |
| 956 | chr19 | 589054 | G | A | *HCN2* | upstream_gene_variant |  |  | 0.45 | 37.17 |
| 1099 | chr19 | 585485 | A | G | *HCN2* | upstream_gene_variant |  |  | 0.05 | 24.05 |
| 599 | chr17 | 7676182 | CT | C | *TP53* | frameshift_variant | c.69delA | p.Ala24fs | 0.28 | 3.26 |
| 557 | chr17 | 7674903 | TTC | T | *TP53* | frameshift_variant | c.509_510delGA | p.Arg170fs | 0.1 | 8.13 |
| 269 | chr17 | 7674852 | ACCAGACCT | A | *TP53* | frameshift_variant | c.554_555+6delAGGTCTGG | p.Glu185fs | 0.097 | 28.45 |
| 732 | chr17 | 7674196 | G | GT | *TP53* | frameshift_variant | c.649dupA | p.Thr217fs | 0.1 | 6.25 |
| 886 | chr17 | 7676095 | GC | G | *TP53* | frameshift_variant | c.156delG | p.Trp52fs | 0.23 | 3.32 |
| 547 | chr17 | 7674877 | CA | C | *TP53* | frameshift_variant | c.536delT | p.Val179fs | 0.048 | 48.59 |
| 144 | chr17 | 7670726 | A | T | *TP53* | intron_variant |  |  | 0.3 | 2.7 |
| 686 | chr17 | 7679285 | C | T | *TP53* | intron_variant |  |  | 0.066 | 4.7 |
| 136 | chr17 | 7673793 | G | T | *TP53* | missense_variant | c.710C>A | p.Ala237Asp | 0.076 | 42.8 |
| 682 | chr17 | 7675145 | C | G | *TP53* | missense_variant | c.350G>C | p.Arg117Pro | 0.071 | 1.38 |
| 689 | chr17 | 7675139 | C | A | *TP53* | missense_variant | c.356G>T | p.Arg119Leu | 0.32 | 1.71 |
| 207 | chr17 | 7675088 | C | T | *TP53* | missense_variant | c.407G>A | p.Arg136His | 0.11 | 3.26 |
| 960 | chr17 | 7675088 | C | T | *TP53* | missense_variant | c.407G>A | p.Arg136His | 0.3 | 2.17 |
| 371 | chr17 | 7674221 | G | A | *TP53* | missense_variant | c.625C>T | p.Arg209Trp | 0.16 | 0.95 |
| 543 | chr17 | 7674221 | G | A | *TP53* | missense_variant | c.625C>T | p.Arg209Trp | 0.075 | 1.41 |
| 665 | chr17 | 7674221 | G | A | *TP53* | missense_variant | c.625C>T | p.Arg209Trp | 0.066 | 47.63 |
| 843 | chr17 | 7674221 | G | A | *TP53* | missense_variant | c.625C>T | p.Arg209Trp | 0.18 | 5.33 |
| 273 | chr17 | 7673779 | C | G | *TP53* | missense_variant | c.724G>C | p.Asp242His | 0.1 | 5 |
| 851 | chr17 | 7674250 | C | T | *TP53* | missense_variant | c.596G>A | p.Cys199Tyr | 0.12 | 2.34 |
| 916 | chr17 | 7673796 | C | T | *TP53* | missense_variant | c.707G>A | p.Cys236Tyr | 0.18 | 7.17 |
| 748 | chr17 | 7673764 | C | T | *TP53* | missense_variant | c.739G>A | p.Glu247Lys | 0.1 | 2.8 |
| 1056 | chr17 | 7676055 | C | A | *TP53* | missense_variant | c.197G>T | p.Gly66Val | 0.091 | 10.46 |
| 543 | chr17 | 7675076 | T | C | *TP53* | missense_variant | c.419A>G | p.His140Arg | 0.18 | 1.41 |
| 677 | chr17 | 7675076 | T | C | *TP53* | missense_variant | c.419A>G | p.His140Arg | 0.12 | 8.32 |
| 594 | chr17 | 7674953 | T | A | *TP53* | missense_variant | c.461A>T | p.His154Leu | 0.054 | 45.53 |
| 592 | chr17 | 7674890 | T | C | *TP53* | missense_variant | c.524A>G | p.His175Arg | 0.093 | 48.82 |
| 745 | chr17 | 7674947 | A | G | *TP53* | missense_variant | c.467T>C | p.Ile156Thr | 0.23 | 0.92 |
| 847 | chr17 | 7674947 | A | G | *TP53* | missense_variant | c.467T>C | p.Ile156Thr | 0.26 | 11.41 |
| 820 | chr17 | 7674211 | A | C | *TP53* | missense_variant | c.635T>G | p.Ile212Ser | 0.26 | 7.01 |
| 850 | chr17 | 7674950 | A | C | *TP53* | missense_variant | c.464T>G | p.Leu155Arg | 0.089 | 37.01 |
| 254 | chr17 | 7675218 | T | C | *TP53* | missense_variant | c.277A>G | p.Lys93Glu | 0.26 | 47.93 |
| 805 | chr17 | 7675217 | T | G | *TP53* | missense_variant | c.278A>C | p.Lys93Thr | 0.16 | 8.49 |
| 154 | chr17 | 7674252 | C | T | *TP53* | missense_variant | c.594G>A | p.Met198Ile | 0.047 | 4.08 |
| 367 | chr17 | 7674242 | A | C | *TP53* | missense_variant | c.604T>G | p.Ser202Ala | 0.17 | 8.59 |
| 596 | chr17 | 7674241 | G | A | *TP53* | missense_variant | c.605C>T | p.Ser202Phe | 0.2 | 47.86 |
| 895 | chr17 | 7674241 | G | A | *TP53* | missense_variant | c.605C>T | p.Ser202Phe | 0.25 | 24.08 |
| 1098 | chr17 | 7676051 | G | C | *TP53* | missense_variant | c.201C>G | p.Ser67Arg | 0.14 | 23.95 |
| 124 | chr17 | 7674872 | T | C | *TP53* | missense_variant | c.542A>G | p.Tyr181Cys | 0.036 | 0.3 |
| 241 | chr17 | 7674872 | T | C | *TP53* | missense_variant | c.542A>G | p.Tyr181Cys | 0.23 | 19.31 |
| 645 | chr17 | 7674872 | T | C | *TP53* | missense_variant | c.542A>G | p.Tyr181Cys | 0.18 | 0.66 |
| 1210 | chr17 | 7674263 | A | G | *TP53* | missense_variant | c.583T>C | p.Tyr195His | 0.72 | 6.97 |
| 1091 | chr17 | 7675095 | C | A | *TP53* | missense_variant | c.400G>T | p.Val134Leu | 0.14 | 40.1 |
| 923 | chr17 | 7674885 | C | A | *TP53* | missense_variant | c.529G>T | p.Val177Leu | 0.34 | 2.99 |
| 881 | chr17 | 7674885 | C | T | *TP53* | missense_variant | c.529G>A | p.Val177Met | 0.2 | 24.31 |
| 930 | chr17 | 7674885 | C | T | *TP53* | missense_variant | c.529G>A | p.Val177Met | 0.13 | 0.46 |
| 202 | chr17 | 7673806 | C | T | *TP53* | missense_variant | c.697G>A | p.Val233Met | 0.22 | 24.01 |
| 165 | chr17 | 7674292 | T | C | *TP53* | splice_acceptor_variant | c.556-2A>G |  | 0.25 | 3.78 |
| 262 | chr17 | 7674972 | C | T | *TP53* | splice_acceptor_variant | c.443-1G>A |  | 0.48 | 23.68 |
| 958 | chr17 | 7675237 | C | T | *TP53* | splice_acceptor_variant | c.259-1G>A |  | 0.085 | 4.47 |
| 371 | chr17 | 7675993 | C | A | *TP53* | splice_donor_variant | c.258+1G>T |  | 0.14 | 0.95 |
| 496 | chr17 | 7675052 | C | T | *TP53* | splice_donor_variant | c.442+1G>A |  | 0.11 | 23.52 |
| 886 | chr17 | 7674858 | C | T | *TP53* | splice_donor_variant | c.555+1G>A |  | 0.085 | 3.32 |
| 1526 | chr17 | 7675994 | C | T | *TP53* | splice_region_variant | c.258G>A |  | 0.22 | 0.95 |
| 273 | chr17 | 7675994 | C | T | *TP53* | splice_region_variant | c.258G>A |  | 0.076 | 5 |
| 840 | chr17 | 7687376 | CCCAATCCAGG | C | *TP53* | splice_region_variant | c.-155_-146delCCTGGATTGG |  | 0.19 | 3.85 |
| 580 | chr17 | 7687372 | C | T | *TP53* | splice_region_variant | c.-146+5G>A |  | 0.065 | 47.6 |
| 745 | chr17 | 7675989 | C | T | *TP53* | splice_region_variant | c.258+5G>A |  | 0.22 | 0.92 |
| 748 | chr17 | 7675990 | T | TGG | *TP53* | splice_region_variant | c.258+3_258+4insCC |  | 0.093 | 2.8 |
| 165 | chr17 | 7674945 | G | A | *TP53* | stop_gained | c.469C>T | p.Arg157* | 0.12 | 3.78 |
| 715 | chr17 | 7674894 | G | A | *TP53* | stop_gained | c.520C>T | p.Arg174* | 0.16 | 8.22 |
| 840 | chr17 | 7674894 | G | A | *TP53* | stop_gained | c.520C>T | p.Arg174* | 0.21 | 3.85 |
| 689 | chr17 | 7674939 | C | A | *TP53* | stop_gained | c.475G>T | p.Glu159* | 0.27 | 1.71 |
| 173 | chr17 | 7676211 | C | T | *TP53* | stop_gained | c.41G>A | p.Trp14* | 0.048 | 25.23 |
| 689 | chr17 | 7674940 | C | A | *TP53* | synonymous_variant | c.474G>T | p.Val158Val | 0.27 | 1.71 |

Supplemental Table 16. Gene annotation table for Top genes in supervised clustering analysis of common variants.

| Gene ID | Gene annotation |
| --- | --- |
| ABL2 | Abelson tyrosine-protein kinase 2; Non-receptor tyrosine-protein kinase that plays an ABL1- overlapping role in key processes linked to cell growth and survival such as cytoskeleton remodeling in response to extracellular stimuli, cell motility and adhesion and receptor endocytosis. Coordinates actin remodeling through tyrosine phosphorylation of proteins controlling cytoskeleton dynamics like MYH10 (involved in movement); CTTN (involved in signaling); or TUBA1 and TUBB (microtubule subunits). Binds directly F-actin and regulates actin cytoskeletal structure through its F-actin- bundli [...] |
| ACAP2 | Arf-GAP with coiled-coil, ANK repeat and PH domain-containing protein 2; GTPase-activating protein (GAP) for ADP ribosylation factor 6 (ARF6); Ankyrin repeat domain containing |
| ADAMTS17 | A disintegrin and metalloproteinase with thrombospondin motifs 17; ADAM metallopeptidase with thrombospondin type 1 motif 17 |
| AMY1B | Amylase, alpha 1b; Belongs to the glycosyl hydrolase 13 family |
| ANKRD30B | Ankyrin repeat domain-containing protein 30B; Ankyrin repeat domain containing |
| ANO3 | Anoctamin-3; Has calcium-dependent phospholipid scramblase activity; scrambles phosphatidylcholine and galactosylceramide. Seems to act as potassium channel regulator and may inhibit pain signaling; can facilitate KCNT1/Slack channel activity by promoting its full single-channel conductance at very low sodium concentrations and by increasing its sodium sensitivity (By similarity). Does not exhibit calcium-activated chloride channel (CaCC) activity; Anoctamins |
| ASS1 | Argininosuccinate synthase; One of the enzymes of the urea cycle, the metabolic pathway transforming neurotoxic amonia produced by protein catabolism into inocuous urea in the liver of ureotelic animals. Catalyzes the formation of arginosuccinate from aspartate, citrulline and ATP and together with ASL it is responsible for the biosynthesis of arginine in most body tissues; Belongs to the argininosuccinate synthase family. Type 1 subfamily |
| BANK1 | B-cell scaffold protein with ankyrin repeats; Involved in B-cell receptor (BCR)-induced Ca(2+) mobilization from intracellular stores. Promotes Lyn-mediated phosphorylation of IP3 receptors 1 and 2; Ankyrin repeat domain containing |
| BEND4 | Ben domain-containing protein 4; BEN domain containing 4 |
| C1QB | Complement C1q subcomponent subunit B; C1q associates with the proenzymes C1r and C1s to yield C1, the first component of the serum complement system. The collagen-like regions of C1q interact with the Ca(2+)-dependent C1r(2)C1s(2) proenzyme complex, and efficient activation of C1 takes place on interaction of the globular heads of C1q with the Fc regions of IgG or IgM antibody present in immune complexes |
| C2CD4C | C2 calcium-dependent domain-containing protein 4C; C2 calcium dependent domain containing 4C; Belongs to the C2CD4 family |
| C6orf57 | Succinate dehydrogenase assembly factor 4, mitochondrial; Plays an essential role in the assembly of succinate dehydrogenase (SDH), an enzyme complex (also referred to as respiratory complex II) that is a component of both the tricarboxylic acid (TCA) cycle and the mitochondrial electron transport chain, and which couples the oxidation of succinate to fumarate with the reduction of ubiquinone (coenzyme Q) to ubiquinol. Binds to the flavoprotein subunit SDHA in its FAD-bound form, blocking the generation of excess reactive oxigen species (ROS) and facilitating its assembly with the iron [...] |
| CDH13 | Cadherin-13; Cadherins are calcium-dependent cell adhesion proteins. They preferentially interact with themselves in a homophilic manner in connecting cells; cadherins may thus contribute to the sorting of heterogeneous cell types. May act as a negative regulator of neural cell growth |
| CDH15 | Cadherin-15; Cadherins are calcium-dependent cell adhesion proteins. They preferentially interact with themselves in a homophilic manner in connecting cells; cadherins may thus contribute to the sorting of heterogeneous cell types. M-cadherin is part of the myogenic program and may provide a trigger for terminal muscle differentiation |
| CDH2 | Cadherin-2; Cadherins are calcium-dependent cell adhesion proteins. They preferentially interact with themselves in a homophilic manner in connecting cells; cadherins may thus contribute to the sorting of heterogeneous cell types. Acts as a regulator of neural stem cells quiescence by mediating anchorage of neural stem cells to ependymocytes in the adult subependymal zone: upon cleavage by MMP24, CDH2-mediated anchorage is affected, leading to modulate neural stem cell quiescence. CDH2 may be involved in neuronal recognition mechanism. In hippocampal neurons, may regulate dendritic spi [...] |
| CDH23 | Cadherin related 23; Cadherin-23; Cadherins are calcium-dependent cell adhesion proteins. They preferentially interact with themselves in a homophilic manner in connecting cells. CDH23 is required for establishing and/or maintaining the proper organization of the stereocilia bundle of hair cells in the cochlea and the vestibule during late embryonic/early postnatal development. It is part of the functional network formed by USH1C, USH1G, CDH23 and MYO7A that mediates mechanotransduction in cochlear hair cells. Required for normal hearing |
| CELF2 | CUGBP Elav-like family member 2; RNA-binding protein implicated in the regulation of several post-transcriptional events. Involved in pre-mRNA alternative splicing, mRNA translation and stability. Mediates exon inclusion and/or exclusion in pre-mRNA that are subject to tissue-specific and developmentally regulated alternative splicing. Specifically activates exon 5 inclusion of TNNT2 in embryonic, but not adult, skeletal muscle. Activates TNNT2 exon 5 inclusion by antagonizing the repressive effect of PTB. Acts as both an activator and repressor of a pair of coregulated exons: promotes [...] |
| CLSTN2 | Calsyntenin-2; May modulate calcium-mediated postsynaptic signals; Cadherin related |
| CNTN4 | Contactin-4; Contactins mediate cell surface interactions during nervous system development. Has some neurite outgrowth-promoting activity. May be involved in synaptogenesis; Fibronectin type III domain containing |
| CR1L | Complement component receptor 1-like protein; Complement C3b/C4b receptor 1 like; Belongs to the receptors of complement activation (RCA) family |
| CROCC | Ciliary rootlet coiled-coil, rootletin; Rootletin; Major structural component of the ciliary rootlet, a cytoskeletal-like structure in ciliated cells which originates from the basal body at the proximal end of a cilium and extends proximally toward the cell nucleus (By similarity). Furthermore, is required for the correct positioning of the cilium basal body relative to the cell nucleus, to allow for ciliogenesis. Contributes to centrosome cohesion before mitosis |
| CTSO | Cathepsin O; Proteolytic enzyme possibly involved in normal cellular protein degradation and turnover; Belongs to the peptidase C1 family |
| CWH43 | Cell wall biogenesis 43 c-terminal homolog; PGAP2-interacting protein; Involved in lipid remodeling during GPI-anchor maturation |
| DCUN1D4 | DCN1-like protein 4; Defective in cullin neddylation 1 domain containing 4 |
| DKK2 | Dickkopf-related protein 2; Antagonizes canonical Wnt signaling by inhibiting LRP5/6 interaction with Wnt and by forming a ternary complex with the transmembrane protein KREMEN that promotes internalization of LRP5/6. DKKs play an important role in vertebrate development, where they locally inhibit Wnt regulated processes such as antero- posterior axial patterning, limb development, somitogenesis and eye formation. In the adult, Dkks are implicated in bone formation and bone disease, cancer and Alzheimer disease (By similarity); Belongs to the dickkopf family |
| DLGAP2 | Disks large-associated protein 2; May play a role in the molecular organization of synapses and neuronal cell signaling. Could be an adapter protein linking ion channel to the subsynaptic cytoskeleton. May induce enrichment of PSD-95/SAP90 at the plasma membrane; Belongs to the SAPAP family |
| ENSP00000429240 | annotation not available |
| EPM2A | Laforin; Plays an important role in preventing glycogen hyperphosphorylation and the formation of insoluble aggregates, via its activity as glycogen phosphatase, and by promoting the ubiquitination of proteins involved in glycogen metabolism via its interaction with the E3 ubiquitin ligase NHLRC1/malin. Shows strong phosphatase activity towards complex carbohydrates in vitro, avoiding glycogen hyperphosphorylation which is associated with reduced branching and formation of insoluble aggregates. Dephosphorylates phosphotyrosine and synthetic substrates, such as para- nitrophenylphosphat [...] |
| FAM177A1 | Protein FAM177A1; Family with sequence similarity 177 member A1 |
| FAM213A | Redox-regulatory protein FAM213A; Involved in redox regulation of the cell. Acts as an antioxidant. Inhibits TNFSF11-induced NFKB1 and JUN activation and osteoclast differentiation. May affect bone resorption and help to maintain bone mass. Acts as a negative regulator of macrophage-mediated inflammation by inhibiting macrophage production of inflammatory cytokines, probably through suppression of the MAPK signaling pathway; Belongs to the peroxiredoxin-like FAM213 family. FAM213A subfamily |
| FCGR3B | Low affinity immunoglobulin gamma Fc region receptor III-B; Receptor for the Fc region of immunoglobulins gamma. Low affinity receptor. Binds complexed or aggregated IgG and also monomeric IgG. Contrary to III-A, is not capable to mediate antibody-dependent cytotoxicity and phagocytosis. May serve as a trap for immune complexes in the peripheral circulation which does not activate neutrophils; CD molecules |
| FREM1 | FRAS1-related extracellular matrix protein 1; Extracellular matrix protein that plays a role in epidermal differentiation and is required for epidermal adhesion during embryonic development; Belongs to the FRAS1 family |
| FSTL5 | Follistatin-related protein 5; EF-hand domain containing |
| GALR1 | Galanin receptor type 1; Receptor for the hormone galanin. The activity of this receptor is mediated by G proteins that inhibit adenylate cyclase activity; Belongs to the G-protein coupled receptor 1 family |
| GPD1L | Glycerol-3-phosphate dehydrogenase 1-like protein; Plays a role in regulating cardiac sodium current; decreased enzymatic activity with resulting increased levels of glycerol 3-phosphate activating the DPD1L-dependent SCN5A phosphorylation pathway, may ultimately lead to decreased sodium current; cardiac sodium current may also be reduced due to alterations of NAD(H) balance induced by DPD1L |
| GPX6 | Glutathione peroxidase 6; Selenoproteins |
| HCN4 | Potassium/sodium hyperpolarization-activated cyclic nucleotide-gated channel 4; Hyperpolarization-activated ion channel with very slow activation and inactivation exhibiting weak selectivity for potassium over sodium ions. Contributes to the native pacemaker currents in heart (If) that regulate the rhythm of heart beat. May contribute to the native pacemaker currents in neurons (Ih). May mediate responses to sour stimuli |
| HMP19 | Neuronal vesicle trafficking-associated protein 2; Neuron-specific protein family member 2 |
| HSPA13 | Heat shock 70 kDa protein 13; Has peptide-independent ATPase activity; Heat shock 70kDa proteins |
| IFITM3 | Interferon-induced transmembrane protein 3; IFN-induced antiviral protein which disrupts intracellular cholesterol homeostasis. Inhibits the entry of viruses to the host cell cytoplasm by preventing viral fusion with cholesterol depleted endosomes. May inactivate new enveloped viruses which buds out of the infected cell, by letting them go out with a cholesterol depleted membrane. Active against multiple viruses, including influenza A virus, SARS coronavirus (SARS-CoV), Marburg virus (MARV) and Ebola virus (EBOV), Dengue virus (DNV), West Nile virus (WNV), human immunodeficiency virus [...] |
| INPP5K | Inositol polyphosphate 5-phosphatase K; Inositol 5-phosphatase which acts on inositol 1,4,5- trisphosphate, inositol 1,3,4,5-tetrakisphosphate, phosphatidylinositol 4,5-bisphosphate and phosphatidylinositol 3,4,5-trisphosphate. Has 6-fold higher affinity for phosphatidylinositol 4,5-bisphosphate than for inositol 1,4,5- trisphosphate. Negatively regulates assembly of the actin cytoskeleton. Controls insulin-dependent glucose uptake among inositol 3,4,5-trisphosphate phosphatases; therefore, is the specific regulator for insulin signaling in skeletal muscle (By similarity) |
| KAZN | Kazrin, periplakin interacting protein; Kazrin; Component of the cornified envelope of keratinocytes. May be involved in the interplay between adherens junctions and desmosomes. The function in the nucleus is not known; Sterile alpha motif domain containing |
| KLHL32 | Kelch-like protein 32; Kelch like family member 32; BTB domain containing |
| LDLRAD4 | Low-density lipoprotein receptor class A domain-containing protein 4; Functions as a negative regulator of TGF-beta signaling and thereby probably plays a role in cell proliferation, differentiation, apoptosis, motility, extracellular matrix production and immunosuppression. In the canonical TGF-beta pathway, ZFYVE9/SARA recruits the intracellular signal transducer and transcriptional modulators SMAD2 and SMAD3 to the TGF-beta receptor. Phosphorylated by the receptor, SMAD2 and SMAD3 then form a heteromeric complex with SMAD4 that translocates to the nucleus to regulate transcription. [...] |
| LEMD3 | Inner nuclear membrane protein Man1; Can function as a specific repressor of TGF-beta, activin, and BMP signaling through its interaction with the R-SMAD proteins. Antagonizes TGF-beta-induced cell proliferation arrest; LEM domain containing |
| LRRC55 | Leucine-rich repeat-containing protein 55; Auxiliary protein of the large-conductance, voltage and calcium-activated potassium channel (BK alpha). Modulates gating properties by producing a marked shift in the BK channel's voltage dependence of activation in the hyperpolarizing direction, and in the absence of calcium |
| MAP3K5 | Mitogen-activated protein kinase kinase kinase 5; Serine/threonine kinase which acts as an essential component of the MAP kinase signal transduction pathway. Plays an important role in the cascades of cellular responses evoked by changes in the environment. Mediates signaling for determination of cell fate such as differentiation and survival. Plays a crucial role in the apoptosis signal transduction pathway through mitochondria-dependent caspase activation. MAP3K5/ASK1 is required for the innate immune response, which is essential for host defense against a wide range of pathogens. Me [...] |
| MGAT5 | Alpha-1,3(6)-mannosylglycoprotein beta-1,6-N-acetyl-glucosaminyltransferase; Alpha-1,6-mannosylglycoprotein 6-beta-N-acetylglucosaminyltransferase A; Catalyzes the addition of N-acetylglucosamine in beta 1- 6 linkage to the alpha-linked mannose of biantennary N-linked oligosaccharides. It is one of the most important enzymes involved in the regulation of the biosynthesis of glycoprotein oligosaccharides; Belongs to the glycosyltransferase 18 family |
| MGST2 | Microsomal glutathione S-transferase 2; Can catalyze the production of LTC4 from LTA4 and reduced glutathione. Can catalyze the conjugation of 1-chloro-2,4- dinitrobenzene with reduced glutathione; Microsomal glutathione S-transferases |
| MPPED1 | Metallophosphoesterase domain-containing protein 1; May have metallophosphoesterase activity (in vitro) |
| MROH7 | Maestro heat-like repeat-containing protein family member 7; Maestro heat like repeat family member 7 |
| MTUS2 | Microtubule-associated tumor suppressor candidate 2; Binds microtubules. Together with MAPRE1 may target the microtubule depolymerase KIF2C to the plus-end of microtubules. May regulate the dynamics of microtubules at their growing distal tip; In the C-terminal section; belongs to the MTUS1 family |
| NBPF1 | Neuroblastoma breakpoint family member 1; NBPF member 1 |
| NRG3 | Pro-neuregulin-3, membrane-bound isoform; Direct ligand for the ERBB4 tyrosine kinase receptor. Binding results in ligand-stimulated tyrosine phosphorylation and activation of the receptor. Does not bind to the EGF receptor, ERBB2 or ERBB3 receptors. May be a survival factor for oligodendrocytes; Belongs to the neuregulin family |
| OSBPL10 | Oxysterol-binding protein-related protein 10; Probable lipid transporter involved in lipid countertransport between the endoplasmic reticulum and the plasma membrane. Its ability to bind phosphatidylserine, suggests that it specifically exchanges phosphatidylserine with phosphatidylinositol 4-phosphate (PI4P), delivering phosphatidylserine to the plasma membrane in exchange for PI4P (Probable). Plays a role in negative regulation of lipid biosynthesis. Negatively regulates APOB secretion from hepatocytes. Binds cholesterol and acidic phospholipids. Also binds 25-hydroxycholesterol. Bin [...] |
| PCDH15 | Protocadherin-15; Calcium-dependent cell-adhesion protein. Essential for maintenance of normal retinal and cochlear function; Cadherin related |
| PCNT | Pericentrin; Integral component of the filamentous matrix of the centrosome involved in the initial establishment of organized microtubule arrays in both mitosis and meiosis. Plays a role, together with DISC1, in the microtubule network formation. Is an integral component of the pericentriolar material (PCM). May play an important role in preventing premature centrosome splitting during interphase by inhibiting NEK2 kinase activity at the centrosome |
| PIK3AP1 | Phosphoinositide 3-kinase adapter protein 1; Signaling adapter that contributes to B-cell development by linking B-cell receptor (BCR) signaling to the phosphoinositide 3-kinase (PI3K)-Akt signaling pathway. Has a complementary role to the BCR coreceptor CD19, coupling BCR and PI3K activation by providing a docking site for the PI3K subunit PIK3R1. Alternatively, links Toll-like receptor (TLR) signaling to PI3K activation, a process preventing excessive inflammatory cytokine production. Also involved in the activation of PI3K in natural killer cells. May be involved in the survival of [...] |
| PITX2 | Paired-like homeodomain transcription factor 2; Pituitary homeobox 2; Controls cell proliferation in a tissue-specific manner and is involved in morphogenesis. During embryonic development, exerts a role in the expansion of muscle progenitors. May play a role in the proper localization of asymmetric organs such as the heart and stomach. Isoform PTX2C is involved in left-right asymmetry the developing embryo (By similarity); Belongs to the paired homeobox family. Bicoid subfamily |
| PLCG2 | 1-phosphatidylinositol 4,5-bisphosphate phosphodiesterase gamma-2; The production of the second messenger molecules diacylglycerol (DAG) and inositol 1,4,5-trisphosphate (IP3) is mediated by activated phosphatidylinositol-specific phospholipase C enzymes. It is a crucial enzyme in transmembrane signaling; C2 domain containing phospholipases |
| PLEKHG1 | Pleckstrin homology domain-containing family g member 1; Pleckstrin homology and RhoGEF domain containing G1 |
| PLXNA2 | Plexin-A2; Coreceptor for SEMA3A and SEMA6A. Necessary for signaling by SEMA6A and class 3 semaphorins and subsequent remodeling of the cytoskeleton. Plays a role in axon guidance, invasive growth and cell migration. Class 3 semaphorins bind to a complex composed of a neuropilin and a plexin. The plexin modulates the affinity of the complex for specific semaphorins, and its cytoplasmic domain is required for the activation of down- stream signaling events in the cytoplasm (By similarity) |
| PPARGC1A | Peroxisome proliferator-activated receptor gamma coactivator 1-alpha; Transcriptional coactivator for steroid receptors and nuclear receptors. Greatly increases the transcriptional activity of PPARG and thyroid hormone receptor on the uncoupling protein promoter. Can regulate key mitochondrial genes that contribute to the program of adaptive thermogenesis. Plays an essential role in metabolic reprogramming in response to dietary availability through coordination of the expression of a wide array of genes involved in glucose and fatty acid metabolism. Induces the expression of PERM1 in [...] |
| PROSER2 | Proline and serine-rich protein 2; Proline and serine rich 2 |
| PRR5 | Proline-rich protein 5; Subunit of mTORC2, which regulates cell growth and survival in response to hormonal signals. mTORC2 is activated by growth factors, but, in contrast to mTORC1, seems to be nutrient- insensitive. mTORC2 seems to function upstream of Rho GTPases to regulate the actin cytoskeleton, probably by activating one or more Rho-type guanine nucleotide exchange factors. mTORC2 promotes the serum-induced formation of stress-fibers or F-actin. mTORC2 plays a critical role in AKT1 'Ser-473' phosphorylation, which may facilitate the phosphorylation of the activation loop of AKT [...] |
| PSMB1 | Proteasome subunit beta type-1; Component of the 20S core proteasome complex involved in the proteolytic degradation of most intracellular proteins. This complex plays numerous essential roles within the cell by associating with different regulatory particles. Associated with two 19S regulatory particles, forms the 26S proteasome and thus participates in the ATP-dependent degradation of ubiquitinated proteins. The 26S proteasome plays a key role in the maintenance of protein homeostasis by removing misfolded or damaged proteins that could impair cellular functions, and by removing prot [...] |
| PXDNL | Peroxidasin-like protein; Isoform PMR1: Endonuclease selectively degrading some target mRNAs while they are engaged by translating ribosomes, among which albumin and beta-globin mRNAs; I-set domain containing |
| RAB11FIP2 | Rab11 family-interacting protein 2; A Rab11 effector binding preferentially phosphatidylinositol 3,4,5-trisphosphate (PtdInsP3) and phosphatidic acid (PA) and acting in the regulation of the transport of vesicles from the endosomal recycling compartment (ERC) to the plasma membrane. Involved in insulin granule exocytosis. Also involved in receptor-mediated endocytosis and membrane trafficking of recycling endosomes, probably originating from clathrin-coated vesicles. Required in a complex with MYO5B and RAB11 for the transport of NPC1L1 to the plasma membrane. Also acts as a regulator [...] |
| RAD51B | DNA repair protein RAD51 homolog 2; Involved in the homologous recombination repair (HRR) pathway of double-stranded DNA breaks arising during DNA replication or induced by DNA-damaging agents. May promote the assembly of presynaptic RAD51 nucleoprotein filaments. Binds single-stranded DNA and double-stranded DNA and has DNA-dependent ATPase activity. Part of the RAD21 paralog protein complex BCDX2 which acts in the BRCA1-BRCA2-dependent HR pathway. Upon DNA damage, BCDX2 acts downstream of BRCA2 recruitment and upstream of RAD51 recruitment. BCDX2 binds predominantly to the intersecti [...] |
| RASGEF1A | Ras-GEF domain-containing family member 1A; Guanine nucleotide exchange factor (GEF) with specificity for RAP2A, KRAS, HRAS, and NRAS (in vitro). Plays a role in cell migration |
| RCC2 | Regulator of chromosome condensation 2; Protein RCC2; Required for completion of mitosis and cytokinesis. May function as a guanine nucleotide exchange factor for the small GTPase RAC1 |
| RFTN1 | Raftlin, lipid raft linker 1; Raftlin; May play a pivotal role in the formation and/or maintenance of lipid rafts. May regulate B-cell antigen receptor- mediated signaling |
| RGMA | Repulsive guidance molecule A; Member of the repulsive guidance molecule (RGM) family that performs several functions in the developing and adult nervous system. Regulates cephalic neural tube closure, inhibits neurite outgrowth and cortical neuron branching, and the formation of mature synapses. Binding to its receptor NEO1/neogenin induces activation of RHOA-ROCK1/Rho-kinase signaling pathway through UNC5B-ARHGEF12/LARG-PTK2/FAK1 cascade, leading to collapse of the neuronal growth cone and neurite outgrowth inhibition. Furthermore, RGMA binding to NEO1/neogenin leads to HRAS inactiva [...] |
| RNF4 | E3 ubiquitin-protein ligase RNF4; E3 ubiquitin-protein ligase which binds polysumoylated chains covalently attached to proteins and mediates 'Lys-6'-, 'Lys-11'-, 'Lys-48'- and 'Lys-63'-linked polyubiquitination of those substrates and their subsequent targeting to the proteasome for degradation. Regulates the degradation of several proteins including PML and the transcriptional activator PEA3. Involved in chromosome alignment and spindle assembly, it regulates the kinetochore CENPH-CENPI-CENPK complex by targeting polysumoylated CENPI to proteasomal degradation. Regulates the cellular [...] |
| ROCK1 | Rho-associated protein kinase 1; Protein kinase which is a key regulator of actin cytoskeleton and cell polarity. Involved in regulation of smooth muscle contraction, actin cytoskeleton organization, stress fiber and focal adhesion formation, neurite retraction, cell adhesion and motility via phosphorylation of DAPK3, GFAP, LIMK1, LIMK2, MYL9/MLC2, PFN1 and PPP1R12A. Phosphorylates FHOD1 and acts synergistically with it to promote SRC-dependent non-apoptotic plasma membrane blebbing. Phosphorylates JIP3 and regulates the recruitment of JNK to JIP3 upon UVB-induced stress. Acts as a sup [...] |
| RP1 | Oxygen-regulated protein 1; Microtubule-associated protein regulating the stability and length of the microtubule-based axoneme of photoreceptors. Required for the differentiation of photoreceptor cells, it plays a role in the organization of the outer segment of rod and cone photoreceptors ensuring the correct orientation and higher-order stacking of outer segment disks along the photoreceptor axoneme (By similarity); Doublecortin superfamily |
| RPAP2 | Putative RNA polymerase II subunit B1 CTD phosphatase RPAP2; Protein phosphatase that displays CTD phosphatase activity and regulates transcription of snRNA genes. Recognizes and binds phosphorylated 'Ser-7' of the C-terminal heptapeptide repeat domain (CTD) of the largest RNA polymerase II subunit POLR2A, and mediates dephosphorylation of 'Ser-5' of the CTD, thereby promoting transcription of snRNA genes |
| SLC25A21 | Mitochondrial 2-oxodicarboxylate carrier; Transports C5-C7 oxodicarboxylates across the inner membranes of mitochondria. Can transport 2-oxoadipate, 2- oxoglutarate, adipate, glutarate, and to a lesser extent, pimelate, 2-oxopimelate, 2-aminoadipate, oxaloacetate, and citrate; Belongs to the mitochondrial carrier (TC 2.A.29) family |
| SLC44A5 | Choline transporter-like protein 5; Solute carrier family 44 member 5; Belongs to the CTL (choline transporter-like) family |
| SLITRK1 | Slit and ntrk like family member 1; SLIT and NTRK-like protein 1; It is involved in synaptogenesis and promotes excitatory synapse differentiation. Enhances neuronal dendrite outgrowth |
| SMOX | Spermine oxidase; Flavoenzyme which catalyzes the oxidation of spermine to spermidine. Can also use N(1)-acetylspermine and spermidine as substrates, with different affinity depending on the isoform (isozyme) and on the experimental conditions. Plays an important role in the regulation of polyamine intracellular concentration and has the potential to act as a determinant of cellular sensitivity to the antitumor polyamine analogs. May contribute to beta-alanine production via aldehyde dehydrogenase conversion of 3-amino-propanal; Belongs to the flavin monoamine oxidase family |
| SNAP47 | Synaptosomal-associated protein 47; Plays a role in intracellular membrane fusion |
| SNRNP40 | U5 small nuclear ribonucleoprotein 40 kDa protein; Component of the U5 small nuclear ribonucleoprotein (snRNP) complex. The U5 snRNP is part of the spliceosome, a multiprotein complex that catalyzes the removal of introns from pre-messenger RNAs; Spliceosomal Bact complex |
| SORL1 | Sortilin-related receptor; Likely to be a multifunctional endocytic receptor, that may be implicated in the uptake of lipoproteins and of proteases. Binds LDL, the major cholesterol-carrying lipoprotein of plasma, and transports it into cells by endocytosis. Binds the receptor- associated protein (RAP). Could play a role in cell-cell interaction. Involved in APP trafficking to and from the Golgi apparatus. It probably acts as a sorting receptor that protects APP from trafficking to late endosome and from processing into amyloid beta, thereby reducing the burden of amyloidogenic peptide [...] |
| SRGAP2B | SLIT-ROBO Rho GTPase-activating protein 2B; May regulate cell migration and differentiation through interaction with and inhibition of SRGAP2; F-BAR domain containing |
| ST8SIA5 | Alpha-n-acetyl-neuraminate alpha-2,8-sialyltransferase (sialyltransferase 8e); Alpha-2,8-sialyltransferase 8E; May be involved in the synthesis of gangliosides GD1c, GT1a, GQ1b and GT3 from GD1a, GT1b, GM1b and GD3 respectively; Belongs to the glycosyltransferase 29 family |
| SUPT3H | Transcription initiation protein SPT3 homolog; Probable transcriptional activator; SAGA complex |
| SV2C | Mfs transporter, vnt family, synaptic vesicle glycoprotein 2; Synaptic vesicle glycoprotein 2C; Plays a role in the control of regulated secretion in neural and endocrine cells, enhancing selectively low-frequency neurotransmission. Positively regulates vesicle fusion by maintaining the readily releasable pool of secretory vesicles (By similarity) |
| TLK1 | Serine/threonine-protein kinase tousled-like 1; Rapidly and transiently inhibited by phosphorylation following the generation of DNA double-stranded breaks during S- phase. This is cell cycle checkpoint and ATM-pathway dependent and appears to regulate processes involved in chromatin assembly. Isoform 3 phosphorylates and enhances the stability of the t-SNARE SNAP23, augmenting its assembly with syntaxin. Isoform 3 protects the cells from the ionizing radiation by facilitating the repair of DSBs. In vitro, phosphorylates histone H3 at 'Ser-10' |
| TRAPPC9 | Trafficking protein particle complex subunit 9; Functions as an activator of NF-kappa-B through increased phosphorylation of the IKK complex. May function in neuronal cells differentiation. May play a role in vesicular transport from endoplasmic reticulum to Golgi; Belongs to the NIBP family |
| TWISTNB | DNA-directed RNA polymerase I subunit RPA43; DNA-dependent RNA polymerase catalyzes the transcription of DNA into RNA using the four ribonucleoside triphosphates as substrates. Component of RNA polymerase I which synthesizes ribosomal RNA precursors. Through its association with RRN3/TIF-IA may be involved in recruitment of Pol I to rDNA promoters; Belongs to the eukaryotic RPA43 RNA polymerase subunit family |
| UNC93A | Protein unc-93 homolog a; Unc-93 homolog A; Belongs to the unc-93 family |
| USH2A | Usherin; Involved in hearing and vision; Fibronectin type III domain containing |
| VPS13A | Vacuolar protein sorting-associated protein 13A; May play a role in the control of protein cycling through the trans-Golgi network to early and late endosomes, lysosomes and plasma membrane |
| XXYLT1 | Xyloside xylosyltransferase 1; Alpha-1,3-xylosyltransferase, which elongates the O- linked xylose-glucose disaccharide attached to EGF-like repeats in the extracellular domain of target proteins by catalyzing the addition of the second xylose. Known targets include Notch proteins and coagulation factors, such as F9; Glycosyltransferase family 8 |
| ZMYM4 | Zinc finger MYM-type protein 4; Plays a role in the regulation of cell morphology and cytoskeletal organization; Zinc fingers MYM-type |
| ZNF124 | Zinc finger protein 124; May be involved in transcriptional regulation; Belongs to the krueppel C2H2-type zinc-finger protein family |
| ZNF212 | Zinc finger protein 212; May be involved in transcriptional regulation; Zinc fingers C2H2-type |

Supplementary Table 17. Gene annotation table for Top genes in supervised clustering analysis of rare variants.

| Gene ID | Gene annotation |
| --- | --- |
| FAM47E-STBD1 | FAM47E-STBD1 readthrough |
| HLA-B | HLA class I histocompatibility antigen, B-7 alpha chain; Involved in the presentation of foreign antigens to the immune system; C1-set domain containing |
| KIAA1644 | Uncharacterized protein KIAA1644; KIAA1644 |
| LOC102724159 | annotation not available |
| MPHOSPH10 | U3 small nucleolar ribonucleoprotein protein MPP10; Component of the 60-80S U3 small nucleolar ribonucleoprotein (U3 snoRNP). Required for the early cleavages during pre-18S ribosomal RNA processing; MPP10 subcomplex |
| RHO | Rhodopsin; Photoreceptor required for image-forming vision at low light intensity. Required for photoreceptor cell viability after birth. Light-induced isomerization of 11-cis to all-trans retinal triggers a conformational change leading to G-protein activation and release of all-trans retinal; Belongs to the G-protein coupled receptor 1 family. Opsin subfamily |
| TROVE2 | 60 kDa SS-A/Ro ribonucleoprotein; RNA-binding protein that binds to misfolded non-coding RNAs, pre-5S rRNA, and several small cytoplasmic RNA molecules known as Y RNAs. May stabilize some of these RNAs and protect them from degradation; Belongs to the Ro 60 kDa family |
| RP11-301G19.1 | NA |
| RP11-359P18.1 | NA |
| RP11-568N6.1 | NA |
| RP4-671O14.7 | NA |
| RP11-1069G10.1 | NA |
| RP11-132N15.1 | NA |
| RP11-17M15.2 | NA |
| RP11-20J1.1 | NA |
| RP11-259G18.2 | NA |
| RP11-396C23.2 | NA |
| RP11-467M13.3 | NA |
| RP11-474D1.1 | NA |
| RP11-567J20.3 | NA |
| RP11-805I24.1 | NA |
| RP13-1016M1.2 | NA |
| RP3-400B16.1 | NA |
| RP4-620F22.3 | NA |
| CH507-9B2.5 | NA |
| CTA-109P11.1 | NA |
| CTB-113D17.1 | NA |
| CTC-264K15.6 | NA |
| CTC-529L17.2 | NA |
| DLGAP1-AS5 | NA |
| GS1-124K5.4 | NA |
| GS1-44D20.1 | NA |
| GS1-519E5.1 | NA |
| GS1-590J6.2 | NA |
| IGHV1OR15-9 | NA |
| ITGB5-AS1 | NA |
| KB-1090H4.2 | NA |
| KB-1410C5.1 | NA |
| KB-1410C5.3 | NA |
| KB-1930G5.4 | NA |
| LL22NC03-63E9.3 | NA |
| LMLN-AS1 | NA |
| NTM-IT | NA |
| RNU1-106P | NA |
| RNU1-108P | NA |
| RNU6-1101P | NA |
| RNU6-1151P | NA |
| RNU6-1293P | NA |
| RNU6-397P | NA |
| RNU6-858P | NA |
| RNU6-978P | NA |
| RNU7-12P | NA |
| RNU7-93P | NA |
| RP11-1003J3.1 | NA |
| RP11-107D24.2 | NA |
| RP11-10G15.4 | NA |
| RP11-112J3.16 | NA |
| RP11-1166P10.1 | NA |
| RP11-122G18.11 | NA |
| RP11-122G18.12 | NA |
| RP11-1250I15.3 | NA |
| RP11-131M6.1 | NA |
| RP11-133L19.2 | NA |
| RP11-139K4.2 | NA |
| RP11-157B13.8 | NA |
| RP11-159H10.1 | NA |
| RP11-177A2.5 | NA |
| RP11-187C18.4 | NA |
| RP11-197K6.1 | NA |
| RP11-217C7.1 | NA |
| RP11-21C4.4 | NA |
| RP11-226F19.1 | NA |
| RP11-239L20.6 | NA |
| RP11-247A12.2 | NA |
| RP11-252A24.8 | NA |
| RP11-252E2.2 | NA |
| RP11-255E6.5 | NA |
| RP11-258O13.1 | NA |
| RP11-269G24.8 | NA |
| RP11-292D4.4 | NA |
| RP11-2N5.2 | NA |
| RP11-30J20.1 | NA |
| RP11-316I3.1 | NA |
| RP11-321E2.9 | NA |
| RP11-326L9.1 | NA |
| RP11-340E6.1 | NA |
| RP11-341A22.1 | NA |
| RP11-357K9.2 | NA |
| RP11-359H3.1 | NA |
| RP11-359M6.2 | NA |
| RP11-368J21.4 | NA |
| RP11-370F5.4 | NA |
| RP11-395P16.1 | NA |
| RP11-396F22.1 | NA |
| RP11-396O20.2 | NA |
| RP11-403I13.7 | NA |
| RP11-426C22.6 | NA |
| RP11-429P3.5 | NA |
| RP11-431M7.3 | NA |
| RP11-459C13.1 | NA |
| RP11-472E5.3 | NA |
| RP11-473E2.2 | NA |
| RP11-474L11.5 | NA |
| RP11-4O3.2 | NA |
| RP11-511B23.1 | NA |
| RP11-522M21.3 | NA |
| RP11-523L1.2 | NA |
| RP11-53B5.1 | NA |
| RP11-542B15.1 | NA |
| RP11-556I14.1 | NA |
| RP11-55L3.1 | NA |
| RP11-567N4.3 | NA |
| RP11-578F21.2 | NA |
| RP11-580I1.1 | NA |
| RP11-599J14.2 | NA |
| RP11-59N23.1 | NA |
| RP11-603J24.9 | NA |
| RP11-63A2.2 | NA |
| RP11-648L3.1 | NA |
| RP11-651P23.4 | NA |
| RP11-66N5.2 | NA |
| RP11-699L21.1 | NA |
| RP11-705C15.2 | NA |
| RP11-708J19.3 | NA |
| RP11-71J2.1 | NA |
| RP11-72H11.1 | NA |
| RP11-757A13.1 | NA |
| RP11-769N21.2 | NA |
| RP11-787B4.2 | NA |
| RP11-800A18.4 | NA |
| RP11-813I20.2 | NA |
| RP11-86H7.6 | NA |
| RP11-89F3.2 | NA |
| RP11-972K6.1 | NA |
| RP13-140E4.1 | NA |
| RP13-313G19.2 | NA |
| RP3-363L9.1 | NA |
| RP3-449O17.1 | NA |
| RP3-463P15.1 | NA |
| RP3-492P14.2 | NA |
| RP4-580N22.2 | NA |
| RP5-1028K7.2 | NA |
| RP5-1136G13.1 | NA |
| RP5-1174N9.2 | NA |
| RP5-834N19.1 | NA |
| RP5-919F19.5 | NA |
| Y_RNA | NA |
| U3 | NA |
| AC008074.4 | NA |
| RP4 | NA |
| RP11-1069G10.2 | NA |
| RP11-430L16.1 | NA |
| RP11-17M15.4 | NA |
| RP11-336F14.1 | NA |
| RP11-259G18.3 | NA |
| RP11-396C23.3 | NA |
| RP11-14N9.2 | NA |
| RP11-474D1.2 | NA |
| RP11-567J20.2 | NA |
| RP11-805I24.4 | NA |
| RP11-1398P2.1 | NA |
| RP3-400B16.3 | NA |
| RP5-908D6.1 | NA |
| snoU13 | NA |
| RP11-768G3.1 | NA |

**References**

1. Research CfIBaMT: CIBMTR 2020 annual report. https://www.cibmtr.org/About/AdminReports/Documents/2020CIBMTRAnnualReport.pdf, 2020

2. Cooke DP, Wedge DC, Lunter G: A unified haplotype-based method for accurate and comprehensive variant calling. Nat Biotechnol 39:885-892, 2021

3. Karczewski KJ, Francioli LC, Tiao G, et al: The mutational constraint spectrum quantified from variation in 141,456 humans. Nature 581:434-443, 2020

4. Yang H, Wang K: Genomic variant annotation and prioritization with ANNOVAR and wANNOVAR. Nat Protoc 10:1556-66, 2015

5. Kircher M, Witten DM, Jain P, et al: A general framework for estimating the relative pathogenicity of human genetic variants. Nat Genet 46:310-5, 2014

6. Stenson PD, Mort M, Ball EV, et al: The Human Gene Mutation Database (HGMD((R))): optimizing its use in a clinical diagnostic or research setting. Hum Genet 139:1197-1207, 2020

7. Sondka Z, Bamford S, Cole CG, et al: The COSMIC Cancer Gene Census: describing genetic dysfunction across all human cancers. Nat Rev Cancer 18:696-705, 2018

8. Talevich E, Shain AH, Botton T, et al: CNVkit: Genome-Wide Copy Number Detection and Visualization from Targeted DNA Sequencing. PLoS Comput Biol 12:e1004873, 2016

9. Miller CA, White BS, Dees ND, et al: SciClone: inferring clonal architecture and tracking the spatial and temporal patterns of tumor evolution. PLoS Comput Biol 10:e1003665, 2014

10. Bergstrom EN, Huang MN, Mahto U, et al: SigProfilerMatrixGenerator: a tool for visualizing and exploring patterns of small mutational events. BMC Genomics 20:685, 2019

11. Duncavage EJ, Schroeder MC, Spencer DH: Genome Sequencing in Myeloid Cancers. Reply. N Engl J Med 384:e106, 2021

12. Haferlach T, Hutter S, Meggendorfer M: Genome Sequencing in Myeloid Cancers. N Engl J Med 384:e106, 2021

13. Cibulskis K, Lawrence MS, Carter SL, et al: Sensitive detection of somatic point mutations in impure and heterogeneous cancer samples. Nat Biotechnol 31:213-9, 2013

14. Jaksik R, Rosiak J, Zawadzki P, et al: Accuracy of somatic variant detection workflows for whole genome sequencing experiments. bioRxiv:2021.06.10.446467, 2021

15. Shin HT, Choi YL, Yun JW, et al: Prevalence and detection of low-allele-fraction variants in clinical cancer samples. Nat Commun 8:1377, 2017

16. Lindsley RC, Ebert BL: Molecular pathophysiology of myelodysplastic syndromes. Annu Rev Pathol 8:21-47, 2013

17. Sperling AS, Gibson CJ, Ebert BL: The genetics of myelodysplastic syndrome: from clonal haematopoiesis to secondary leukaemia. Nat Rev Cancer 17:5-19, 2017

18. Ogawa S: Genetics of MDS. Blood 133:1049-1059, 2019

19. Morris AP, Zeggini E: An evaluation of statistical approaches to rare variant analysis in genetic association studies. Genet Epidemiol 34:188-93, 2010

20. Cancer Genome Atlas Research N, Weinstein JN, Collisson EA, et al: The Cancer Genome Atlas Pan-Cancer analysis project. Nat Genet 45:1113-20, 2013

21. Lindsley RC, Saber W, Mar BG, et al: Prognostic Mutations in Myelodysplastic Syndrome after Stem-Cell Transplantation. N Engl J Med 376:536-547, 2017

22. Nazha A, Komrokji R, Meggendorfer M, et al: Personalized Prediction Model to Risk Stratify Patients With Myelodysplastic Syndromes. J Clin Oncol 39:3737-3746, 2021

23. McLaren W, Gil L, Hunt SE, et al: The Ensembl Variant Effect Predictor. Genome Biol 17:122, 2016

24. Ioannidis NM, Rothstein JH, Pejaver V, et al: REVEL: An Ensemble Method for Predicting the Pathogenicity of Rare Missense Variants. Am J Hum Genet 99:877-885, 2016

25. Dong C, Wei P, Jian X, et al: Comparison and integration of deleteriousness prediction methods for nonsynonymous SNVs in whole exome sequencing studies. Hum Mol Genet 24:2125-37, 2015

26. Adzhubei I, Jordan DM, Sunyaev SR: Predicting functional effect of human missense mutations using PolyPhen-2. Curr Protoc Hum Genet Chapter 7:Unit7 20, 2013

27. Shihab HA, Rogers MF, Gough J, et al: An integrative approach to predicting the functional effects of non-coding and coding sequence variation. Bioinformatics 31:1536-43, 2015

28. Shihab HA, Gough J, Cooper DN, et al: Predicting the functional, molecular, and phenotypic consequences of amino acid substitutions using hidden Markov models. Hum Mutat 34:57-65, 2013

29. Reva B, Antipin Y, Sander C: Predicting the functional impact of protein mutations: application to cancer genomics. Nucleic Acids Res 39:e118, 2011

30. Schwarz JM, Cooper DN, Schuelke M, et al: MutationTaster2: mutation prediction for the deep-sequencing age. Nat Methods 11:361-2, 2014

31. Adzhubei IA, Schmidt S, Peshkin L, et al: A method and server for predicting damaging missense mutations. Nat Methods 7:248-9, 2010

32. Vaser R, Adusumalli S, Leng SN, et al: SIFT missense predictions for genomes. Nat Protoc 11:1-9, 2016

33. Szklarczyk D, Gable AL, Nastou KC, et al: The STRING database in 2021: customizable protein-protein networks, and functional characterization of user-uploaded gene/measurement sets. Nucleic Acids Res 49:D605-D612, 2021
